# Giant Heterometallic [Mn_36_Ni_4_]^0/2−^ and [Mn_32_Co_8_] “Loops-of-Loops-and-Supertetrahedra” Molecular Aggregates

**DOI:** 10.3389/fchem.2019.00096

**Published:** 2019-03-05

**Authors:** Maria Charalambous, Eleni E. Moushi, Tu N. Nguyen, Constantina Papatriantafyllopoulou, Vassilios Nastopoulos, George Christou, Anastasios J. Tasiopoulos

**Affiliations:** ^1^Department of Chemistry, University of Cyprus, Nicosia, Cyprus; ^2^Department of Chemistry, University of Florida, Gainesville, FL, United States; ^3^Department of Chemistry, University of Patras, Patras, Greece

**Keywords:** Mn, heterometallic clusters, diols, magnetic properties, crystal structures

## Abstract

We report the synthesis, crystal structures and magnetic properties of the giant heterometallic [Mn_36_Ni_4_]^2−/0^ (compounds **1**, **2**)/[Mn_32_Co_8_] (compound **3**) “loops-of-loops-and-supertetrahedra” molecular aggregates and of a [Mn_2_Ni_6_]^2+^ compound (cation of **4**) that is structurally related with the cation co-crystallizing with the anion of **1**. In particular, after the initial preparation and characterization of compound [Mn_2_Ni_6_(μ_4_-O)_2_(μ_3_-OH)_3_(μ_3_-Cl)_3_(O_2_CCH_3_)_6_(py)_8_]^2+^[Mn_36_Ni_4_(μ_4_-O)_8_(μ_3_-O)_4_(μ_3_-Cl)_8_Cl_4_(O_2_CCH_3_)_26_(pd)_24_(py)_4_]^2−^ (**1**) we targeted the isolation of (i) both the cationic and the anionic aggregates of **1** in a discrete form and (ii) the Mn/Co analog of [Mn_36_Ni_4_]^2−^ aggregate. Our synthetic efforts toward these directions afforded the discrete [Mn_36_Ni_4_] “loops-of-loops-and-supertetrahedra” aggregate [Mn_36_Ni_4_(μ_4_-O)_8_(μ_3_-O)_4_(μ_3_-Cl)_8_Cl_2_(O_2_CCH_3_)_26_(pd)_24_(py)_4_(H_2_O)_2_] (**2**), the heterometallic Mn/Co analog [Mn_32_Co_8_(μ_4_-O)_8_(μ_3_-O)_4_(μ_3_-Cl)_8_Cl_2_(μ_2_-OCH_2_CH_3_)_2_(O_2_CCH_3_)_28_(pd)_22_(py)_6_] (**3**) and the discrete [Mn_2_Ni_6_]^2+^ cation [Mn_2_Ni_6_(μ_4_-O)_2_(μ_3_-OH)_4_(μ_3_-Cl)_2_(O_2_CCH_3_)_6_(py)_8_](ClO_4_)(OH) (**4**). The structure of **1** consists of a mixed valence [${\text{Mn}}_{28}^{\text{III}}$${\text{Mn}}_{8}^{\text{II}}$${\text{Ni}}_{4}^{\text{II}}$]^2−^ molecular aggregate that contains two ${\text{Mn}}_{8}^{\text{III}}$${\text{Ni}}_{2}^{\text{II}}$ loops separated by two ${\text{Mn}}_{6}^{\text{III}}$${\text{Mn}}_{4}^{\text{II}}$ supertetrahedral units and a [${\text{Mn}}_{2}^{\text{III}}$${\text{Ni}}_{6}^{\text{II}}$]^2+^ cation based on two [Mn^III^${\text{Ni}}_{3}^{\text{II}}$(μ_4_-O)(μ_3_-OH)_1.5_(μ_3_-Cl)_1.5_]^4+^ cubane sub-units connected through both mono- and tri-atomic bridges provided by the μ_4_-O^2−^ and carboxylate anions. The structures of **2**–**4** are related to those of the compounds co-crystallized in **1** exhibiting however some differences that shall be discussed in detail in the manuscript. Magnetism studies revealed the presence of dominant ferromagnetic interactions in **1**–**3** that lead to large ground state spin (S_T_) values for the “loops-of-loops-and-supertetrahedra” aggregates and antiferromagnetic exchange interactions in **4** that lead to a low (and possibly zero) S_T_ value. In particular, dc and ac magnetic susceptibility studies revealed that the discrete [Mn_36_Ni_4_] aggregate exhibits a large S_T_ value ~ 26 but is not a new SMM. The ac magnetic susceptibility studies of the [Mn_32_Co_8_] analog revealed an extremely weak beginning of an out-of-phase tail indicating the presence of a very small relaxation barrier assignable to the anisotropic Co^2+^ions and a resulting out-of-phase ac signal whose peak is at very low T.

## Introduction

High nuclearity Mn carboxylate clusters continue to attract significant attention mainly because of their structural characteristics and physical properties (Bagai and Christou, 2009; Kostakis et al., 2010; Escuer et al., 2014). In particular, such compounds often exhibit interesting magnetic properties including high spin ground state values (Ako et al., 2006; Moushi et al., 2009) and single—molecule magnetism behavior (Sessoli et al., 1993; Bagai and Christou, 2009; Inglis et al., 2012; Milios and Winpenny, 2015). The latter appears in molecules exhibiting a large spin ground state (S_T_) value and significant easy axis magnetoanisotropy (Christou et al., 2000; Nakano and Oshio, 2011; Ferrando-Soria et al., 2017). In addition, Mn complexes have attracted significant attention since they are involved in the search for structural and functional analogs of the tetranuclear Mn complex that is present in the active site of photosystem II and is responsible for the photosynthetic oxidation of H_2_O to molecular O_2_ (Mukherjee et al., 2012; Yano and Yachandra, 2014; Gerey et al., 2016). Thus, Mn clusters have been proposed for various applications in diverse areas including magnetic refrigeration (Zheng et al., 2014), molecular spintronics (Bogani, 2015), quantum computation (Aromí et al., 2012), and catalysis (Maayan et al., 2018).

This interest has resulted in the development of several synthetic methods to Mn carboxylate clusters and the isolation of numerous high nuclearity complexes possessing a wide variety of shapes (wheels, disks, icosahedra, cuboctahedra, spheres, rods, etc.) and nuclearities (Kostakis et al., 2010). Some of these compounds exhibit very high nuclearities and dimensions with the list of giant, nanosized Mn clusters containing [Mn_29_] (Alexandropoulos et al., 2016), [Mn_30_] (Soler et al., 2004), [Mn_31_] (Abbasi et al., 2017), [Mn_32_] (Scott et al., 2005; Langley et al., 2011; Manoli et al., 2011), [Mn_40_Na_4_] (Moushi et al., 2007, 2010a), [Mn_44_] (Moushi et al., 2010a), [Mn_49_] (Manoli et al., 2016), [Mn_70_] (Vinslava et al., 2016), and [Mn_84_] (Tasiopoulos et al., 2004) aggregates. Although there are several homometallic nanosized Mn clusters reported, a [Mn_28_Cu_17_] aggregate is the only example of giant heterometallic Mn/M (M = any paramagnetic metal ion) compounds (Wang et al., 2007). Apart from their exciting crystal structures some of these nanosized aggregates exhibit interesting magnetic properties. For example, compounds [Mn_30_], [Mn_31_], [Mn_32_], [Mn_70_], and [Mn_84_] possess SMM behavior with an appreciable energy barrier to magnetization reorientation and represent a meeting of the bottom-up and top-down approaches to nanomagnetism (Papatriantafyllopoulou et al., 2016). In addition, compounds [Mn_49_] and [Mn_28_Cu_17_] display dominant ferromagnetic exchange interactions leading to giant S_T_ values S = 61/2 and 51/2, respectively (Wang et al., 2007; Manoli et al., 2016). Note that the record S_T_ values have appeared in other giant homometallic Fe_42_ (Kang et al., 2015) and heterometallic [Ni_21_Gd_20_] (Chen et al., 2018) clusters and are S_T_ = 45 and 91, respectively. Interestingly although Mn cluster chemistry has proven to be the most fruitful source of giant metal clusters among other 3d metal ions there is only one heterometallic Mn/M (M = any metal ion) reported in contrast to the situation with other metal ions. For example, in Ni^2+^ or Cu^2+^ chemistry there are only a few giant homometallic clusters but there are several hetemetallic ones and especially Ni^2+^/4f and Cu^2+^/4f aggregates (Papatriantafyllopoulou et al., 2016).

Our group has been exploring reactions of diols with Mn—containing precursor compounds targeting to new high nuclearity Mn clusters and SMMs (Tasiopoulos and Perlepes, 2008). These investigations have afforded a series of giant Mn carboxylate clusters including [Mn_25_Na_4_] and [Mn_49_] aggregates consisting of eight and four decametallic supertetrahedral repeating sub-units (Manoli et al., 2016). Note that discrete metal clusters exhibiting a [${\text{Mn}}_{6}^{\text{III}}$${\text{Mn}}_{4}^{\text{II}}$(μ_4_-O)_4_]^18+^ supertetrahedral core analogous to that appeared in [Mn_25_Na_4_] and [Mn_49_] aggregates have been stabilized in several cases, especially with polyol-type ligands and in most cases these compounds exhibited entirely ferromagnetic exchange interactions and S_T_ = 22 (Stamatatos et al., 2006; Manoli et al., 2007, 2008; Wu et al., 2011). The use of 1, 3-propanediol (pdH_2_) and its derivatives in Mn carboxylate chemistry afforded a family of [Mn_44_] and [Mn_40_Na_4_] loops consisting of four [Mn_10_M(μ_3_- O)_2_(O_2_CCH_3_)_13_(pd)_6_(py)_2_]${\;}_{4}^{\text{x}+}$ ([Mn_40_M_4_]; M = Na^+^, x = 0; M = Mn^2+^, x = 1) loops linked through Na^+^ or Mn^2+^ ions (called “loops-of-loops”) and have a saddle-like topology. The [Mn_44_] analog of this family displays a spin *S*_T_ = 6 ground state and SMM behavior (Moushi et al., 2007, 2010a).

Further investigation of the reactions that afforded the [Mn_40_M_4_] loops-of-loops aggregates involved the use of various 3d paramagnetic metal ions in an attempt to isolate a series of heterometallic Mn/3d analogs and/or other large aggregates composed of smaller clusters. These investigations afforded compounds [Mn_2_Ni_6_(μ_4_-O)_2_(μ_3_-OH)_3_(μ_3_-Cl)_3_(O_2_CCH_3_)_6_(py)_8_]^2+^[Mn_36_Ni_4_(μ_4_-O)_8_(μ_3_-O)_4_(μ_3_-Cl)_8_Cl_4_(O_2_CCH_3_)_26_(pd)_24_(py)_4_]^2−^ (**1**) [Mn_36_Ni_4_(μ_4_-O)_8_(μ_3_-O)_4_(μ_3_-Cl)_8_Cl_2_(O_2_CCH_3_)_26_(pd)_24_(py)_4_(H_2_O)_2_] (**2**) and [Mn_32_Co_8_(μ_4_-O)_8_(μ_3_-O)_4_(μ_3_-Cl)_8_Cl_2_(μ_2_-OCH_2_CH_3_)_2_(O_2_CCH_3_)_28_(pd)_22_(py)_6_] (**3**). The last compound discussed herein is the discrete [Mn_2_Ni_6_]^2+^ cation [Mn_2_Ni_6_(μ_4_-O)_2_(μ_3_-OH)_4_(μ_3_-Cl)_2_(O_2_CCH_3_)_6_(py)_8_](ClO_4_)(OH) (**4**), i.e., an analog of the complex co-crystallizing with the [Mn_36_Ni_4_]^2−^ anion of **1**. Compounds **1**–**3** are rare examples of giant heterometallic Mn/M clusters and possess an unprecedented “loop-of-loops-and-supertetrahedra” structural topology. Complexes **2** and **3** exhibit dominant ferromagnetic exchange interactions and large S_T_ values which in the case of **2** is 26 ± 1. The [Mn_36_Ni_4_] aggregates (compounds **1** and **2**) do not display SMM behavior as is also the case for their [${\text{Mn}}_{6}^{\text{III}}$${\text{Mn}}_{4}^{\text{II}}$(μ_4_-O)_4_]^18+^ supertetrahedral sub-unit, whereas for their [Mn_32_Co_8_] analog (complex **3**) the existence of an out-of-phase tail is an indication of SMM behavior, however, further studies are required to confirm this conclusion. Part of this work, involving the synthesis and characterization of compound **2** has been communicated previously (Charalambous et al., 2012).

## Materials and Methods

### Materials and Physical Measurements

All manipulations were performed under aerobic conditions using chemicals and solvents as received, unless otherwise stated. [Mn_3_O(O_2_CCH_3_)_6_(py)_3_]·py was prepared as previously described (Vincent et al., 1987).

IR spectra were recorded in the solid state (KBr pellets) in the 4,000–400 cm^−1^ range using a Shimadzu Prestige−21 spectrometer. Elemental analysis (C, H, and N) were performed by the in-house facilities of the Chemistry Department at the University of Florida.

Variable-temperature dc magnetic susceptibility data down to 1.80 K were collected on a Quantum Design MPMS-XL SQUID magnetometer equipped with a 70 kG (7 T) dc magnet at the University of Florida. Diamagnetic corrections were applied to the observed paramagnetic susceptibilities using Pascal's constants. Samples were embedded in solid eicosane to prevent torquing. AC magnetic susceptibility data were collected on the same instrument employing a 3.5 G AC field oscillating at frequencies up to 1,500 Hz. Magnetization vs. field and temperature data were fit using the program MAGNET (Davidson, E. R.)^1^.

### Experimental

#### [Mn_2_Ni_6_(μ_4_-O)_2_(μ_3_-OH)_3_(μ_3_-Cl)_3_(O_2_CCH_3_)_6_(py)_8_]^2+^ [Mn_36_ Ni_4_(μ_4_-O)_8_(μ_3_-O)_4_(μ_3_-Cl)_8_Cl_4_(O_2_CCH_3_)_26_ (pd)_24_(py)_4_]^2−^·6H_2_O, (1)·6H_2_O

To a stirred brown solution of [Mn_3_O(O_2_CCH_3_)_6_(py)_3_]·py (0.23 g, 0.27 mmol) in 15 ml CH_3_CN were added pdH_2_ (100 μL, 0.105 g, 1.38 mmol) and solid NiCl_2_·6H_2_O (0.066 g, 0.27 mmol). The reaction mixture was left under magnetic stirring for 10 min, filtered off and the filtrate was left undisturbed at room temperature. After 1 week brown X-ray quality crystals of **1** suitable for X-ray structural determination were formed. The crystals were isolated by filtration, washed with CH_3_CN and dried in vacuo; the yield was ~36%. The crystals for X-ray studies were maintained in contact with mother liquor to prevent solvent loss. % C H N Anal. for C_196_H_315_N_12_O_135_Cl_15_Mn_38_Ni_10_ [(**1**)·6H_2_O]: calcd: C 28.69, H 3.87, N 2.05; found: C 29.02, H 3.75, N 2.35. Metal analysis was performed via ICP-OES. Anal. Calc. for (**1**)·6H_2_O: Mn 25.44, Ni 7.15; found: Mn 25.64, Ni 7.38%. Selected IR data (cm^−1^, KBr pellet): 3,420 (s, br), 2,931 (m), 2,854 (m), 1,560 (s, br), 1,419 (s, br), 1,341 (w), 1,087 (s), 951 (m), 835 (w), 637 (s, br).

#### [Mn_36_Ni_4_(μ_4_-O)_8_(μ_3_-O)_4_(μ_3_-Cl)_8_Cl_2_(O_2_CCH_3_)_26_(pd)_24_ (py)_4_(H_2_O)_2_]·10H_2_O, (2)·10H_2_O

To a brown solution of [Mn_3_O(O_2_CCH_3_)_6_(py)_3_]·py (0.23 g, 0.27 mmol) in 15 mL CH_3_CN were added under magnetic stirring pdH_2_ (200 μL, 0.211 g, 2.77 mmol) and NiCl_2_·6H_2_O (0.066 g, 0.27 mmol). The reaction mixture was left under magnetic stirring at room temperature for ~1 h and then undisturbed for ~3 h. The resulting dark brown slurry was filtered off and the filtrate was left undisturbed at room temperature. After a few days X-ray quality crystals of **2**·2CH_3_CN·12.30H_2_O were formed, isolated by filtration, washed with CH_3_CN and dried under vacuum. The crystals for X-ray studies were maintained in contact with mother liquor to prevent solvent loss. The yield was 35% based on total Mn content. % C H N Anal. for C_144_H_266_N_4_O_124_Cl_10_Mn_36_Ni_4_ [(**2**)·10H_2_O]: calcd: C 26.19, H 4.06, N 0.85; found: C 26.32, H 4.09, N 0.94. Metal analysis was performed via ICP-OES. Anal. Calc. for (**2**)·10H_2_O: Mn 29.94, Ni 3.55; found: Mn 30.09, Ni 3.68%. Selected IR data (cm^−1^, KBr pellet): 3,426 (s, br), 2,934 (m), 2,849 (m), 1,593 (s, br), 1,553 (w), 1,404 (m), 1,085 (s), 945 (w), 629 (s, br).

#### [Mn_32_Co_8_(μ_4_-O)_8_(μ_3_-O)_4_(μ_3_-Cl)_8_Cl_2_(μ_2_-OCH_2_CH_3_)_2_ (O_2_CCH_3_)_28_(pd)_22_(py)_6_]·20H_2_O, (3)·20H_2_O

To a stirred brown solution of [Mn_3_O(O_2_CCH_3_)_6_(py)_3_]·py (0.23 g, 0.27 mmol) in 12 ml of EtOH were added pdH_2_ (300 μL, 0.316 g, 4.15 mmol) and solid CoCl_2_·6H_2_O (0.066 g, 0.27 mmol) and the reaction mixture was left under magnetic stirring for 2 h. The resulting red-brown slurry was filtered off and the filtrate was layered with Et_2_O (1:3 v/v). After 2 weeks brown crystals of (**3**)·3.84 EtOH·6H_2_O were formed suitable for X-ray structural determination. The crystals were isolated by filtration, washed with EtOH and dried in vacuo; the yield was 29%. The crystals for X-ray studies were maintained in contact with mother liquor to prevent solvent loss. % C H N Anal. for C_156_H_296_N_6_O_134_Cl_10_Mn_32_Co_8_ [(**3**)·20H_2_O]: calcd: C 26.83, H 4.27, N 1.20; found: C 26.55, H 4.01, N 0.97. Metal analysis was performed via ICP-OES. Anal. Calc. for (**3**)·20H_2_O: Mn 25.17, Co 6.75; found: Mn 25.38, Co 6.88%. Selected IR data (cm^−1^, KBr pellet): 3,414 (s, br), 3,110 (s, br), 2,843 (w), 2,770 (w), 1,610 cm^−1^ (m), 1,556 (w), 1396 (s), 1,078 (m), 621(m).

#### [Mn_2_Ni_6_(μ_4_-O)_2_(μ_3_-OH)_4_(μ_3_-Cl)_2_(O_2_CCH_3_)_6_(py)_8_] (ClO_4_)(OH)·2H_2_O, (4)·2H_2_O

To a stirred brown solution of [Mn_3_O(O_2_CCH_3_)_6_(py)_3_]·py (0.20 g, 0.24 mmol) in 15 mL EtOH were added solids NiCl_2_·6H_2_O (0.06 g, 0.24 mmol) and NaClO_4_(0.03 g, 0.24 mmol) and the reaction mixture was left under magnetic stirring at room temperature for 2 h. The resulting brown slurry was filtered off and the dark brown filtrate was layered with Et_2_O (1:3 v/v) and left undisturbed at room temperature for a period of 1 week, upon which yellow-brown crystals of **4** suitable for X-ray structural determination were formed. The crystals were isolated by filtration, washed with EtOH and dried in vacuo; the yield was ~21%. C H N Anal. for C_52_H_67_N_8_O_25_Cl_3_Mn_2_Ni_6_ [(**4**)·2H_2_O]: calcd: C 35.24, H 3.81, N 6.32; found: C 35.54, H 3.55, N 6.74. Metal analysis was performed via ICP-OES. Anal. Calc. for (**4**)·2H_2_O: Mn 6.20, Ni 19.87; found: Mn 6.52, Ni 20.28 %. Selected IR data (cm^−1^, KBr pellet): 3,549 (w, br), 3,078 (w, br), 1,584 (s), 1,443 (s), 1,413 (s), 1,223 (m), 1,105 (s), 698 (s), 627 (m).

### Single Crystal X-Ray Crystallography

Single crystal X-ray diffraction data for (**1**), (**2**)·2CH_3_CN·12.30H_2_O, (**3**)·3.84EtOH·6H_2_O, and (**4**) were collected on an Oxford-Diffraction Supernova diffractometer, equipped with a CCD detector utilizing Mo Ka (λ = 0.71073 Å) radiation. A suitable crystal was mounted on a Hampton cryoloop with Paratone-N oil and transferred to a goniostat where it was cooled for data collection. Empirical absorption corrections (multiscan based on symmetry-related measurements) were applied using CrysAlis RED software (Oxford Diffraction, 2008). The structures were solved by direct methods using SIR2004 (Burla et al., 2005) and refined on F^2^ using full-matrix least-squares with SHELXL-2014/7 (Sheldrick, 2014) Software packages used were as follows: CrysAlis CCD for data collection (Oxford Diffraction 2008). CrysAlis RED for cell refinement and data reduction (Oxford Diffraction 2008). WINGX for geometric calculations (Farrugia, 1999), and DIAMOND (Brandenburg, 2006) for molecular graphics. The non-H atoms were treated anisotropically, whereas the aromatic H atoms were placed in calculated, ideal positions and refined as riding on their respective carbon atoms. Electron density contributions from disordered guest molecules were handled using the SQUEEZE procedure from the PLATON software suit (Van der Sluis and Spek, 1990; Spek, 2003). Selected crystal data for (**1**), (**2**)·2CH_3_CN·12.30H_2_O, (**3**)·3.84 EtOH·6H_2_O, and (**4**) are summarized in Table S1, whereas selected bond lengths and angles are given in Tables S2–S5.

CCDC 1859793, CCDC 862029, CCDC 1859811, and CCDC 1859814 contain the supplementary crystallographic data for (**1**), (**2**)·2CH_3_CN·12.30H_2_O, (**3**)·3.84 EtOH·6H_2_O, and (**4**), respectively. These data can be obtained free of charge from The Cambridge Crystallographic Data Centre via www.ccdc.cam.ac.uk/data_request/cif.

## Results

### Syntheses

We have been systematically studying reactions of Mn salts and preformed clusters with diols as a route to new polynuclear clusters with novel structural characteristics and interesting magnetism (Tasiopoulos and Perlepes, 2008; Moushi et al., 2009, 2010b; Skordi et al., 2018). These studies have focused on the use of simple aliphatic diols such as pdH_2_ and its derivatives which due to their alkoxide arms exhibit a high bridging capability and a fruitful coordination chemistry. We recently reported a family of large molecular aggregates consisting of four smaller clusters linked through Na^+^ or Mn^2+^ ions (Moushi et al., 2010a). These large tetrameric {[Mn_10_M(μ_3_- O)_2_(O_2_CCH_3_)_13_(pd)_6_(py)_2_]_4_}^x+^ ([Mn_40_M_4_]; M = Na^+^, x = 0; M = Mn^2+^, x = 1), clusters contain four Mn_10_ loops linked through Na^+^ or Mn^2+^ ions and have a saddle-like topology. They were prepared from reactions of [Mn_3_O(O_2_CMe)_6_(py)_3_]·py (py = pyridine) with H_2_pd in the presence of NaN_3_ [Mn_40_Na_4_] or Mn(ClO_4_)_2_·6H_2_O [Mn_44_]. We were interested to extend this study by preparing analogous heterometallic Mn_x_M_y_ (M = a 3d metal ion) complexes. These studies involved the investigation of similar reactions to those led to the [Mn_44_] or [Mn_40_Na_4_] aggregates which however, in the place of Mn(ClO_4_)_2_·6H_2_O or NaN_3_ contained a 3d metal ion salt. The initial result of these studies was compound [Mn_2_Ni_6_(μ_4_-O)_2_(μ_3_-OH)_3_(μ_3_-Cl)_3_(O_2_CCH_3_)_6_(py)_8_]^2+^[Mn_36_Ni_4_(μ_4_-O)_8_(μ_3_-O)_4_(μ_3_-Cl)_8_Cl_4_(O_2_CCH_3_)_26_(pd)_24_(py)_4_]^2−^ (**1**) that was obtained in ~36% yield from the reaction of [Mn_3_O(O_2_CCH_3_)_6_(py)_3_]·py with 1,3-propanediol (pdH_2_) in the presence of NiCl_2_·6H_2_O in 1:5:1 molar ratio in CH_3_CN. The formation of compound **1** is summarized in Equation (1):

(1) $$\begin{array}{l} 38[{\text{Mn}}_{3}\text{O}{\left({\text{O}}_{2}{\text{CCH}}_{3}\right)}_{6}{\left(\text{py}\right)}_{3}]\cdot \text{py}+72{\text{H}}_{2}\text{L}+30{\text{NiCl}}_{2}\cdot 6{\text{H}}_{2}\text{O}\\ +7/2{\text{O}}_{2}\overset{{\text{CH}}_{3}\text{CN}}{\to }3{\left[Mn_{36}Ni_{4}O_{12}Cl_{12}{\left(O_{2}CCH_{3}\right)}_{26}{\left(pd\right)}_{24}{\left(py\right)}_{4}\right]}^{2-}\\ {\left[Mn_{2}Ni_{6}O_{2}{\left(OH\right)}_{3}{\left(Cl\right)}_{3}{\left(O_{2}CCH_{3}\right)}_{6}{\left(py\right)}_{8}\right]}^{2+}\\ +15\text{HCl}+\;116\text{py}+\;132{\text{CH}}_{3}\text{COOH}+174{\text{H}}_{2}\text{O} \end{array}$$

Since compound **1** crystallized as a mixture of a cationic and an anionic complexes, we targeted the isolation of both of its components, and especially of [Mn_36_Ni_4_] aggregate, in a discrete form. From the molecular formula of **1** it was observed that in the [Mn_2_Ni_6_]^2+^ cation of **1** there are no pd^2−^ ligands in contrast to the [Mn_36_Ni_4_]^2−^ anion of **1** which contains 24 pd^2−^ groups. We thus decided to increase the ratio of pd^2−^ in the reaction mixture in order to facilitate the formation of the complex that contains pd^2−^ groups and this modification resulted in the isolation of the discrete [Mn_36_Ni_4_] analog. In particular, the reaction of [Mn_3_O(O_2_CCH_3_)_6_(py)_3_]·py with pdH_2_ and NiCl_2_·6H_2_O in a 1:10:1 molar ratio in CH_3_CN afforded the discrete [Mn_36_Ni_4_] complex [Mn_36_Ni_4_O_12_Cl_10_(O_2_CCH_3_)_26_(pd)_24_(py)_4_(H_2_O)_2_]·10H_2_O in 35% yield. The formation of compound **2** is summarized in Equation (2):

(2) $$\begin{array}{l} 12[{\text{Mn}}_{3}\text{O}{\left({\text{O}}_{2}\text{CMe}\right)}_{6}{\left(\text{py}\right)}_{3}]\cdot \text{py}+24{\text{H}}_{2}\text{pd}+5{\text{NiCl}}_{2}\cdot 6{\text{H}}_{2}\text{O}\\ +{\text{O}}_{2}\overset{{\text{CH}}_{3}\text{CN}}{\to }[Mn_{36}Ni_{4}O_{12}Cl_{10}{\left(O_{2}\;CCH_{3}\right)}_{26}{\left(pd\right)}_{24}{\left(py\right)}_{4}\\ {\left(H_{2}O\right)}_{2}]+44\text{py}+44{\text{CH}}_{3}\text{COOH}+\text{Ni}{\left({\text{CH}}_{3}\text{COO}\right)}_{2}+30{\text{H}}_{2}\text{O} \end{array}$$

After the isolation of compound **2** was realized, our efforts were focused on the synthesis of other Mn_40−x_M_x_ (M = a 3d metal ion) heterometallic “loops-of-loops-and-supertetrahedra” molecular aggregates and also of the [Mn_2_Ni_6_] cation of **1** in a discrete form. The preparation of a Mn_40−x_Co_x_ analog was our initial target since the incorporation in this structure of the highly anisotropic Co^2+^ ions could result in the appearance of a different magnetic behavior in the resulting compound than those shown in **1** and **2**. The formation of the Mn/Co species was achieved from the reaction of [Mn_3_O(O_2_CCH_3_)_6_(py)_3_]·py with pdH_2_ and CoCl_2_·6H_2_O in a 1:15:1 molar ratio in C_2_H_5_OH in ~29% yield. The formation of compound **3** is summarized in Equation (3):

(3) $$\begin{array}{l} 32[{\text{Mn}}_{3}\text{O}{\left({\text{O}}_{2}{\text{CCH}}_{3}\right)}_{6}{\left(\text{py}\right)}_{3}]\cdot \text{py}+66{\text{H}}_{2}\text{pd}+24{\text{CoCl}}_{2}\cdot 4{\text{H}}_{2}\text{O}\\ +6{\text{CH}}_{3}{\text{CH}}_{2}\text{OH}+5{\text{O}}_{2}\overset{{\text{CH}}_{3}{\text{CH}}_{2}\text{OH}}{\to }3[Mn_{32}Co_{8}O_{12}Cl_{10}\\ {\left(OCH_{2}CH_{3}\right)}_{2}{\left(O_{2}CCH_{3}\right)}_{28}{\left(pd\right)}_{22}{\left(py\right)}_{6}]+18\text{HCl}+110\text{py}\\ +108{\text{CH}}_{3}\text{COOH}+102{\text{H}}_{2}\text{O} \end{array}$$

The discrete [Mn_2_Ni_6_] cluster was also isolated by following a similar synthetic procedure to the one that led to the discrete [Mn_36_Ni_4_] aggregate which however, did not involve the use of pdH_2_ which is not present in this compound. Thus, the reaction of [Mn_3_O(O_2_CCH_3_)_6_(py)_3_]·py with NiCl_2_·6H_2_O in the presence of NaClO_4_ in a 1:1:1 molar ratio in C_2_H_5_OH afforded complex **4** in ~21% yield. The formation of compound **4** is summarized in Equation (4):

(4) $$\begin{array}{l} \left[{\text{Mn}}_{3}\text{O}{\left({\text{O}}_{2}{\text{CCH}}_{3}\right)}_{6}{\left(\text{py}\right)}_{3}]\cdot \text{py}+6{\text{NiCl}}_{2}\cdot 6{\text{H}}_{2}\text{O}+3\text{Na}({\text{ClO}}_{4}\right)\\ +4\text{py}\overset{{\text{CH}}_{3}{\text{CH}}_{2}\text{OH}}{\to }[Mn_{2}Ni_{6}O_{2}{\left(OH\right)}_{4}{\left(Cl\right)}_{2}-\\ {\left(O_{2}CCH_{3}\right)}_{6}{\left(py\right)}_{8}](ClO_{4})(OH)+7\text{HCl}+3\text{NaCl}\\ +\text{Mn}{\left({\text{ClO}}_{4}\right)}_{2}+30{\text{H}}_{2}\text{O} \end{array}$$

### Description of the Structures

**Figure 1:**
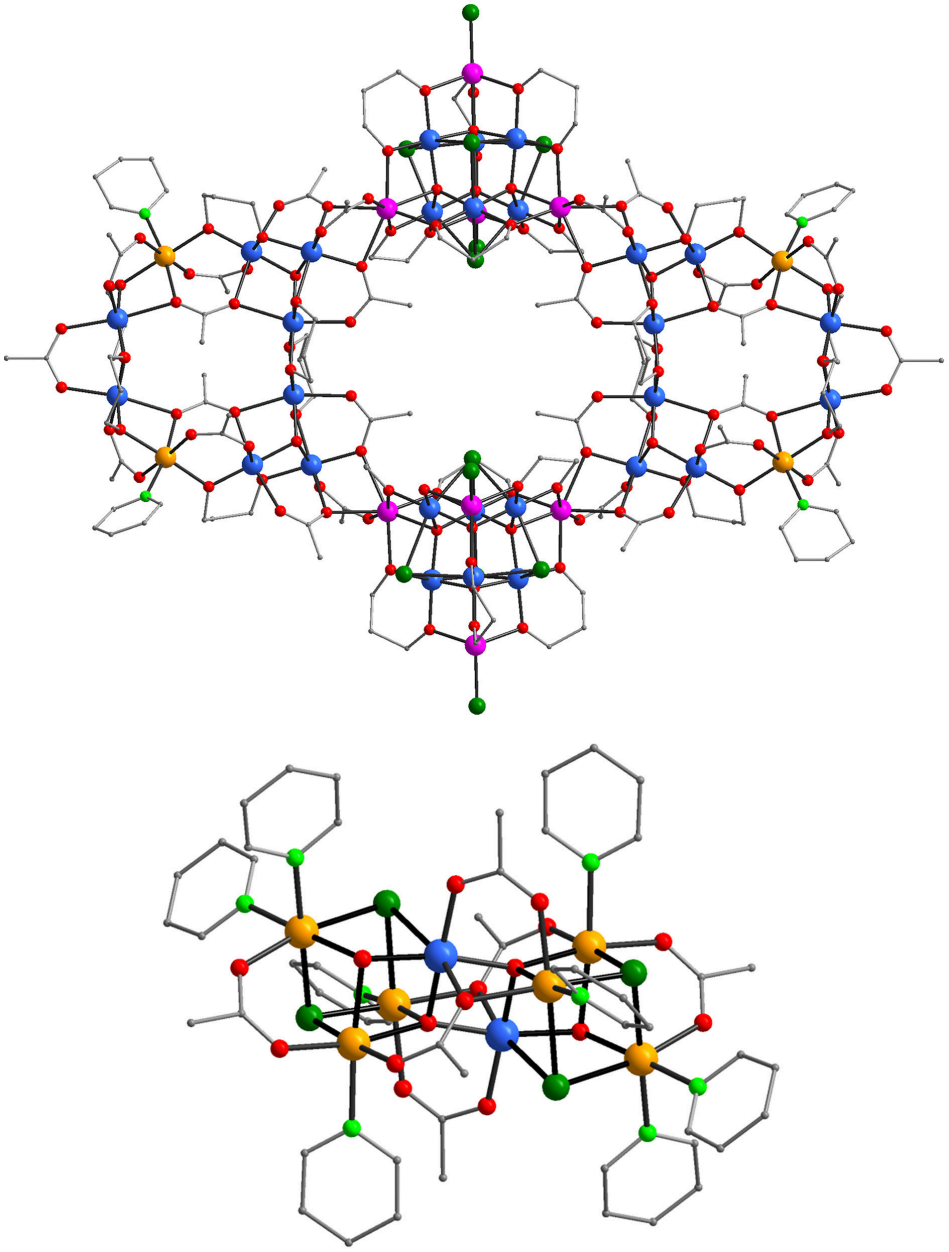
Representations of the molecular structures of the [Mn_36_Ni_4_]^2−^ anion (top) and the [Mn_2_Ni_6_]^2+^ cation (bottom) of **1** Color code: Mn^II^ purple, Mn^III^ blue, Ni^II^ orange, O red, N light green, Cl green, C gray. H atoms are omitted for clarity.

**Figure 2:**
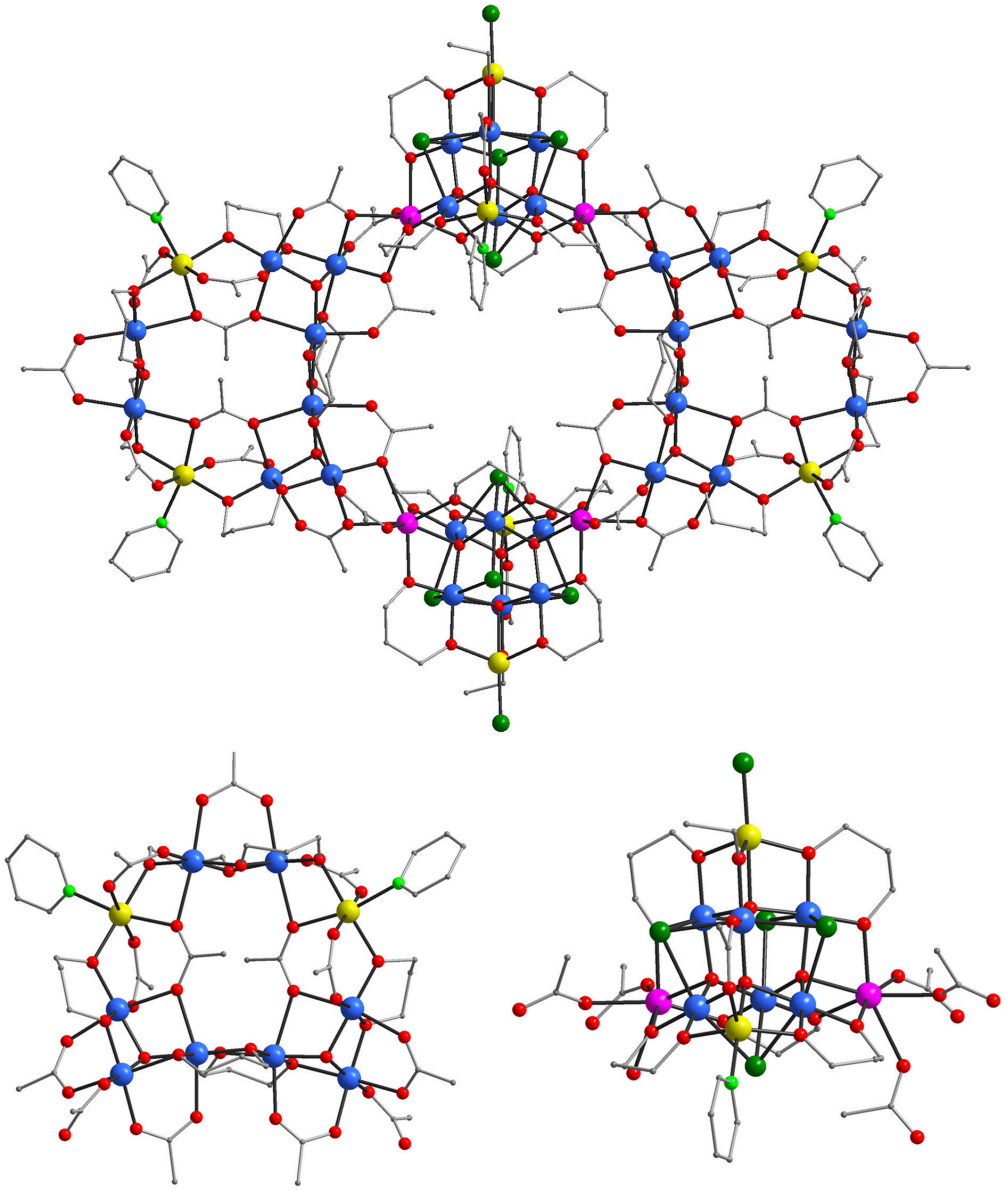
Representations of the molecular structure of **3 (top)** and its [${\text{Mn}}_{8}^{\text{III}}$Co_2_] loop (bottom, **left**) and [${\text{Mn}}_{6}^{\text{III}}$${\text{Mn}}_{2}^{\text{II}}$${\text{Co}}_{2}^{\text{II}}$] supertetrahedral (bottom, **right**) sub-units. Color code: Mn^II^ purple, Mn^III^ blue, Co^II^ yellow, O red; N light green, Cl green, C gray. H atoms are omitted for clarity.

Complexes (**1**) and (**3**)·3.84 EtOH·6H_2_O crystallize in the triclinic space group *P*$\bar{1}$ and (**2**)·2CH_3_CN·12.30H_2_O and (**4**) in the monoclinic *I* 2/a one. The [Mn_36_Ni_4_]^2−^, [Mn_36_Ni_4_], and [Mn_32_Co_8_] aggregates of **1**, **2**, and **3**, respectively exhibit related structures. Similarly, the molecular structures of the [Mn_2_Ni_6_]^2+^ cation of **1** and **4** are also related. Thus, the structures of the [Mn_36_Ni_4_]^2−^ anion and the [Mn_2_Ni_6_]^2+^ cation will be discussed in detail and compared to those of their related analogs **2**–**4**. The molecular structures of the cation and the anion of **1**, and the [Mn_32_Co_8_] aggregate and the sub-units of **3** are shown in Figures 1, 2, respectively. Structural figures and tables for **1**–**4** (bond lengths and angles, Mn/Ni/Co BVS calculations are reported in the Supplementary Material).

The molecular structure of **1** consists of a [Mn_36_Ni_4_]^2−^ anionic aggregate (Figure 1, top) the charge of which is balanced from a cationic [Mn_2_Ni_6_]^2+^ cluster (Figure 1, bottom). Note that the oxidation states of the Mn/Ni ions and the protonation levels of the ligands were determined by bond valence sum calculations (Brown and Altermatt, 1985; Liu and Thorp, 1993), charge considerations and inspection of metric parameters. The giant [Mn_36_Ni_4_]^2−^ aggregate contains two [${\text{Mn}}_{8}^{\text{III}}$Ni_2_(μ_3_-O)_2_(O_2_CCH_3_)_12_(pd)_6_(py)_2_] loops (Figure S1, top) and two [${\text{Mn}}_{6}^{\text{III}}$${\text{Mn}}_{4}^{\text{II}}$(μ_4_-O)_4_(μ_3_-Cl)_4_(O_2_CCH_3_)Cl_2_(pd)_6_(H_2_O)] supertetrahedral sub-units (Figure S1, bottom) which are related to compounds either appeared as fragments in high nuclearity clusters or in a discrete form. In particular the [${\text{Mn}}_{8}^{\text{III}}$Ni_2_] loop exhibits analogous structure to the [${\text{Mn}}_{8}^{\text{III}}$${\text{Mn}}_{2}^{\text{II}}$] sub-unit of the tetrameric [Mn_44_] or [Mn_40_Na_4_] “loops-of-loops” aggregates (vide supra) with their main difference being the presence of two Ni^2+^ ions in the place of two Mn^2+^ ions (Moushi et al., 2010a). In addition, the [${\text{Mn}}_{6}^{\text{III}}$${\text{Mn}}_{4}^{\text{II}}$(μ_4_-O)_4_)]^18+^ supertetrahedral core that is present in **1** has appeared in several compounds either in discrete form (Stamatatos et al., 2006; Manoli et al., 2007, 2008; Wu et al., 2011) or as fragment of higher nuclearity clusters (Manoli et al., 2016). The Mn^III^ and Ni^II^ ions are hexacoordinated exhibiting a distorted octahedral coordination geometry whereas the Mn^II^ ions adopt various coordination numbers and geometries. As expected, the Mn^III^ ions display the expected Jahn-Teller (JT) elongations although the JT axes are not co-parallel.

Thus, each [${\text{Mn}}_{8}^{\text{III}}$Ni_2_] unit consists of two [${\text{Mn}}_{3}^{\text{III}}$(μ_3_-O)]^7+^ triangles and two dinuclear Mn^III^Ni^II^ moieties linked by pd^2−^ RO^−^ groups, and bridging CH_3_${\text{CO}}_{2}^{-}$ ligands. The Mn and Ni ions are held together through 12 acetate and six pd^2−^ bridging ligands. The acetate groups adopt *syn, syn*- η^1^:η^1^:μ (six CH_3_${\text{CO}}_{2}^{-}$ ligands), η^1^:η^2^:μ_3_ (four CH_3_${\text{CO}}_{2}^{-}$ ligands) and η^2^:η^2^:μ_4_ (two CH_3_${\text{CO}}_{2}^{-}$ ligands) bridging modes whereas the pd^2−^ ligands link metal ions in a η^2^:η^2^:μ_3_ fashion. The peripheral ligation of the [${\text{Mn}}_{8}^{\text{III}}$Ni_2_] loop is completed by two terminal py molecules. The [${\text{Mn}}_{8}^{\text{III}}$Ni_2_] loops are connected to the [${\text{Mn}}_{6}^{\text{III}}$${\text{Mn}}_{4}^{\text{II}}$] supertetrahedral sub-units through two μ_3_- and one μ- CH_3_${\text{CO}}_{2}^{-}$ ligands bridging the Mn ions of each [${\text{Mn}}_{3}^{\text{III}}$O]^7+^ triangle to a Mn^II^ ion of a supertetrahedral sub-unit (Figure S3) constructing the nearly—planar [${\text{Mn}}_{28}^{\text{III}}$${\text{Mn}}_{8}^{\text{II}}$Ni_4_] “loop-of-loops-and-supertetrahedra” aggregate. The Mn^II^ ions of the [${\text{Mn}}_{6}^{\text{III}}$${\text{Mn}}_{4}^{\text{II}}$] sub-unit occupy the apex positions of a tetrahedron and the Mn^III^ ions are located on its edges. The metal ions are connected by four μ_4_-O^2−^ ions forming the [${\text{Mn}}_{6}^{\text{III}}$${\text{Mn}}_{4}^{\text{II}}$(μ_4_-O)_4_]^18+^ core which consists of four [${\text{Mn}}_{3}^{\text{III}}$Mn^II^(μ_4_-O)]^9+^ vertex-sharing tetrahedra. The Mn^III^ ions are bridged through four μ_3_- Cl^−^ ions which occupy their JT axes. The two Mn^2+^ and one Mn^3+^ ions located in each edge of the tetrahedron are connected through six pd^2−^ ligands bridging in a η^2^:η^2^:μ_3_ mode. The peripheral ligation of the [${\text{Mn}}_{6}^{\text{III}}$${\text{Mn}}_{4}^{\text{II}}$(μ_4_-O)_4_(μ_3_-Cl)_4_(pd)_6_(O_2_CCH_3_)Cl_2_] subunit (Figure S1) is completed by two terminal Cl^−^ anions.

The molecular structure of the [${\text{Mn}}_{2}^{\text{III}}$${\text{Ni}}_{6}^{\text{II}}$] cation consists of two mixed metal [Mn^III^${\text{Ni}}_{3}^{\text{II}}$(μ_4_-O)(μ_3_-OH)_1.5_(μ_3_-Cl)_1.5_(O_2_CMe)_3_(py)_4_]^+^ cubanes linked through oxide and carboxylate ligands. In particular, the three Ni^II^ and one Mn^III^ ions are connected through one μ_4_-O^2−^, one μ_3_-OH^−^, one μ_3_-Cl^−^ and a mixed 0.5 Cl^−^/0.5 OH^−^ site. The μ_3_-OH^−^ and μ_3_-Cl^−^ bridge two 2 Ni^II^ and one Mn^III^ ions, the mixed 0.5 Cl^−^/0.5 OH^−^ connects 3 Ni^II^ ions of the cubane whereas the μ_4_-O^2−^ links 2 Ni^II^ and one Mn^III^ ions of the cubane thus forming the [Mn^III^${\text{Ni}}_{3}^{\text{II}}$(μ_4_-O)(μ_3_-OH)_1.5_(μ_3_-Cl)_1.5_]^4+^ cubane core and one additional Mn^III^ ion of the second cubane of the cation of **1**. The Mn/Ni ions of each cubane are also bridged by three carboxylate ligands two of which bridge in the common *syn, syn*- η^1^:η^1^:μ fashion and the third one in a η^1^:η^2^:μ_3_ one. Their peripheral ligation is completed by four terminal pyridine molecules linked to the Ni^II^ ions. The two cubanes of the cation of **1** are linked apart from the μ_4_-O^2−^ ions from two *syn, syn*- η^1^:η^1^:μ and two η^1^:η^2^:μ_3_ carboxylate ligands constructing the [Mn_2_Ni_6_(μ_4_-O)_2_(μ_3_-OH)_3_(μ_3_-Cl)_3_(O_2_CCH_3_)_6_(py)_8_]^2+^ cation of **1**.

The molecular structure of the [Mn_36_Ni_4_] cluster of **2**·2CH_3_CN·12.30H_2_O exhibits a striking similarity to the anion of **1**. The main difference between the two complexes is their overall charge since compound **1** is anionic with a 2- charge and **2** is neutral. This difference in the charges appears because a terminal Cl^−^ ligand of **1** linked to a Mn^II^ ion of the supertetrahedral sub-units has been replaced in **2** by a terminal H_2_O molecule. As a result, compound **2** contains two less Cl^−^ anions and is neutral and thus, the [Mn_36_Ni_4_] aggregate is the only metal cluster appearing in the crystal structure.

The molecular structure of (**3)**·3.84 EtOH·6H_2_O (Figure 2, top) is also related to the anion of **1** and to **2**·2CH_3_CN·12.30H_2_O with the main difference obviously being the presence in **3** of 32 Mn/8 Co ions instead of 36 Mn/4 Ni ions that appear in the anion of **1** and in **2**. These eight Co^II^ ions are located in the decametallic [${\text{Mn}}_{8}^{\text{III}}$${\text{Co}}_{2}^{\text{II}}$] loops (Figure 2, bottom left) in the same positions that the Ni^II^ ions of the [Mn_36_Ni_4_] aggregates are found and also in the [${\text{Mn}}_{6}^{\text{III}}$${\text{Mn}}_{2}^{\text{II}}$${\text{Co}}_{2}^{\text{II}}$] supertetrahedral sub-units (Figure 2, bottom right). In the latter the Co^II^ ions occupy two apex positions in which Mn^II^ ions are located in the known [${\text{Mn}}_{6}^{\text{III}}$${\text{Mn}}_{4}^{\text{II}}$] supertetrahedra including the sub-units of the anion of **1** and in **2**. In fact, this is a major difference between the [Mn_36_Ni_4_]^0/2−^ and [Mn_32_Co_8_] since the former ones consist of one heterometallic [${\text{Mn}}_{8}^{\text{III}}$${\text{Ni}}_{2}^{\text{II}}$] and one homometallic [${\text{Mn}}_{6}^{\text{III}}$${\text{Mn}}_{4}^{\text{II}}$] sub-units, whereas the latter is based on two different types of heterometallic sub-units. Apart from these major differences in the structures of the [Mn_36_Ni_4_]^0/2−^ and [Mn_32_Co_8_] compounds there are also some minor ones. These include the presence in the structure of [Mn_32_Co_8_] aggregate of two less pd^2−^ ligands and two additional CH_3_${\text{CO}}_{2}^{-}$ and C_2_H_5_O^−^ groups compared to the structures of the [Mn_36_Ni_4_]^0/2−^ complexes. This is because one pd^2−^ group bridging 2 Mn^II^/1Mn^III^ ions of one edge of each supertetrahedron in [Mn_36_Ni_4_]^0/2−^ complexes has been replaced by a *syn, syn*- η^1^:η^1^:μ- CH_3_${\text{CO}}_{2}^{-}$ and a μ-EtO ligands in [Mn_32_Co_8_] aggregate. In addition, [Mn_32_Co_8_] aggregate contains a terminal py ligand bound to a Co^II^ ion located in the [${\text{Mn}}_{6}^{\text{III}}$${\text{Mn}}_{2}^{\text{II}}$${\text{Co}}_{2}^{\text{II}}$] supertetrahedra in the place of a Cl^−^ ligand or a H_2_O molecule in the anion of **1** or **2**, respectively.

The molecular structure of compound **4** is related to that of the cation of **1**. In fact, there are only very minor differences between the two compounds. These include the replacement in **4** of the mixed 0.5 Cl^−^/0.5 OH^−^ site that bridges 3 Ni^II^ ions of each cubane by a μ_3_-OH^−^ anion. In addition, in complex **4** the positive charge of the [${\text{Mn}}_{2}^{\text{III}}$${\text{Ni}}_{6}^{\text{II}}$]^2+^ cation is balanced by a Cl${\text{O}}_{4}^{-}$ and a OH^−^ lattice anions instead of the [Mn_36_Ni_4_]^2−^ anionic aggregate.

### Magnetic Properties

**Figure 3:**
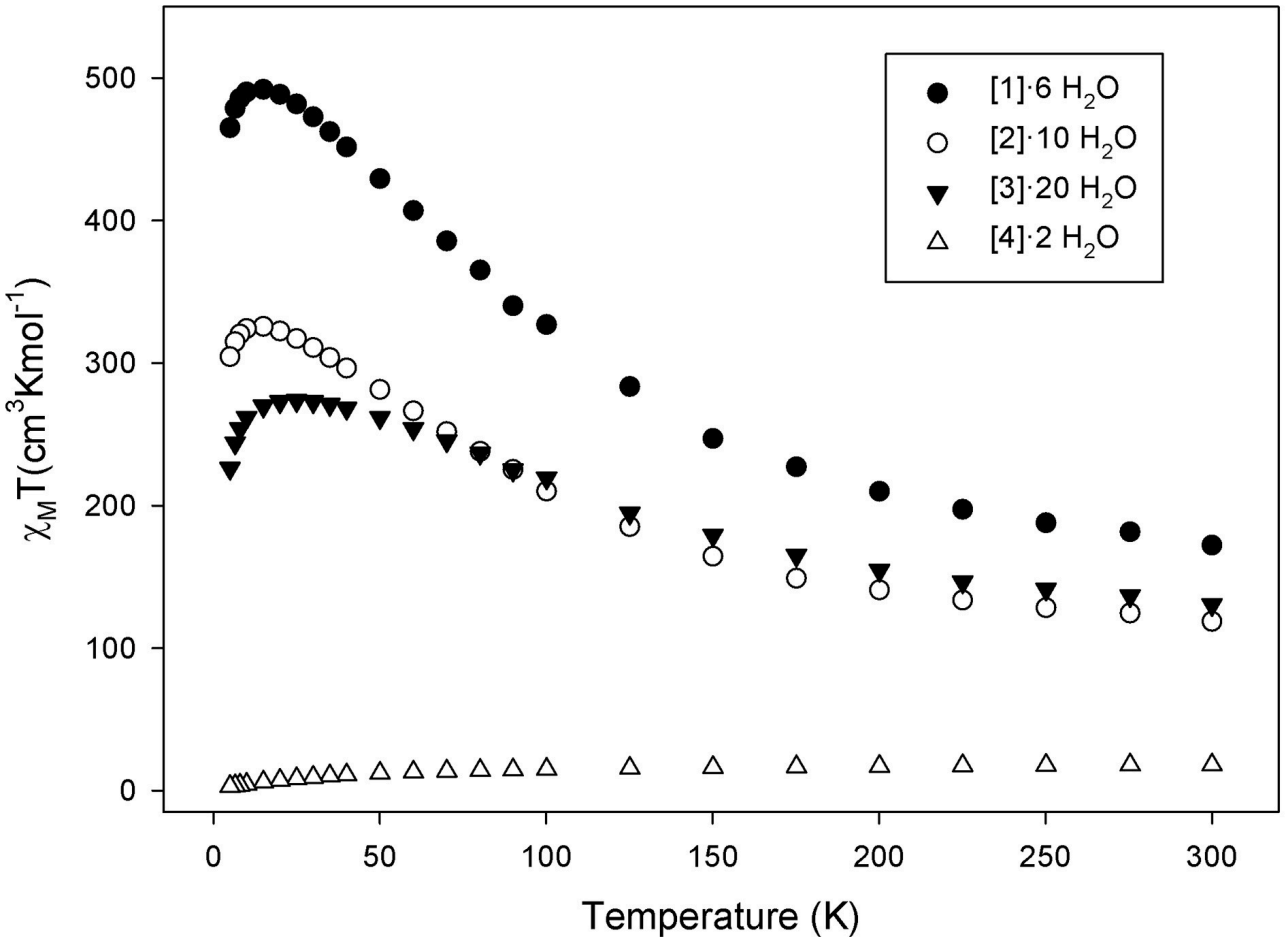
χ_M_T vs. T plots for complexes (**1**)·6 H_2_O (•), (**2**)·10 H_2_O (◦), (**3**)·20 H_2_O (▾), and (**4**)·2 H_2_O (Δ) in the temperature range 5–300 K in a 0.1 T applied dc field.

Solid-state dc magnetic susceptibility measurements were performed on polycrystalline samples of complexes **1**·6 H_2_O, **2**·10 H_2_O, **3**·20 H_2_O, and **4**·2 H_2_O under a magnetic field of 0.1 T in the temperature range 5-300 K. The obtained data are shown as χ_M_*T* vs. *T* plot in Figure 3. For complexes **1**·6 H_2_O, **2**·10 H_2_O, and **3**·20 H_2_O the χ_M_*T* value increases continuously from 172.1, 118.6, and 130.5 cm^3^ mol^−1^ K at 300 K to a maximum value of 492.0 (at 20 K), 325.6 (at 15 K), and 273.7 cm^3^ mol^−1^ K (at 25 K) and then decreases at low T to 465.1, 304.3, and 226.1 cm^3^ mol^−1^ K at 5 K, respectively. For **4**·2 H_2_O, the χ_M_*T* value at 300 K is 18.03 cm^3^ mol^−1^ K and decreases continuously with decreasing temperature reaching a value of ~15.03 cm^3^ mol^−1^ K at 100 K and then rapidly to 2.94 cm^3^ mol^−1^ K at 5.0K (Figure 3 and Figure S6). The increase of the χ_M_*T* values with decreasing T in **1**·6 H_2_O—**3**·20 H_2_O indicates the presence of dominant ferromagnetic exchange interactions. The maximum χ_M_*T* values of **2**·10 H_2_O and **3**·20 H_2_O suggest S_T_ values of ~ 26 ± 1 and 22 ± 1, respectively. However, we note that in the case of **3**·20 H_2_O it may not be safe to exclude any conclusions for the spin ground state using the spin-only formula due to the presence of Co^II^ ions which is well known that exhibit strong spin-orbit coupling. The decrease in the χ_M_*T* value at the lowest temperatures is attributed to zero-field splitting (ZFS), Zeeman effects from the applied field, and/or any weak intermolecular antiferromagnetic exchange interactions. In the case of **4**·2 H_2_O, the continuous decrease of χ_M_*T* with decreasing temperature, the small χ_M_*T* value at 5 K and the fact that the curve heads to 0 at 0 K suggests the presence of antiferromagnetic exchange interactions possibly leading to a diamagnetic ground state. This may be rationalized assuming that the two [Mn^III^${\text{Ni}}_{3}^{\text{II}}$] are antiferromagnetically coupled leading to a diamagnetic ground state.

Given the size and the complexity of the structures of **1**–**3**, it is not possible to apply the Kambe method to determine the individual pairwise Mn-Mn, Mn-Ni, and Mn-Co exchange interaction parameters. In addition, the existence of two complexes co-crystallizing together in the structure of **1**·6 H_2_O does not allow to obtain information for the D and S_T_ values of these complexes. Furthermore, in **3**·20 H_2_O, the presence of Co^II^ ions exhibiting strong spin-orbit coupling and of many low lying excited states appearing due to the complexity of the giant [Mn_32_Co_8_] aggregate did not allow to obtain reliable S and D values from reduced magnetization fitting.

In the case of compound **2**·10 H_2_O magnetization vs. dc field measurements at applied magnetic fields and temperatures in the 1–10 kG and 1.8–4.0 K ranges, respectively were performed. Low field data (≤ 1.0 T) were used, as we have previously done for many Mn clusters containing Mn^II^ atoms, to avoid problems from low-lying excited states. The data for complex **2**·10 H_2_O are shown in Figure S7 as reduced magnetization (M/Nμ_B_) vs. H/T plot, where M is the magnetization, N is Avogadro's number, μ_B_ is the Bohr magneton, and H is the magnetic field.

The *M*/*N*μ_B_ vs. *H*/*T* data were fit by assuming that only the ground state is populated and by including axial zero-field splitting (*D*${Ŝ}_{\text{z}}^{2}$) and isotropic Zeeman interactions. The corresponding spin Hamiltonian is given by Equation (5),

(5) $$\begin{array}{r} \text{H}=\text{D}{Ŝ}_{\text{z}}^{2}+\text{g}\mu_{\text{B}}\mu_{0}Ŝ\cdot \text{H} \end{array}$$

where *D* is the axial ZFS parameter, Ŝ_z_ is the easy-axis spin operator, μ_0_ is the vacuum permeability, and *H* is the applied field. Equal quality fits, shown as the solid lines in Figure S7, were obtained for *S* = 25, 26, and 27 with parameters *g* = 2.03(1)/*D* = −0.007(1) cm^−1^, *g* = 1.96(1)/*D* = −0.004(1) cm^−1^, and *g* = 1.91(1)/*D* = −0.004(1) cm^−1^, respectively. Based on the obtained fits, we conclude that **2**·10 H_2_O has a ground state of *S*_*T*_ = 26 ± 1, and a very small D value.

Alternating current (ac) magnetic susceptibility data were collected for compounds **1**–**3** to obtain additional information about their S_T_ values and the possibility to exhibit slow relaxation of the magnetization phenomena indicative of SMM behavior. The temperature dependence of the in-phase ($\chi_{\text{M}}^{\prime }$), shown as $\chi_{\text{M}}^{\prime }$T and out-of-phase ($\chi_{\text{M}}^{\prime \prime }$) ac signals for **1**–**3** is shown in Figures S8–S10). These studies revealed that there are not any out-of-phase ac signals in **1** and **2** suggesting that these compounds are not new SMMs. In the case of **3**, there is a barely visible beginning in the 1.8 K data of a frequency dependence in the in-phase plot and a concomitant very weak tail of an out-of-phase signal ($\chi_{\text{M}}^{\prime \prime }$/$\chi_{\text{M}}^{\prime }$ ~ 1.5% at 1.8 K) representing the beginning of a $\chi_{\text{M}}^{\prime \prime }$ signal whose peak is clearly far below the 1.8 K limit of our SQUID instrument, i.e., the anisotropy barrier is extremely small. In addition, ac data are in line with the conclusions obtained from dc studies concerning the S_T_ values of **2** and **3**. In particular, extrapolation of the χ_M_'*T* signal of compounds **2** and **3** to 0 K from above ~8 K (to avoid the effects of intermolecular interactions at lower temperatures) gives values of ~340 and 255 cm^3^mol^−1^ K, respectively. These values are consistent with S_T_ in the range 25–27 (*S*_T_ = 25, *g* = 2.05; *S*_T_ = 26, *g* = 1.97; *S*_T_ = 27, *g* = 1.90) for **2** and 21–23 (*S*_T_ = 21, *g* = 2.10; *S*_T_ = 22, *g* = 2.01; *S*_T_ = 23, *g* = 1.92) for **3**.

## Discussion

We described herein the synthesis, crystal structures and magnetic properties of a series of high nuclearity mixed metal Mn/Ni and Mn/Co clusters. The first member of this family of complexes, compound **1**, was prepared by targeted modifications in the reaction procedures that afforded the family of [Mn_44_] and [Mn_40_Na_4_] “loops-of-loops” aggregates reported by our group previously (Moushi et al., 2010a). Since complex **1** consists of a [Mn_36_Ni_4_]^2−^ anion which co-crystallized together with a [Mn_2_Ni_6_]^2+^ cation, we targeted and achieved, following synthetic procedures containing elements of rational design, the isolation of the discrete [Mn_36_Ni_4_] (compound **2**) and [Mn_2_Ni_6_]^2+^ (the cation of **4**) clusters and also of another Mn/3d analog the [Mn_32_Co_8_] aggregate of **3**. Arguably most of the known giant molecular aggregates have been afforded from serendipitous assembly synthetic procedures although recently there have been a few reports about elegant, rationally designed synthetic strategies that led to such high nuclearity clusters (Papatriantafyllopoulou et al., 2016 and references therein). The isolation of **2**–**4** thus, represents a rare example of targeted synthesis of high nuclearity metal—organic complexes from procedures that contain elements of rational design.

The heterometallic [Mn_36_Ni_4_]^2−/0^ and [Mn_32_Co_8_] “loops-of-loops-and-supertetrahedra” molecular aggregates are giant clusters exhibiting nanosized dimensions and large molecular weights approaching those of small proteins (for example the MW of compound **1** is ~8,095 g/mol and is comparable to those of small proteins which are ~10 kDa). In fact these are the second highest nuclearity heterometallic Mn_x_M_y_ (M = any metal ion) clusters and M_x_M'_y_ (M, M′ = any 3d metal ion) with the highest nuclearity heterometallic Mn-containing complex and mixed 3d metal/3d metal cluster being a [Mn_28_Cu_17_] aggregate (Wang et al., 2007). Interestingly, the [Mn_36_Ni_4_]^2−/0^ and [Mn_32_Co_8_] aggregates are based on polynuclear sub-units that have appeared either as fragments and/or in a discrete form previously (Stamatatos et al., 2006; Manoli et al., 2007, 2008, 2016; Moushi et al., 2010a; Wu et al., 2011). This structural feature makes them a member of a very small family of large matal-organic clusters based on known in a discrete form polynuclear repeating units (Manoli et al., 2016; Papatriantafyllopoulou et al., 2016). Their [${\text{Mn}}_{6}^{\text{III}}$${\text{Mn}}_{4}^{\text{II}}$(μ_4_-O)_4_]^18+^ supertetrahedral repeating unit is very well-known in Mn cluster chemistry and has attracted significant interest due to its symmetric structure and the fact that most of the compounds containing this core exhibit entirely ferromagnetic exchange interactions and the maximum possible, for a [${\text{Mn}}_{6}^{\text{III}}$${\text{Mn}}_{4}^{\text{II}}$] complex, S_T_ = 22 spin ground state value. The presence of this repeating unit in the [Mn_36_Ni_4_]^2−/0^ and [Mn_32_Co_8_] “loops-of-loops-and-supertetrahedra” aggregates ensures the appearance of dominant ferromagnetic exchange interactions between their metal ions and of a large S_T_ value. Notably there are no Ni^II^ ions located in this sub-unit in the [Mn_36_Ni_4_]^2−/0^ clusters since all of them are found in the decametallic loops. However, in the [Mn_32_Co_8_] aggregate there are two Co^II^ ions in each decametallic supertetrahedron located in the apex positions replacing two Mn^II^ ions. In fact, there have been reported some heterometallic Mn_10−x_M_x_ (M = any metal ion) supertetrahedra where the Mn^II^ ions are partially or completely replaced, however, to the best of our knowledge there are no mixed metal Mn_10−x_Co_x_ analogs in the literature. Such compounds could be very attractive magnetically since they could possibly combine the ferromagnetic exchange interactions and large S_T_ values appearing in the decametallic Mn-based supertetrahedra with a significant anisotropy due to the presence of Co^II^ ions. The appearance of the Co^II^ ions in the apex positions of the supertetrahedra is not surprising, not only because they replace a metal ion being in the same oxidation state (Mn^II^) and as a result there are no charge variations in the new compound but also these positions have proven to be the most labile in this family of [${\text{Mn}}_{6}^{\text{III}}$${\text{Mn}}_{4}^{\text{II}}$] complexes. This is also supported from the isolation a series of heterometallic [[${\text{Mn}}_{6}^{\text{III}}$${\text{Mn}}_{\text{x}}^{\text{II}}$M_4−x_] (M = any metal ion) supertetrahedra, appearing in a discrete form and also as fragments of high nuclearity clusters, in which the Mn^II^ ions have been completely or partially replaced by other metal ions (Skordi et al., 2018 and references therein). The [Mn_2_Ni_6_]^2+^ cation appears also for the first time in heterometallic cluster chemistry although there are homometallic [${\text{Mn}}_{6}^{\text{II}}$${\text{Mn}}_{2}^{\text{III}}$] clusters reported exhibiting an analogous structural core (Boskovic et al., 2002).

Magnetism studies revealed that the [Mn_36_Ni_4_]^2−/0^/[Mn_32_Co_8_] aggregates exhibit dominant ferromamagnetic exchange interactions and large spin ground state values *S*_*T*_ = 26 ± 1 ([Mn_36_Ni_4_]) and 22 ± 1 ([Mn_32_Co_8_]), although in the latter it is not safe to conclude for the S_T_ value due to the presence of the anisotropic Co^II^ ions. On the other hand, the [Mn_2_Ni_6_]^2+^ cation of **4** exhibits dominant antiferromagnetic exchange interactions leading to a diamagnetic S_T_ value. In fact, a diamagnetic ground state has also been reported for the analogous homometallic [${\text{Mn}}_{6}^{\text{II}}$${\text{Mn}}_{2}^{\text{III}}$] cluster (Boskovic et al., 2002). This behavior could result from the presence of antiferromagnetic exchange interactions between the two [Mn^III^${\text{Ni}}_{3}^{\text{II}}$] (in **4**) or [Mn^III^${\text{Mn}}_{3}^{\text{II}}$] (in the [${\text{Mn}}_{6}^{\text{II}}$${\text{Mn}}_{2}^{\text{III}}$]) cubane sub-units leading to diamagnetic ground states. The reported S_T_ values for **2** and **3** are among the larger ones for heterometallic aggregates with the S_T_ ≈ 26 being the second highest value reported for a heterometallic cluster (Chen et al., 2018). Clearly the overall magnetic behavior of **2** exhibits remarkable analogies with that of its [${\text{Mn}}_{6}^{\text{III}}$${\text{Mn}}_{4}^{\text{II}}$] building block since they both display ferromagnetic exchange interactions, large S_T_ and small D values and are not SMMs. On the other hand, in the case of **3** although it is not safe to conclude about the S_T_ value, however, it is clear that the spin ground state value in **3** is smaller than that of **2**. This can be attributed to the existence of more heterometal ions, since in **3** there are 8 Co^II^ ions whereas in **2** only 4 Ni^II^ ions and also to the presence of stronger antiferromagnetic exchange interactions as expected for Mn/Co and Mn/Ni heterometallic compounds. In addition, the out-of-phase ac signals at low T in **3** appear due to the presence of the anisotropic Co^II^ ions leading to the increase of the anisotropy and possibly to SMM behavior.

Summarizing, a series of heterometallic [Mn_36_Ni_4_]^2−/0^ and [Mn_32_Co_8_] “loops-of-loops-and-supertetrahedra” molecular aggregates and the cationic [${\text{Mn}}_{2}^{\text{III}}$${\text{Ni}}_{6}^{\text{II}}$]^2+^ cluster were prepared by employing synthetic procedures containing elements of rational design. The “loops-of-loops-and-supertetrahedra” molecular aggregates of **1**–**3** are among the largest heterometallic Mn-containing clusters exhibiting dimensions and molecular weights comparable to those of small proteins. In addition, they exhibit dominant ferromagnetic exchange interactions and very large S_T_ values. These compounds are new additions in the very small family of giant Mn_x_M_y_ (M = any metal ion) aggregates. Since this area is merely unexplored, further studies targeting to “loops-of-loops-and-supertetrahedra” analogs with various 3d and 4f metal ions and other high nuclearity heterometallic Mn/M clusters are in progress and the results will be reported in due course.

## Author Contributions

MC was involved on the synthesis, crystallization, and characterization of the reported complexes. EM was involved on the synthesis, crystallization, and characterization of the reported complexes and on manuscript preparation. TN was involved on the investigation of the magnetic properties of compounds **3** and **4**. CP was involved on the investigation of the magnetic properties of compounds **1** and **2** and on manuscript preparation. VN was involved on the refinement of the crystal structures of **1**–**4** and on manuscript preparation. GC was involved on the investigation of the magnetic properties of compounds **1**–**2** and on manuscript preparation. AT supervised the reported work and was involved on all parts of the project.

### Conflict of Interest Statement

The authors declare that the research was conducted in the absence of any commercial or financial relationships that could be construed as a potential conflict of interest. The handling editor declared a past co-authorship with several of the authors (CP, VN, GC, AT).

## Footnotes

^1^MAGNET, Indiana University.
