# Supplementary material for: Giant Heterometallic [Mn36Ni4]0/2− and [Mn32Co8] “Loops-of-Loops-and-Supertetrahedra” Molecular Aggregates
Source: Front Chem. 2019 Mar 5;7:96. doi: 10.3389/fchem.2019.00096 (PMC6413240; doi:10.3389/fchem.2019.00096)

checkCIF/PLATON report

You have not supplied any structure factors. As a result the full set of tests cannot be run.

THIS REPORT IS FOR GUIDANCE ONLY. IF USED AS PART OF A REVIEW PROCEDURE FOR PUBLICATION, IT SHOULD NOT REPLACE THE EXPERTISE OF AN EXPERIENCED CRYSTALLOGRAPHIC REFEREE.

No syntax errors found. CIF dictionary Interpreting this report

Datablock: mc221_Final

```
Bond precision:  C-C = 0.0101 A          Wavelength=0.71073

Cell:          a=19.2325(3)      b=21.5359(5)     c=26.0165(6)
               alpha=85.877(3)   beta=71.202(2)   gamma=67.749(2)
Temperature:   100 K
```

	Calculated	Reported
Volume	9425.9(4)	9425.9(4)
Space group	P -1	P -1
Hall group	-P 1	-P 1
Moiety formula	C144 H242 Cl12 Mn36 N4 Ni4 O112, C52 H58 Cl3 Mn2 N8 Ni6 O17 [+	C144 H242 Cl12 Mn36 N4 Ni4 O112, C52 H58 Cl3 Mn2 N8 Ni6 O17
Sum formula	C196 H300 Cl15 Mn38 N12 Ni10 O129 [+ solvent]	C196 H300 Cl15 Mn38 N12 Ni10 O129
Mr	8094.86	8094.86
Dx,g cm-3	1.426	1.426
Z	1	1
Mu (mm-1)	1.883	1.883
F000	4077.0	4077.0
F000’	4097.00	
h,k,lmax	23,26,32	23,26,32
Nref	37040	36964
Tmin,Tmax	0.763,0.860	0.918,1.000
Tmin’	0.726	

```
Correction method= # Reported T Limits: Tmin=0.918 Tmax=1.000
AbsCorr = MULTI-SCAN

Data completeness= 0.998          Theta(max)= 26.000

R(reflections)= 0.0585( 21597)    wR2(reflections)= 0.1699( 36964)

S = 1.072                   Npar= 1819
```

The following ALERTS were generated. Each ALERT has the format
test-name_ALERT_alert-type_alert-level.
Click on the hyperlinks for more details of the test.

Alert level C

```
PLAT220_ALERT_2_C Non-Solvent  Resd 1  C   Ueq(max)/Ueq(min) Range        4.8 Ratio
PLAT222_ALERT_3_C Non-Solv.  Resd 1  H   Uiso(max)/Uiso(min) Range        4.2 Ratio
PLAT241_ALERT_2_C High   'MainMol' Ueq as Compared to Neighbors of        O58 Check
PLAT241_ALERT_2_C High   'MainMol' Ueq as Compared to Neighbors of        C21 Check
PLAT241_ALERT_2_C High   'MainMol' Ueq as Compared to Neighbors of        C23 Check
PLAT241_ALERT_2_C High   'MainMol' Ueq as Compared to Neighbors of        C26 Check
PLAT241_ALERT_2_C High   'MainMol' Ueq as Compared to Neighbors of        C71 Check
PLAT242_ALERT_2_C Low    'MainMol' Ueq as Compared to Neighbors of        C69 Check
PLAT250_ALERT_2_C Large U3/U1 Ratio for Average U(i,j) Tensor ....        2.1 Note
PLAT341_ALERT_3_C Low Bond Precision on  C-C Bonds ...............    0.01015 Ang.
PLAT413_ALERT_2_C Short Inter XH3 .. XHn     H19A     ..H66A            2.13 Ang.
                                                     1+x,y,z  =      1_655 Check
```

Alert level G

```
PLAT002_ALERT_2_G Number of Distance or Angle Restraints on AtSite          9 Note
PLAT003_ALERT_2_G Number of Uiso or Uij Restrained non-H Atoms ...          9 Report
PLAT172_ALERT_4_G The CIF-Embedded .res File Contains DFIX Records          3 Report
PLAT178_ALERT_4_G The CIF-Embedded .res File Contains SIMU Records          1 Report
PLAT186_ALERT_4_G The CIF-Embedded .res File Contains ISOR Records          2 Report
PLAT187_ALERT_4_G The CIF-Embedded .res File Contains RIGU Records          1 Report
PLAT300_ALERT_4_G Atom Site Occupancy of C20A       Constrained at        0.6 Check
PLAT300_ALERT_4_G Atom Site Occupancy of C20B       Constrained at        0.4 Check
PLAT300_ALERT_4_G Atom Site Occupancy of H19A       Constrained at        0.6 Check
PLAT300_ALERT_4_G Atom Site Occupancy of H19B       Constrained at        0.6 Check
PLAT300_ALERT_4_G Atom Site Occupancy of H20A       Constrained at        0.6 Check
PLAT300_ALERT_4_G Atom Site Occupancy of H20B       Constrained at        0.6 Check
PLAT300_ALERT_4_G Atom Site Occupancy of H21A       Constrained at        0.6 Check
PLAT300_ALERT_4_G Atom Site Occupancy of H21B       Constrained at        0.6 Check
PLAT300_ALERT_4_G Atom Site Occupancy of H19C       Constrained at        0.4 Check
PLAT300_ALERT_4_G Atom Site Occupancy of H19D       Constrained at        0.4 Check
PLAT300_ALERT_4_G Atom Site Occupancy of H20C       Constrained at        0.4 Check
PLAT300_ALERT_4_G Atom Site Occupancy of H20D       Constrained at        0.4 Check
PLAT300_ALERT_4_G Atom Site Occupancy of H21C       Constrained at        0.4 Check
PLAT300_ALERT_4_G Atom Site Occupancy of H21D       Constrained at        0.4 Check
PLAT300_ALERT_4_G Atom Site Occupancy of Cl8        Constrained at        0.5 Check
PLAT300_ALERT_4_G Atom Site Occupancy of O9         Constrained at        0.5 Check
PLAT301_ALERT_3_G Main Residue  Disorder ..............(Resd  1  )         1% Note
PLAT302_ALERT_4_G Anion/Solvent/Minor-Residue Disorder (Resd  2  )         2% Note
PLAT380_ALERT_4_G Incorrectly? Oriented X(sp2)-Methyl Moiety .....        C38 Check
PLAT380_ALERT_4_G Incorrectly? Oriented X(sp2)-Methyl Moiety .....        C40 Check
PLAT380_ALERT_4_G Incorrectly? Oriented X(sp2)-Methyl Moiety .....        C42 Check
PLAT380_ALERT_4_G Incorrectly? Oriented X(sp2)-Methyl Moiety .....        C44 Check
PLAT380_ALERT_4_G Incorrectly? Oriented X(sp2)-Methyl Moiety .....        C46 Check
PLAT380_ALERT_4_G Incorrectly? Oriented X(sp2)-Methyl Moiety .....        C48 Check
PLAT380_ALERT_4_G Incorrectly? Oriented X(sp2)-Methyl Moiety .....        C50 Check
PLAT380_ALERT_4_G Incorrectly? Oriented X(sp2)-Methyl Moiety .....        C52 Check
PLAT380_ALERT_4_G Incorrectly? Oriented X(sp2)-Methyl Moiety .....        C54 Check
PLAT380_ALERT_4_G Incorrectly? Oriented X(sp2)-Methyl Moiety .....        C56 Check
PLAT380_ALERT_4_G Incorrectly? Oriented X(sp2)-Methyl Moiety .....        C58 Check
PLAT380_ALERT_4_G Incorrectly? Oriented X(sp2)-Methyl Moiety .....        C60 Check
PLAT380_ALERT_4_G Incorrectly? Oriented X(sp2)-Methyl Moiety .....        C62 Check
PLAT380_ALERT_4_G Incorrectly? Oriented X(sp2)-Methyl Moiety .....        C64 Check
PLAT380_ALERT_4_G Incorrectly? Oriented X(sp2)-Methyl Moiety .....        C66 Check
```

```
PLAT380_ALERT_4_G Incorrectly? Oriented X(sp2)-Methyl Moiety .....        C68 Check
PLAT606_ALERT_4_G VERY LARGE Solvent Accessible VOID(S) in Structure        ! Info
PLAT764_ALERT_4_G Overcomplete CIF Bond List Detected (Rep/Expd) .       1.12 Ratio
PLAT794_ALERT_5_G Tentative Bond Valency for Ni1       (II)      .       2.02 Info
PLAT794_ALERT_5_G Tentative Bond Valency for Ni2       (II)      .       2.01 Info
PLAT794_ALERT_5_G Tentative Bond Valency for Mn1       (I)       .       0.92 Info
PLAT794_ALERT_5_G Tentative Bond Valency for Mn2       (I)       .       0.92 Info
PLAT794_ALERT_5_G Tentative Bond Valency for Mn3       (I)       .       0.90 Info
PLAT794_ALERT_5_G Tentative Bond Valency for Mn4       (I)       .       0.90 Info
PLAT794_ALERT_5_G Tentative Bond Valency for Mn5       (I)       .       0.90 Info
PLAT794_ALERT_5_G Tentative Bond Valency for Mn7       (I)       .       0.92 Info
PLAT794_ALERT_5_G Tentative Bond Valency for Mn8       (I)       .       0.91 Info
PLAT794_ALERT_5_G Tentative Bond Valency for Mn10      (III)     .       3.20 Info
PLAT794_ALERT_5_G Tentative Bond Valency for Mn11      (II)      .       2.11 Info
PLAT794_ALERT_5_G Tentative Bond Valency for Mn12      (I)       .       0.80 Info
PLAT794_ALERT_5_G Tentative Bond Valency for Mn13      (II)      .       1.92 Info
PLAT794_ALERT_5_G Tentative Bond Valency for Mn14      (III)     .       3.15 Info
PLAT794_ALERT_5_G Tentative Bond Valency for Mn16      (III)     .       3.16 Info
PLAT794_ALERT_5_G Tentative Bond Valency for Mn18      (I)       .       0.78 Info
PLAT860_ALERT_3_G Number of Least-Squares Restraints .............         93 Note
PLAT869_ALERT_4_G ALERTS Related to the Use of SQUEEZE Suppressed           ! Info
PLAT933_ALERT_2_G Number of OMIT Records in Embedded .res File ...          7 Note
```

---

  0 **ALERT level A** = Most likely a serious problem - resolve or explain
  0 **ALERT level B** = A potentially serious problem, consider carefully
 11 **ALERT level C** = Check. Ensure it is not caused by an omission or oversight
 61 **ALERT level G** = General information/check it is not something unexpected

```
  0 ALERT type 1 CIF construction/syntax error, inconsistent or missing data
 12 ALERT type 2 Indicator that the structure model may be wrong or deficient
  4 ALERT type 3 Indicator that the structure quality may be low
 40 ALERT type 4 Improvement, methodology, query or suggestion
 16 ALERT type 5 Informative message, check
```

It is advisable to attempt to resolve as many as possible of the alerts in all categories. Often the minor alerts point to easily fixed oversights, errors and omissions in your CIF or refinement strategy, so attention to these fine details can be worthwhile. In order to resolve some of the more serious problems it may be necessary to carry out additional measurements or structure refinements. However, the purpose of your study may justify the reported deviations and the more serious of these should normally be commented upon in the discussion or experimental section of a paper or in the "special_details" fields of the CIF. checkCIF was carefully designed to identify outliers and unusual parameters, but every test has its limitations and alerts that are not important in a particular case may appear. Conversely, the absence of alerts does not guarantee there are no aspects of the results needing attention. It is up to the individual to critically assess their own results and, if necessary, seek expert advice.

Publication of your CIF in IUCr journals

A basic structural check has been run on your CIF. These basic checks will be run on all CIFs submitted for publication in IUCr journals (*Acta Crystallographica*, *Journal of Applied Crystallography*, *Journal of Synchrotron Radiation*); however, if you intend to submit to *Acta Crystallographica Section C* or *E* or *IUCrData*, you should make sure that full publication checks are run on the final version of your CIF prior to submission.

Publication of your CIF in other journals

Please refer to the *Notes for Authors* of the relevant journal for any special instructions relating to CIF submission.

PLATON version of 14/07/2018; check.def file version of 05/06/2018

Datablock mc221_Final - ellipsoid plot

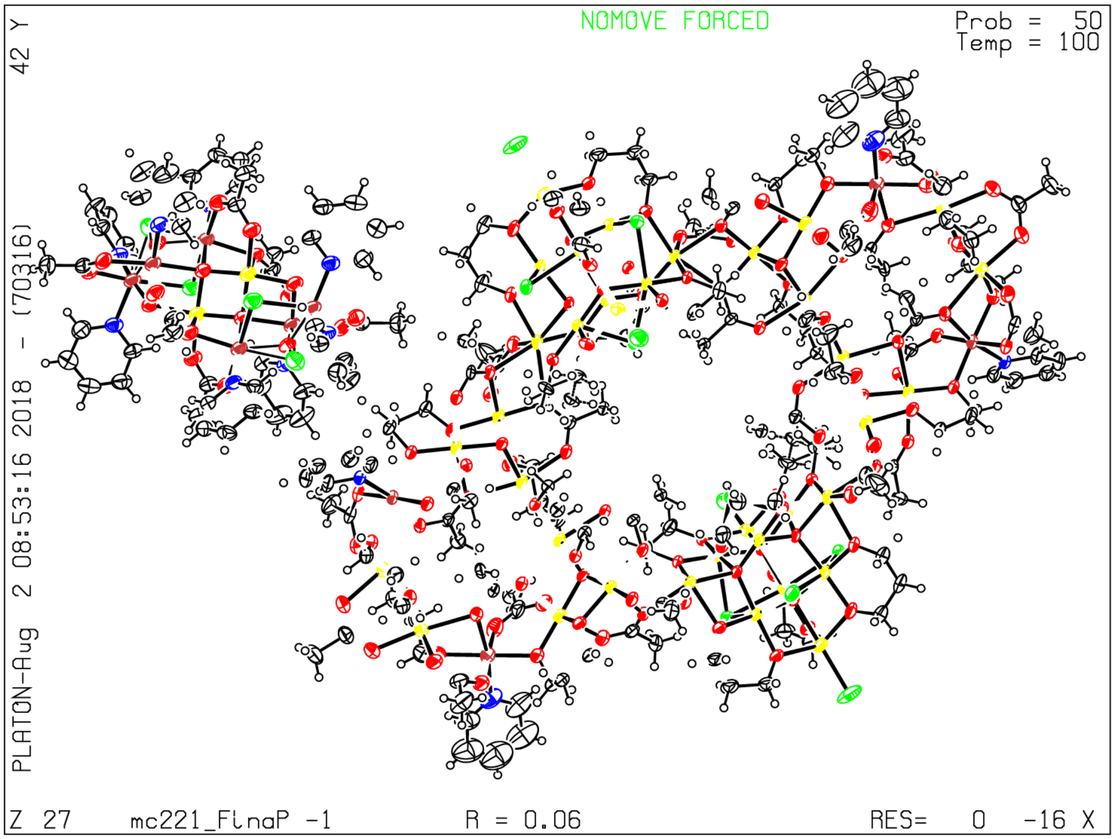

Supplement: Supplementary file 1 [file Data_Sheet_1.PDF]
