# Supplementary material for: Giant Heterometallic [Mn36Ni4]0/2− and [Mn32Co8] “Loops-of-Loops-and-Supertetrahedra” Molecular Aggregates
Source: Front Chem. 2019 Mar 5;7:96. doi: 10.3389/fchem.2019.00096 (PMC6413240; doi:10.3389/fchem.2019.00096)

checkCIF/PLATON report

You have not supplied any structure factors. As a result the full set of tests cannot be run.

THIS REPORT IS FOR GUIDANCE ONLY. IF USED AS PART OF A REVIEW PROCEDURE FOR PUBLICATION, IT SHOULD NOT REPLACE THE EXPERTISE OF AN EXPERIENCED CRYSTALLOGRAPHIC REFEREE.

No syntax errors found.   CIF dictionary   Interpreting this report

Datablock: MC102

```
Bond precision:  C-C = 0.0142 A          Wavelength=0.71073

Cell:           a=47.4626(13)    b=14.0486(4)       c=50.1602(11)
                alpha=90         beta=103.689(3)    gamma=90
Temperature:    100 K
```

	Calculated	Reported
Volume	32495.9(15)	32495.9(15)
Space group	I 2/a	I 2/a
Hall group	-I 2ya	-I 2ya
Moiety formula	C144 H242 Cl10 Mn36 N4 Ni4 O114, 2(C2 H3 N), 0.15(O2), 12(O) [+	C144 H246 Cl10 Mn36 N4 Ni4 O114, 2(C2 H3 N), 12.30(H2 O)
Sum formula	C148 H248 Cl10 Mn36 N6 Ni4 O126.30 [+ solvent]	C148 H276.60 Cl10 Mn36 N6 Ni4 O126.30
Mr	6699.44	6728.33
Dx,g cm-3	1.369	1.375
Z	4	4
Mu (mm-1)	1.722	1.722
F000	13481.6	13596.0
F000’	13547.32	
h,k,lmax	56,16,59	56,16,59
Nref	28639	28529
Tmin,Tmax	0.603,0.709	0.476,1.000
Tmin’	0.591	

```
Correction method= # Reported T Limits: Tmin=0.476 Tmax=1.000
AbsCorr = MULTI-SCAN

Data completeness= 0.996          Theta(max)= 25.000

R(reflections)= 0.0725( 11651)    wR2(reflections)= 0.1851( 28529)

S = 0.933                   Npar= 1561
```

The following ALERTS were generated. Each ALERT has the format
test-name_ALERT_alert-type_alert-level.
Click on the hyperlinks for more details of the test.

Alert level B

```
PLAT430_ALERT_2_B Short Inter D...A Contact  O42      ..O62              2.78 Ang.
                                          -1+x,1+y,z  =      1_465 Check
```

Author Response: This short distance assumes hydrogen-bonding between the crystallization water O62 (with 50% site occupancy) and O42 of an acetate ion; however, due to the disorder of the O62 water molecule it was not possible to locate its hydrogen atoms in the course of the X-ray analysis.

```
PLAT430_ALERT_2_B Short Inter D...A Contact  O68      ..O69              2.65 Ang.
                                    1-x,-1/2+y,3/2-z  =      4_546 Check
```

Author Response: This short distance assumes hydrogen-bonding between the crystallization water O62 (with 50% site occupancy) and O42 of an acetate ion; however, due to the disorder of the O62 water molecule it was not possible to locate its hydrogen atoms in the course of the X-ray analysis.

```
PLAT990_ALERT_1_B Deprecated .res/.hkl Input Style SQUEEZE Job ...          ! Note
```

Alert level C

```
PLAT026_ALERT_3_C Ratio Observed / Unique Reflections (too) Low ..        41% Check
```

Author Response: Several crystals of the title compound were carefully tested on the X-rays at 100 K. The diffraction quality of the crystals has proven to be moderate and structure determination was eventually carried out by means of the best data set collected. As a necessary compromise between resolution and data completeness we have used data up to 50 degrees with a completeness of 99.6%. Therefore, most of the alerts are mainly due to the moderate quality of the difraction data.

```
PLAT094_ALERT_2_C Ratio of Maximum / Minimum Residual Density ....       3.24 Report
```

Author Response: The highest residual density peak is located close to the highly disordered region of the coordinated water molecule O57. Attempts to model this molecule over two orientations with a chemically reasonable geometry were unsuccessful.

```
PLAT220_ALERT_2_C Non-Solvent  Resd 1  C   Ueq(max)/Ueq(min) Range        3.1 Ratio
PLAT241_ALERT_2_C High   'MainMol' Ueq as Compared to Neighbors of        Mn8 Check
PLAT241_ALERT_2_C High   'MainMol' Ueq as Compared to Neighbors of        Cl4 Check
PLAT241_ALERT_2_C High   'MainMol' Ueq as Compared to Neighbors of        C15 Check
PLAT241_ALERT_2_C High   'MainMol' Ueq as Compared to Neighbors of        C16 Check
PLAT241_ALERT_2_C High   'MainMol' Ueq as Compared to Neighbors of        C71 Check
PLAT242_ALERT_2_C Low    'MainMol' Ueq as Compared to Neighbors of        C28 Check
PLAT242_ALERT_2_C Low    'MainMol' Ueq as Compared to Neighbors of        C38 Check
```

```
PLAT250_ALERT_2_C Large U3/U1 Ratio for Average U(i,j) Tensor ....        3.2 Note
PLAT341_ALERT_3_C Low Bond Precision on  C-C Bonds ...............     0.0142 Ang.
```

Alert level G

```
FORMU01_ALERT_2_G  There is a discrepancy between the atom counts in the
            _chemical_formula_sum and the formula from the _atom_site* data.
            Atom count from _chemical_formula_sum:C148 H276.6 Cl10 Mn36 N6 Ni4 O
            Atom count from the _atom_site data:  C148 H248 Cl10 Mn36 N6 Ni4 O12
```

Author Response: Hydrogen atoms not located by the X-ray analysis have been included in the chemical formula; this accounts for the difference between formula and atom_site contents.

```
CELLZ01_ALERT_1_G Difference between formula and atom_site contents detected.
CELLZ01_ALERT_1_G WARNING: H atoms missing from atom site list. Is this intentional?
           From the CIF: _cell_formula_units_Z    4
           From the CIF: _chemical_formula_sum  C148 H276.60 Cl10 Mn36 N6 Ni4 O1
           TEST: Compare cell contents of formula and atom_site data

           atom    Z*formula  cif sites diff
           C         592.00    592.00    0.00
           H        1106.40    992.00  114.40
           Cl         40.00     40.00    0.00
           Mn        144.00    144.00    0.00
           N          24.00     24.00    0.00
           Ni         16.00     16.00    0.00
           O         505.20    505.20    0.00
PLAT002_ALERT_2_G Number of Distance or Angle Restraints on AtSite          6 Note
PLAT003_ALERT_2_G Number of Uiso or Uij Restrained non-H Atoms ...         71 Report
PLAT005_ALERT_5_G No Embedded Refinement Details Found  in the CIF     Please Do !
PLAT041_ALERT_1_G Calc. and Reported SumFormula    Strings  Differ     Please Check
```

Author Response: The missing (not located by a difference synthesis) hydrogen atoms of the coordinated water molecule O57 and the crystallization water molecules O58-O69 have been included in the sum and moiety formula and the Mr, Dx, and F(000) have been modified accordingly. These modifications account for the differences detected in the relevant data items.

```
PLAT042_ALERT_1_G Calc. and Reported MoietyFormula Strings  Differ     Please Check
```

Author Response: See comment above.

```
PLAT068_ALERT_1_G Reported F000 Differs from Calcd (or Missing)...     Please Check
```

Author Response: See comment above.

```
PLAT300_ALERT_4_G Atom Site Occupancy of O60        Constrained at       0.15 Check
PLAT300_ALERT_4_G Atom Site Occupancy of O58A       Constrained at       0.52 Check
PLAT300_ALERT_4_G Atom Site Occupancy of O59        Constrained at        0.6 Check
PLAT300_ALERT_4_G Atom Site Occupancy of O61        Constrained at        0.7 Check
PLAT300_ALERT_4_G Atom Site Occupancy of O62        Constrained at        0.5 Check
PLAT300_ALERT_4_G Atom Site Occupancy of O63        Constrained at        0.5 Check
```

```
PLAT300_ALERT_4_G Atom Site Occupancy of O65        Constrained at        0.5 Check
PLAT300_ALERT_4_G Atom Site Occupancy of O68        Constrained at        0.5 Check
PLAT300_ALERT_4_G Atom Site Occupancy of O69        Constrained at        0.6 Check
PLAT300_ALERT_4_G Atom Site Occupancy of O58B       Constrained at       0.48 Check
PLAT300_ALERT_4_G Atom Site Occupancy of O64        Constrained at        0.4 Check
PLAT300_ALERT_4_G Atom Site Occupancy of O66        Constrained at        0.3 Check
PLAT300_ALERT_4_G Atom Site Occupancy of O67        Constrained at        0.4 Check
PLAT302_ALERT_4_G Anion/Solvent/Minor-Residue Disorder (Resd  3  )       100% Note
PLAT302_ALERT_4_G Anion/Solvent/Minor-Residue Disorder (Resd  4  )       100% Note
PLAT302_ALERT_4_G Anion/Solvent/Minor-Residue Disorder (Resd  5  )       100% Note
PLAT302_ALERT_4_G Anion/Solvent/Minor-Residue Disorder (Resd  6  )       100% Note
PLAT302_ALERT_4_G Anion/Solvent/Minor-Residue Disorder (Resd  7  )       100% Note
PLAT302_ALERT_4_G Anion/Solvent/Minor-Residue Disorder (Resd  8  )       100% Note
PLAT302_ALERT_4_G Anion/Solvent/Minor-Residue Disorder (Resd  9  )       100% Note
PLAT302_ALERT_4_G Anion/Solvent/Minor-Residue Disorder (Resd 10  )       100% Note
PLAT302_ALERT_4_G Anion/Solvent/Minor-Residue Disorder (Resd 11  )       100% Note
PLAT302_ALERT_4_G Anion/Solvent/Minor-Residue Disorder (Resd 12  )       100% Note
PLAT302_ALERT_4_G Anion/Solvent/Minor-Residue Disorder (Resd 13  )       100% Note
PLAT302_ALERT_4_G Anion/Solvent/Minor-Residue Disorder (Resd 14  )       100% Note
PLAT302_ALERT_4_G Anion/Solvent/Minor-Residue Disorder (Resd 15  )       100% Note
PLAT304_ALERT_4_G Non-Integer Number of Atoms in ...... Resd  3          0.30 Check
PLAT304_ALERT_4_G Non-Integer Number of Atoms in ...... Resd  4          0.52 Check
PLAT304_ALERT_4_G Non-Integer Number of Atoms in ...... Resd  5          0.60 Check
PLAT304_ALERT_4_G Non-Integer Number of Atoms in ...... Resd  6          0.70 Check
PLAT304_ALERT_4_G Non-Integer Number of Atoms in ...... Resd  7          0.50 Check
PLAT304_ALERT_4_G Non-Integer Number of Atoms in ...... Resd  8          0.50 Check
PLAT304_ALERT_4_G Non-Integer Number of Atoms in ...... Resd  9          0.50 Check
PLAT304_ALERT_4_G Non-Integer Number of Atoms in ...... Resd 10          0.50 Check
PLAT304_ALERT_4_G Non-Integer Number of Atoms in ...... Resd 11          0.60 Check
PLAT304_ALERT_4_G Non-Integer Number of Atoms in ...... Resd 12          0.48 Check
PLAT304_ALERT_4_G Non-Integer Number of Atoms in ...... Resd 13          0.40 Check
PLAT304_ALERT_4_G Non-Integer Number of Atoms in ...... Resd 14          0.30 Check
PLAT304_ALERT_4_G Non-Integer Number of Atoms in ...... Resd 15          0.40 Check
PLAT311_ALERT_2_G Isolated Disordered Oxygen Atom (No H’s ?) .....       O58A Check
```

Author Response: It was not possible to locate the hydrogen atoms of the disordered lattice water molecules O58-O69 (with occupancies ranging from 15% to 70%) from a difference map.

```
PLAT311_ALERT_2_G Isolated Disordered Oxygen Atom (No H’s ?) .....        O59 Check
```

Author Response: It was not possible to locate the hydrogen atoms of the disordered lattice water molecules O58-O69 (with occupancies ranging from 15% to 70%) from a difference map.

```
PLAT311_ALERT_2_G Isolated Disordered Oxygen Atom (No H’s ?) .....        O61 Check
```

Author Response: It was not possible to locate the hydrogen atoms of the disordered lattice water molecules O58-O69 (with occupancies ranging from 15% to 70%) from a difference map.

```
PLAT311_ALERT_2_G Isolated Disordered Oxygen Atom (No H’s ?) .....        O62 Check
```

Author Response: It was not possible to locate the hydrogen atoms of the disordered lattice water molecules O58-O69 (with occupancies ranging from 15% to 70%) from a difference map.

```
PLAT311_ALERT_2_G Isolated Disordered Oxygen Atom (No H’s ?) .....        O63 Check
```

Author Response: It was not possible to locate the hydrogen atoms of the disordered lattice water molecules O58-O69 (with occupancies ranging from 15% to 70%) from a difference map.

```
PLAT311_ALERT_2_G Isolated Disordered Oxygen Atom (No H’s ?) .....        O65 Check
```

Author Response: It was not possible to locate the hydrogen atoms of the disordered lattice water molecules O58-O69 (with occupancies ranging from 15% to 70%) from a difference map.

```
PLAT311_ALERT_2_G Isolated Disordered Oxygen Atom (No H’s ?) .....        O68 Check
```

Author Response: It was not possible to locate the hydrogen atoms of the disordered lattice water molecules O58-O69 (with occupancies ranging from 15% to 70%) from a difference map.

```
PLAT311_ALERT_2_G Isolated Disordered Oxygen Atom (No H’s ?) .....        O69 Check
```

Author Response: It was not possible to locate the hydrogen atoms of the disordered lattice water molecules O58-O69 (with occupancies ranging from 15% to 70%) from a difference map.

```
PLAT311_ALERT_2_G Isolated Disordered Oxygen Atom (No H’s ?) .....       O58B Check
```

Author Response: It was not possible to locate the hydrogen atoms of the disordered lattice water molecules O58-O69 (with occupancies ranging from 15% to 70%) from a difference map.

```
PLAT311_ALERT_2_G Isolated Disordered Oxygen Atom (No H’s ?) .....        O64 Check
```

Author Response: It was not possible to locate the hydrogen atoms of the disordered lattice water molecules O58-O69 (with occupancies ranging from 15% to 70%) from a difference map.

```
PLAT311_ALERT_2_G Isolated Disordered Oxygen Atom (No H’s ?) .....        O66 Check
```

Author Response: It was not possible to locate the hydrogen atoms of the disordered lattice water molecules O58-O69 (with occupancies ranging from 15% to 70%) from a difference map.

```
PLAT311_ALERT_2_G Isolated Disordered Oxygen Atom (No H's ?) .....        O67 Check
```

Author Response: It was not possible to locate the hydrogen atoms of the disordered lattice water molecules O58-O69 (with occupancies ranging from 15% to 70%) from a difference map.

```
PLAT606_ALERT_4_G VERY LARGE Solvent Accessible VOID(S) in Structure        ! Info
PLAT794_ALERT_5_G Tentative Bond Valency for Ni1       (II)      .       2.07 Info
PLAT794_ALERT_5_G Tentative Bond Valency for Ni2       (II)      .       2.03 Info
PLAT794_ALERT_5_G Tentative Bond Valency for Mn1       (II)      .       2.00 Info
PLAT794_ALERT_5_G Tentative Bond Valency for Mn2       (I)       .       0.91 Info
PLAT794_ALERT_5_G Tentative Bond Valency for Mn3       (I)       .       0.92 Info
PLAT794_ALERT_5_G Tentative Bond Valency for Mn4       (I)       .       0.92 Info
PLAT794_ALERT_5_G Tentative Bond Valency for Mn5       (I)       .       0.94 Info
PLAT794_ALERT_5_G Tentative Bond Valency for Mn6       (II)      .       1.94 Info
PLAT794_ALERT_5_G Tentative Bond Valency for Mn7       (III)     .       3.16 Info
PLAT794_ALERT_5_G Tentative Bond Valency for Mn8       (II)      .       1.74 Info
PLAT794_ALERT_5_G Tentative Bond Valency for Mn9       (I)       .       0.84 Info
PLAT794_ALERT_5_G Tentative Bond Valency for Mn10      (I)       .       0.79 Info
PLAT794_ALERT_5_G Tentative Bond Valency for Mn11      (I)       .       0.81 Info
PLAT794_ALERT_5_G Tentative Bond Valency for Mn12      (I)       .       0.80 Info
PLAT794_ALERT_5_G Tentative Bond Valency for Mn13      (I)       .       0.80 Info
PLAT794_ALERT_5_G Tentative Bond Valency for Mn14      (II)      .       2.12 Info
PLAT794_ALERT_5_G Tentative Bond Valency for Mn15      (I)       .       0.92 Info
PLAT794_ALERT_5_G Tentative Bond Valency for Mn16      (I)       .       0.93 Info
PLAT794_ALERT_5_G Tentative Bond Valency for Mn17      (I)       .       0.92 Info
PLAT794_ALERT_5_G Tentative Bond Valency for Mn18      (I)       .       0.94 Info
PLAT860_ALERT_3_G Number of Least-Squares Restraints .............        243 Note
PLAT869_ALERT_4_G ALERTS Related to the Use of SQUEEZE Suppressed           ! Info
PLAT881_ALERT_1_G No Datum for _diffrn_reflns_av_R_equivalents ...     Please Do !
PLAT899_ALERT_4_G SHELXL97   is Deprecated and Succeeded by SHELXL       2018 Note
```

```
   0 ALERT level A = Most likely a serious problem - resolve or explain
   3 ALERT level B = A potentially serious problem, consider carefully
  12 ALERT level C = Check. Ensure it is not caused by an omission or oversight
  85 ALERT level G = General information/check it is not something unexpected

   7 ALERT type 1 CIF construction/syntax error, inconsistent or missing data
  27 ALERT type 2 Indicator that the structure model may be wrong or deficient
   3 ALERT type 3 Indicator that the structure quality may be low
  42 ALERT type 4 Improvement, methodology, query or suggestion
  21 ALERT type 5 Informative message, check
```

It is advisable to attempt to resolve as many as possible of the alerts in all categories. Often the minor alerts point to easily fixed oversights, errors and omissions in your CIF or refinement strategy, so attention to these fine details can be worthwhile. In order to resolve some of the more serious problems it may be necessary to carry out additional measurements or structure refinements. However, the purpose of your study may justify the reported deviations and the more serious of these should normally be commented upon in the discussion or experimental section of a paper or in the "special_details" fields of the CIF. checkCIF was carefully designed to identify outliers and unusual parameters, but every test has its limitations and alerts that are not important in a particular case may appear. Conversely, the absence of alerts does not guarantee there are no aspects of the results needing attention. It is up to the individual to critically assess their own results and, if necessary, seek expert advice.

Publication of your CIF in IUCr journals

A basic structural check has been run on your CIF. These basic checks will be run on all CIFs submitted for publication in IUCr journals (*Acta Crystallographica*, *Journal of Applied Crystallography*, *Journal of Synchrotron Radiation*); however, if you intend to submit to *Acta Crystallographica Section C* or *E* or *IUCrData*, you should make sure that full publication checks are run on the final version of your CIF prior to submission.

Publication of your CIF in other journals

Please refer to the *Notes for Authors* of the relevant journal for any special instructions relating to CIF submission.

PLATON version of 14/07/2018; check.def file version of 05/06/2018

Datablock MC102 - ellipsoid plot

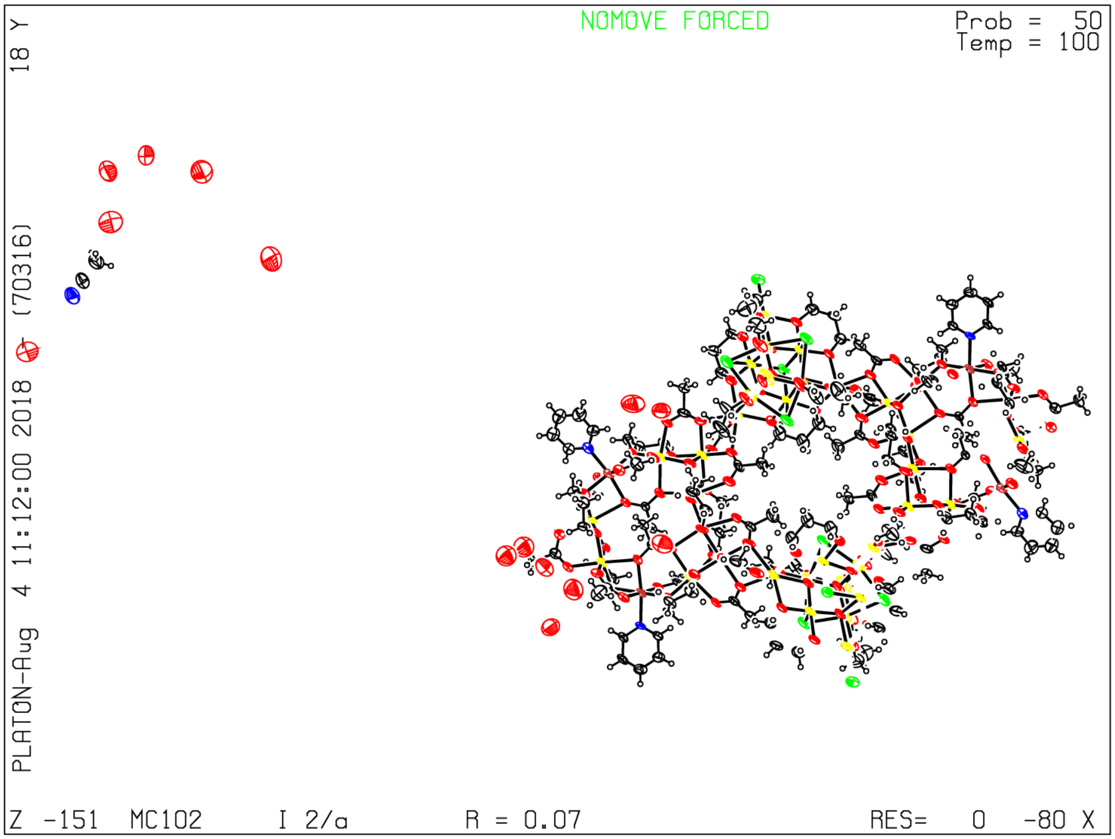

Supplement: Supplementary file 2 [file Data_Sheet_2.PDF]
