# Supplementary material for: Giant Heterometallic [Mn36Ni4]0/2− and [Mn32Co8] “Loops-of-Loops-and-Supertetrahedra” Molecular Aggregates
Source: Front Chem. 2019 Mar 5;7:96. doi: 10.3389/fchem.2019.00096 (PMC6413240; doi:10.3389/fchem.2019.00096)

checkCIF/PLATON report

You have not supplied any structure factors. As a result the full set of tests cannot be run.

THIS REPORT IS FOR GUIDANCE ONLY. IF USED AS PART OF A REVIEW PROCEDURE FOR PUBLICATION, IT SHOULD NOT REPLACE THE EXPERTISE OF AN EXPERIENCED CRYSTALLOGRAPHIC REFEREE.

No syntax errors found.   CIF dictionary   Interpreting this report

Datablock: mn32co8_Final

```
Bond precision:  C-C = 0.0181 A          Wavelength=0.71073

Cell:           a=14.5627(6)       b=21.3308(9)     c=26.2945(10)
                alpha=100.083(3)   beta=94.279(3)   gamma=98.230(4)
Temperature:    100 K
```

	Calculated	Reported
Volume	7917.3(6)	7917.2(6)
Space group	P -1	P -1
Hall group	-P 1	-P 1
Moiety formula	C156 H256 Cl10 Co8 Mn32 N6 O114, 2.4(C2 H5 O), 1.44(C O), 6(O),	C156 H256 Cl10 Co8 Mn32 N6 O114, 3.84(C2 H5 O), 6(O)
Sum formula	C163.68 H275.20 Cl10 Co8 Mn32 N6 O123.84 [+ solvent]	C163.68 H275.20 Cl10 Co8 Mn32 N6 O123.84
Mr	6892.73	6892.70
Dx,g cm-3	1.446	1.446
Z	1	1
Mu (mm-1)	1.790	1.790
F000	3476.0	3476.0
F000’	3492.53	
h,k,lmax	16,24,30	16,24,30
Nref	24901	24788
Tmin,Tmax	0.756,0.882	0.640,1.000
Tmin’	0.751	

```
Correction method= # Reported T Limits: Tmin=0.640 Tmax=1.000
AbsCorr = MULTI-SCAN

Data completeness= 0.995          Theta(max)= 24.000

R(reflections)= 0.0933( 18487)    wR2(reflections)= 0.2497( 24788)

S = 1.060                   Npar= 1595
```

The following ALERTS were generated. Each ALERT has the format
test-name_ALERT_alert-type_alert-level.
Click on the hyperlinks for more details of the test.

Alert level B

```
THETM01_ALERT_3_B  The value of sine(theta_max)/wavelength is less than 0.575
            Calculated sin(theta_max)/wavelength =    0.5723
PLAT220_ALERT_2_B Non-Solvent  Resd 1  C   Ueq(max)/Ueq(min) Range        7.0 Ratio
PLAT306_ALERT_2_B Isolated Oxygen Atom (H-atoms Missing ?) .......        O61 Check
PLAT306_ALERT_2_B Isolated Oxygen Atom (H-atoms Missing ?) .......        O62 Check
PLAT306_ALERT_2_B Isolated Oxygen Atom (H-atoms Missing ?) .......        O63 Check
PLAT341_ALERT_3_B Low Bond Precision on  C-C Bonds ...............    0.01808 Ang.
PLAT430_ALERT_2_B Short Inter D...A Contact  O59      ..O63             2.82 Ang.
                                                      x,y,z  =      1_555 Check
PLAT430_ALERT_2_B Short Inter D...A Contact  O60      ..O62             2.62 Ang.
                                                      x,y,z  =      1_555 Check
```

Alert level C

```
PLAT213_ALERT_2_C Atom O7              has ADP max/min Ratio .....        3.6 prolat
PLAT213_ALERT_2_C Atom O48             has ADP max/min Ratio .....        3.1 oblate
PLAT213_ALERT_2_C Atom C1              has ADP max/min Ratio .....        3.2 prolat
PLAT213_ALERT_2_C Atom C74             has ADP max/min Ratio .....        3.5 prolat
PLAT220_ALERT_2_C Non-Solvent  Resd 1  O   Ueq(max)/Ueq(min) Range        4.0 Ratio
PLAT222_ALERT_3_C Non-Solv.  Resd 1  H   Uiso(max)/Uiso(min) Range        6.1 Ratio
PLAT241_ALERT_2_C High   'MainMol' Ueq as Compared to Neighbors of        C51 Check
PLAT241_ALERT_2_C High   'MainMol' Ueq as Compared to Neighbors of        C57 Check
PLAT241_ALERT_2_C High   'MainMol' Ueq as Compared to Neighbors of        C64 Check
PLAT241_ALERT_2_C High   'MainMol' Ueq as Compared to Neighbors of        C73 Check
PLAT242_ALERT_2_C Low    'MainMol' Ueq as Compared to Neighbors of         N1 Check
PLAT242_ALERT_2_C Low    'MainMol' Ueq as Compared to Neighbors of        C77 Check
PLAT309_ALERT_2_C Single Bonded Oxygen (C-O > 1.3 Ang) ...........        O58 Check
PLAT309_ALERT_2_C Single Bonded Oxygen (C-O > 1.3 Ang) ...........        O60 Check
PLAT309_ALERT_2_C Single Bonded Oxygen (C-O > 1.3 Ang) ...........        O59 Check
PLAT413_ALERT_2_C Short Inter XH3 .. XHn     H22B     ..H81A            2.06 Ang.
                                                    1+x,y,z  =      1_655 Check
PLAT413_ALERT_2_C Short Inter XH3 .. XHn     H26A     ..H34A            2.09 Ang.
                                                 2-x,-y,1-z  =      2_756 Check
PLAT430_ALERT_2_C Short Inter D...A Contact  O23      ..O59             2.89 Ang.
                                                    1+x,y,z  =      1_655 Check
```

Alert level G

```
PLAT002_ALERT_2_G Number of Distance or Angle Restraints on AtSite         18 Note
PLAT003_ALERT_2_G Number of Uiso or Uij Restrained non-H Atoms ...         17 Report
PLAT042_ALERT_1_G Calc. and Reported MoietyFormula Strings  Differ     Please Check
PLAT083_ALERT_2_G SHELXL Second Parameter in WGHT  Unusually Large      96.28 Why ?
PLAT172_ALERT_4_G The CIF-Embedded .res File Contains DFIX Records         10 Report
PLAT174_ALERT_4_G The CIF-Embedded .res File Contains FLAT Records          1 Report
PLAT177_ALERT_4_G The CIF-Embedded .res File Contains DELU Records          1 Report
PLAT186_ALERT_4_G The CIF-Embedded .res File Contains ISOR Records          9 Report
PLAT187_ALERT_4_G The CIF-Embedded .res File Contains RIGU Records          1 Report
PLAT300_ALERT_4_G Atom Site Occupancy of C78A       Constrained at       0.65 Check
PLAT300_ALERT_4_G Atom Site Occupancy of C78B       Constrained at       0.35 Check
PLAT300_ALERT_4_G Atom Site Occupancy of H77A       Constrained at       0.65 Check
PLAT300_ALERT_4_G Atom Site Occupancy of H77B       Constrained at       0.65 Check
PLAT300_ALERT_4_G Atom Site Occupancy of H78A       Constrained at       0.65 Check
PLAT300_ALERT_4_G Atom Site Occupancy of H78B       Constrained at       0.65 Check
PLAT300_ALERT_4_G Atom Site Occupancy of H78C       Constrained at       0.65 Check
```

```
PLAT300_ALERT_4_G Atom Site Occupancy of H77C       Constrained at       0.35 Check
PLAT300_ALERT_4_G Atom Site Occupancy of H77D       Constrained at       0.35 Check
PLAT300_ALERT_4_G Atom Site Occupancy of H78D       Constrained at       0.35 Check
PLAT300_ALERT_4_G Atom Site Occupancy of H78E       Constrained at       0.35 Check
PLAT300_ALERT_4_G Atom Site Occupancy of H78F       Constrained at       0.35 Check
PLAT300_ALERT_4_G Atom Site Occupancy of O58        Constrained at        0.6 Check
PLAT300_ALERT_4_G Atom Site Occupancy of C79        Constrained at        0.6 Check
PLAT300_ALERT_4_G Atom Site Occupancy of C80        Constrained at        0.6 Check
PLAT300_ALERT_4_G Atom Site Occupancy of H79A       Constrained at        0.6 Check
PLAT300_ALERT_4_G Atom Site Occupancy of H79B       Constrained at        0.6 Check
PLAT300_ALERT_4_G Atom Site Occupancy of H79C       Constrained at        0.6 Check
PLAT300_ALERT_4_G Atom Site Occupancy of H80A       Constrained at        0.6 Check
PLAT300_ALERT_4_G Atom Site Occupancy of H80B       Constrained at        0.6 Check
PLAT300_ALERT_4_G Atom Site Occupancy of O60        Constrained at        0.6 Check
PLAT300_ALERT_4_G Atom Site Occupancy of C83        Constrained at        0.6 Check
PLAT300_ALERT_4_G Atom Site Occupancy of C84        Constrained at        0.6 Check
PLAT300_ALERT_4_G Atom Site Occupancy of H83A       Constrained at        0.6 Check
PLAT300_ALERT_4_G Atom Site Occupancy of H83B       Constrained at        0.6 Check
PLAT300_ALERT_4_G Atom Site Occupancy of H84A       Constrained at        0.6 Check
PLAT300_ALERT_4_G Atom Site Occupancy of H84B       Constrained at        0.6 Check
PLAT300_ALERT_4_G Atom Site Occupancy of H84C       Constrained at        0.6 Check
PLAT300_ALERT_4_G Atom Site Occupancy of O59        Constrained at       0.72 Check
PLAT300_ALERT_4_G Atom Site Occupancy of C82        Constrained at       0.72 Check
PLAT300_ALERT_4_G Atom Site Occupancy of C81A       Constrained at       0.38 Check
PLAT300_ALERT_4_G Atom Site Occupancy of H81A       Constrained at       0.38 Check
PLAT300_ALERT_4_G Atom Site Occupancy of H81B       Constrained at       0.38 Check
PLAT300_ALERT_4_G Atom Site Occupancy of H81C       Constrained at       0.38 Check
PLAT300_ALERT_4_G Atom Site Occupancy of C81B       Constrained at       0.34 Check
PLAT300_ALERT_4_G Atom Site Occupancy of H81D       Constrained at       0.34 Check
PLAT300_ALERT_4_G Atom Site Occupancy of H81E       Constrained at       0.34 Check
PLAT300_ALERT_4_G Atom Site Occupancy of H81F       Constrained at       0.34 Check
PLAT300_ALERT_4_G Atom Site Occupancy of H82A       Constrained at       0.38 Check
PLAT300_ALERT_4_G Atom Site Occupancy of H82B       Constrained at       0.38 Check
PLAT300_ALERT_4_G Atom Site Occupancy of H82C       Constrained at       0.34 Check
PLAT300_ALERT_4_G Atom Site Occupancy of H82D       Constrained at       0.34 Check
PLAT301_ALERT_3_G Main Residue  Disorder ..............(Resd  1  )         1% Note
PLAT302_ALERT_4_G Anion/Solvent/Minor-Residue Disorder (Resd  2  )       100% Note
PLAT302_ALERT_4_G Anion/Solvent/Minor-Residue Disorder (Resd  3  )       100% Note
PLAT302_ALERT_4_G Anion/Solvent/Minor-Residue Disorder (Resd  4  )       100% Note
PLAT302_ALERT_4_G Anion/Solvent/Minor-Residue Disorder (Resd  8  )       100% Note
PLAT302_ALERT_4_G Anion/Solvent/Minor-Residue Disorder (Resd  9  )       100% Note
PLAT304_ALERT_4_G Non-Integer Number of Atoms in ...... Resd  2          4.80 Check
PLAT304_ALERT_4_G Non-Integer Number of Atoms in ...... Resd  3          4.80 Check
PLAT304_ALERT_4_G Non-Integer Number of Atoms in ...... Resd  4          1.44 Check
PLAT304_ALERT_4_G Non-Integer Number of Atoms in ...... Resd  8          1.52 Check
PLAT304_ALERT_4_G Non-Integer Number of Atoms in ...... Resd  9          1.36 Check
PLAT304_ALERT_4_G Non-Integer Number of Atoms in ...... Resd 10          0.38 Check
PLAT304_ALERT_4_G Non-Integer Number of Atoms in ...... Resd 11          0.38 Check
PLAT304_ALERT_4_G Non-Integer Number of Atoms in ...... Resd 12          0.34 Check
PLAT304_ALERT_4_G Non-Integer Number of Atoms in ...... Resd 13          0.34 Check
PLAT380_ALERT_4_G Incorrectly? Oriented X(sp2)-Methyl Moiety .....         C2 Check
PLAT380_ALERT_4_G Incorrectly? Oriented X(sp2)-Methyl Moiety .....         C4 Check
PLAT380_ALERT_4_G Incorrectly? Oriented X(sp2)-Methyl Moiety .....         C6 Check
PLAT380_ALERT_4_G Incorrectly? Oriented X(sp2)-Methyl Moiety .....         C8 Check
PLAT380_ALERT_4_G Incorrectly? Oriented X(sp2)-Methyl Moiety .....        C10 Check
PLAT380_ALERT_4_G Incorrectly? Oriented X(sp2)-Methyl Moiety .....        C12 Check
PLAT380_ALERT_4_G Incorrectly? Oriented X(sp2)-Methyl Moiety .....        C14 Check
PLAT380_ALERT_4_G Incorrectly? Oriented X(sp2)-Methyl Moiety .....        C16 Check
PLAT380_ALERT_4_G Incorrectly? Oriented X(sp2)-Methyl Moiety .....        C18 Check
PLAT380_ALERT_4_G Incorrectly? Oriented X(sp2)-Methyl Moiety .....        C20 Check
PLAT380_ALERT_4_G Incorrectly? Oriented X(sp2)-Methyl Moiety .....        C22 Check
PLAT380_ALERT_4_G Incorrectly? Oriented X(sp2)-Methyl Moiety .....        C24 Check
```

```
PLAT380_ALERT_4_G Incorrectly? Oriented X(sp2)-Methyl Moiety .....        C26 Check
PLAT380_ALERT_4_G Incorrectly? Oriented X(sp2)-Methyl Moiety .....        C28 Check
PLAT432_ALERT_2_G Short Inter X...Y Contact  O26      ..C70              2.99 Ang.
                                               2-x,1-y,1-z  =      2_766 Check
PLAT606_ALERT_4_G VERY LARGE Solvent Accessible VOID(S) in Structure        ! Info
PLAT764_ALERT_4_G Overcomplete CIF Bond List Detected (Rep/Expd) .       1.16 Ratio
PLAT794_ALERT_5_G Tentative Bond Valency for Co1       (II)      .       2.03 Info
PLAT794_ALERT_5_G Tentative Bond Valency for Co2       (II)      .       2.01 Info
PLAT794_ALERT_5_G Tentative Bond Valency for Co3       (II)      .       1.97 Info
PLAT794_ALERT_5_G Tentative Bond Valency for Mn2       (I)       .       0.91 Info
PLAT794_ALERT_5_G Tentative Bond Valency for Mn3       (I)       .       0.93 Info
PLAT794_ALERT_5_G Tentative Bond Valency for Mn4       (I)       .       0.91 Info
PLAT794_ALERT_5_G Tentative Bond Valency for Mn5       (I)       .       0.91 Info
PLAT794_ALERT_5_G Tentative Bond Valency for Mn6       (I)       .       0.91 Info
PLAT794_ALERT_5_G Tentative Bond Valency for Mn7       (I)       .       0.94 Info
PLAT794_ALERT_5_G Tentative Bond Valency for Mn9       (II)      .       1.97 Info
PLAT794_ALERT_5_G Tentative Bond Valency for Mn10      (I)       .       0.82 Info
PLAT794_ALERT_5_G Tentative Bond Valency for Mn11      (III)     .       3.14 Info
PLAT794_ALERT_5_G Tentative Bond Valency for Mn13      (III)     .       3.19 Info
PLAT794_ALERT_5_G Tentative Bond Valency for Mn14      (I)       .       0.82 Info
PLAT794_ALERT_5_G Tentative Bond Valency for Mn15      (I)       .       0.79 Info
PLAT794_ALERT_5_G Tentative Bond Valency for Mn16      (II)      .       1.99 Info
PLAT860_ALERT_3_G Number of Least-Squares Restraints .............        124 Note
PLAT869_ALERT_4_G ALERTS Related to the Use of SQUEEZE Suppressed           ! Info
PLAT933_ALERT_2_G Number of OMIT Records in Embedded .res File ...         41 Note
```

---

  0 **ALERT level A** = Most likely a serious problem - resolve or explain
  8 **ALERT level B** = A potentially serious problem, consider carefully
 18 **ALERT level C** = Check. Ensure it is not caused by an omission or oversight
102 **ALERT level G** = General information/check it is not something unexpected

```
  1 ALERT type 1 CIF construction/syntax error, inconsistent or missing data
 28 ALERT type 2 Indicator that the structure model may be wrong or deficient
  5 ALERT type 3 Indicator that the structure quality may be low
 78 ALERT type 4 Improvement, methodology, query or suggestion
 16 ALERT type 5 Informative message, check
```

It is advisable to attempt to resolve as many as possible of the alerts in all categories. Often the minor alerts point to easily fixed oversights, errors and omissions in your CIF or refinement strategy, so attention to these fine details can be worthwhile. In order to resolve some of the more serious problems it may be necessary to carry out additional measurements or structure refinements. However, the purpose of your study may justify the reported deviations and the more serious of these should normally be commented upon in the discussion or experimental section of a paper or in the "special_details" fields of the CIF. checkCIF was carefully designed to identify outliers and unusual parameters, but every test has its limitations and alerts that are not important in a particular case may appear. Conversely, the absence of alerts does not guarantee there are no aspects of the results needing attention. It is up to the individual to critically assess their own results and, if necessary, seek expert advice.

Publication of your CIF in IUCr journals

A basic structural check has been run on your CIF. These basic checks will be run on all CIFs submitted for publication in IUCr journals (*Acta Crystallographica*, *Journal of Applied Crystallography*, *Journal of Synchrotron Radiation*); however, if you intend to submit to *Acta Crystallographica Section C* or *E* or *IUCrData*, you should make sure that full publication checks are run on the final version of your CIF prior to submission.

Publication of your CIF in other journals

Please refer to the *Notes for Authors* of the relevant journal for any special instructions relating to CIF submission.

PLATON version of 14/07/2018; check.def file version of 05/06/2018

Datablock mn32co8_Final - ellipsoid plot

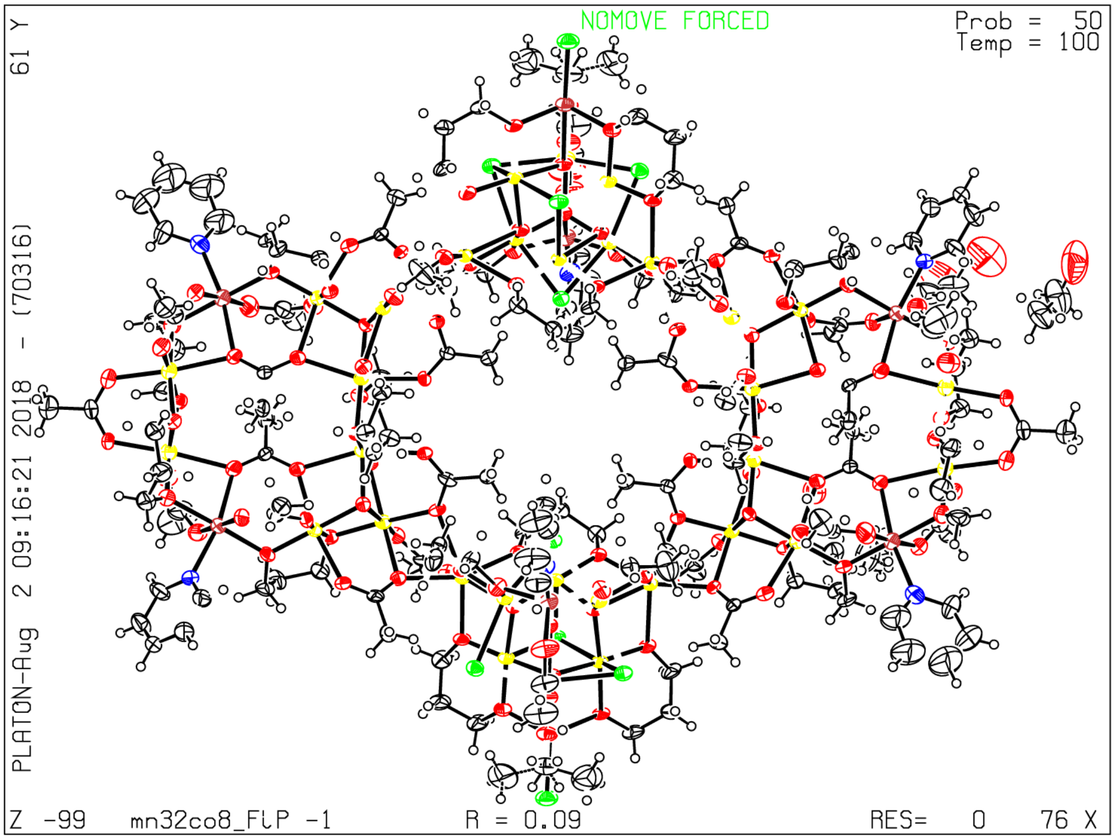

Supplement: Supplementary file 3 [file Data_Sheet_3.PDF]
