# Supplementary material for: Giant Heterometallic [Mn36Ni4]0/2− and [Mn32Co8] “Loops-of-Loops-and-Supertetrahedra” Molecular Aggregates
Source: Front Chem. 2019 Mar 5;7:96. doi: 10.3389/fchem.2019.00096 (PMC6413240; doi:10.3389/fchem.2019.00096)

checkCIF/PLATON report

You have not supplied any structure factors. As a result the full set of tests cannot be run.

THIS REPORT IS FOR GUIDANCE ONLY. IF USED AS PART OF A REVIEW PROCEDURE FOR PUBLICATION, IT SHOULD NOT REPLACE THE EXPERTISE OF AN EXPERIENCED CRYSTALLOGRAPHIC REFEREE.

No syntax errors found. CIF dictionary Interpreting this report

Datablock: mc930_Final

Bond precision: C-C = 0.0116 A Wavelength=0.71073

Cell:	a=19.6744(17)	b=16.2048(13)	c=25.645(5)
	alpha=90	beta=98.804(12)	gamma=90
Temperature:	100 K		

	Calculated	Reported
Volume	8079.8(19)	8079.7(17)
Space group	I 2/a	I 2/a
Hall group	-I 2ya	-I 2ya
Moiety formula	C52 H58 Cl2 Mn2 N8 Ni6 O18, Cl O4, O [+ solvent]	C52 H58 Cl2 Mn2 N8 Ni6 O18, Cl O4, O
Sum formula	C52 H58 Cl3 Mn2 N8 Ni6 O23 [+ solvent]	C52 H58 Cl3 Mn2 N8 Ni6 O23
Mr	1731.43	1731.55
Dx,g cm-3	1.423	1.423
Z	4	4
Mu (mm-1)	1.833	1.833
F000	3516.0	3516.0
F000’	3529.89	
h,k,lmax	24,20,32	24,20,32
Nref	8376	8349
Tmin,Tmax		0.766,1.000
Tmin’		

Correction method= # Reported T Limits: Tmin=0.766 Tmax=1.000
AbsCorr = MULTI-SCAN

Data completeness= 0.997 Theta(max)= 26.500

R(reflections)= 0.0611(5514) wR2(reflections)= 0.1992(8349)

S = 1.013 Npar= 430

The following ALERTS were generated. Each ALERT has the format
test-name_ALERT_alert-type_alert-level.
Click on the hyperlinks for more details of the test.

Alert level B

```
PLAT232_ALERT_2_B Hirshfeld Test Diff (M-X) Ni3      --O1       .       27.4 s.u.
PLAT430_ALERT_2_B Short Inter D...A Contact  O2       ..O12              2.70 Ang.
                                                      x,y,z  =      1_555 Check
```

Alert level C

```
PLAT053_ALERT_1_C Minimum Crystal Dimension Missing (or Error) ...     Please Check
PLAT054_ALERT_1_C Medium  Crystal Dimension Missing (or Error) ...     Please Check
PLAT055_ALERT_1_C Maximum Crystal Dimension Missing (or Error) ...     Please Check
PLAT213_ALERT_2_C Atom O1              has ADP max/min Ratio .....        3.1 prolat
PLAT220_ALERT_2_C Non-Solvent  Resd 1  C   Ueq(max)/Ueq(min) Range        3.3 Ratio
PLAT230_ALERT_2_C Hirshfeld Test Diff for   N4       --C20       .        5.5 s.u.
PLAT234_ALERT_4_C Large Hirshfeld Difference N1       --C5               0.19 Ang.
PLAT234_ALERT_4_C Large Hirshfeld Difference C7       --C8               0.17 Ang.
PLAT234_ALERT_4_C Large Hirshfeld Difference C17      --C18              0.18 Ang.
PLAT234_ALERT_4_C Large Hirshfeld Difference C18      --C19              0.19 Ang.
PLAT241_ALERT_2_C High   'MainMol' Ueq as Compared to Neighbors of         C4 Check
PLAT241_ALERT_2_C High   'MainMol' Ueq as Compared to Neighbors of         C5 Check
PLAT241_ALERT_2_C High   'MainMol' Ueq as Compared to Neighbors of         C8 Check
PLAT242_ALERT_2_C Low    'MainMol' Ueq as Compared to Neighbors of         N1 Check
PLAT341_ALERT_3_C Low Bond Precision on  C-C Bonds ...............    0.01163 Ang.
PLAT413_ALERT_2_C Short Inter XH3 .. XHn     H26C     ..H26C             2.06 Ang.
                                                3/2-x,y,1-z  =      2_656 Check
```

Alert level G

```
PLAT002_ALERT_2_G Number of Distance or Angle Restraints on AtSite          7 Note
PLAT003_ALERT_2_G Number of Uiso or Uij Restrained non-H Atoms ...          8 Report
PLAT072_ALERT_2_G SHELXL First  Parameter in WGHT  Unusually Large       0.13 Report
PLAT152_ALERT_1_G The Supplied and Calc. Volume s.u. Differ by ...          2 Units
PLAT172_ALERT_4_G The CIF-Embedded .res File Contains DFIX Records          5 Report
PLAT186_ALERT_4_G The CIF-Embedded .res File Contains ISOR Records          3 Report
PLAT187_ALERT_4_G The CIF-Embedded .res File Contains RIGU Records          3 Report
PLAT232_ALERT_2_G Hirshfeld Test Diff (M-X) Ni1      --O1_a      .        8.3 s.u.
PLAT232_ALERT_2_G Hirshfeld Test Diff (M-X) Ni2      --O1_a      .        7.6 s.u.
PLAT232_ALERT_2_G Hirshfeld Test Diff (M-X) Ni3      --Cl1_a     .        5.0 s.u.
PLAT244_ALERT_4_G Low    'Solvent' Ueq as Compared to Neighbors of        Cl2 Check
PLAT300_ALERT_4_G Atom Site Occupancy of O12        Constrained at        0.5 Check
PLAT302_ALERT_4_G Anion/Solvent/Minor-Residue Disorder (Resd  3  )       100% Note
PLAT304_ALERT_4_G Non-Integer Number of Atoms in ...... Resd  3          0.50 Check
PLAT311_ALERT_2_G Isolated Disordered Oxygen Atom (No H's ?) .....        O12 Check
PLAT380_ALERT_4_G Incorrectly? Oriented X(sp2)-Methyl Moiety .....        C22 Check
PLAT380_ALERT_4_G Incorrectly? Oriented X(sp2)-Methyl Moiety .....        C24 Check
PLAT380_ALERT_4_G Incorrectly? Oriented X(sp2)-Methyl Moiety .....        C26 Check
PLAT606_ALERT_4_G VERY LARGE Solvent Accessible VOID(S) in Structure        ! Info
PLAT764_ALERT_4_G Overcomplete CIF Bond List Detected (Rep/Expd) .       1.15 Ratio
PLAT794_ALERT_5_G Tentative Bond Valency for Ni1       (II)      .       1.87 Info
PLAT794_ALERT_5_G Tentative Bond Valency for Ni2       (II)      .       1.90 Info
PLAT794_ALERT_5_G Tentative Bond Valency for Ni3       (II)      .       1.69 Info
PLAT794_ALERT_5_G Tentative Bond Valency for Mn1       (III)     .       3.13 Info
PLAT860_ALERT_3_G Number of Least-Squares Restraints .............         55 Note
PLAT869_ALERT_4_G ALERTS Related to the Use of SQUEEZE Suppressed           ! Info
PLAT933_ALERT_2_G Number of OMIT Records in Embedded .res File ...          6 Note
```

```
 0 ALERT level A = Most likely a serious problem - resolve or explain
 2 ALERT level B = A potentially serious problem, consider carefully
16 ALERT level C = Check. Ensure it is not caused by an omission or oversight
27 ALERT level G = General information/check it is not something unexpected

 4 ALERT type 1 CIF construction/syntax error, inconsistent or missing data
18 ALERT type 2 Indicator that the structure model may be wrong or deficient
 2 ALERT type 3 Indicator that the structure quality may be low
17 ALERT type 4 Improvement, methodology, query or suggestion
 4 ALERT type 5 Informative message, check
```

It is advisable to attempt to resolve as many as possible of the alerts in all categories. Often the minor alerts point to easily fixed oversights, errors and omissions in your CIF or refinement strategy, so attention to these fine details can be worthwhile. In order to resolve some of the more serious problems it may be necessary to carry out additional measurements or structure refinements. However, the purpose of your study may justify the reported deviations and the more serious of these should normally be commented upon in the discussion or experimental section of a paper or in the "special_details" fields of the CIF. checkCIF was carefully designed to identify outliers and unusual parameters, but every test has its limitations and alerts that are not important in a particular case may appear. Conversely, the absence of alerts does not guarantee there are no aspects of the results needing attention. It is up to the individual to critically assess their own results and, if necessary, seek expert advice.

Publication of your CIF in IUCr journals

A basic structural check has been run on your CIF. These basic checks will be run on all CIFs submitted for publication in IUCr journals (*Acta Crystallographica*, *Journal of Applied Crystallography*, *Journal of Synchrotron Radiation*); however, if you intend to submit to *Acta Crystallographica Section C* or *E* or *IUCrData*, you should make sure that full publication checks are run on the final version of your CIF prior to submission.

Publication of your CIF in other journals

Please refer to the *Notes for Authors* of the relevant journal for any special instructions relating to CIF submission.

PLATON version of 14/07/2018; check.def file version of 05/06/2018

Datablock mc930_Final - ellipsoid plot

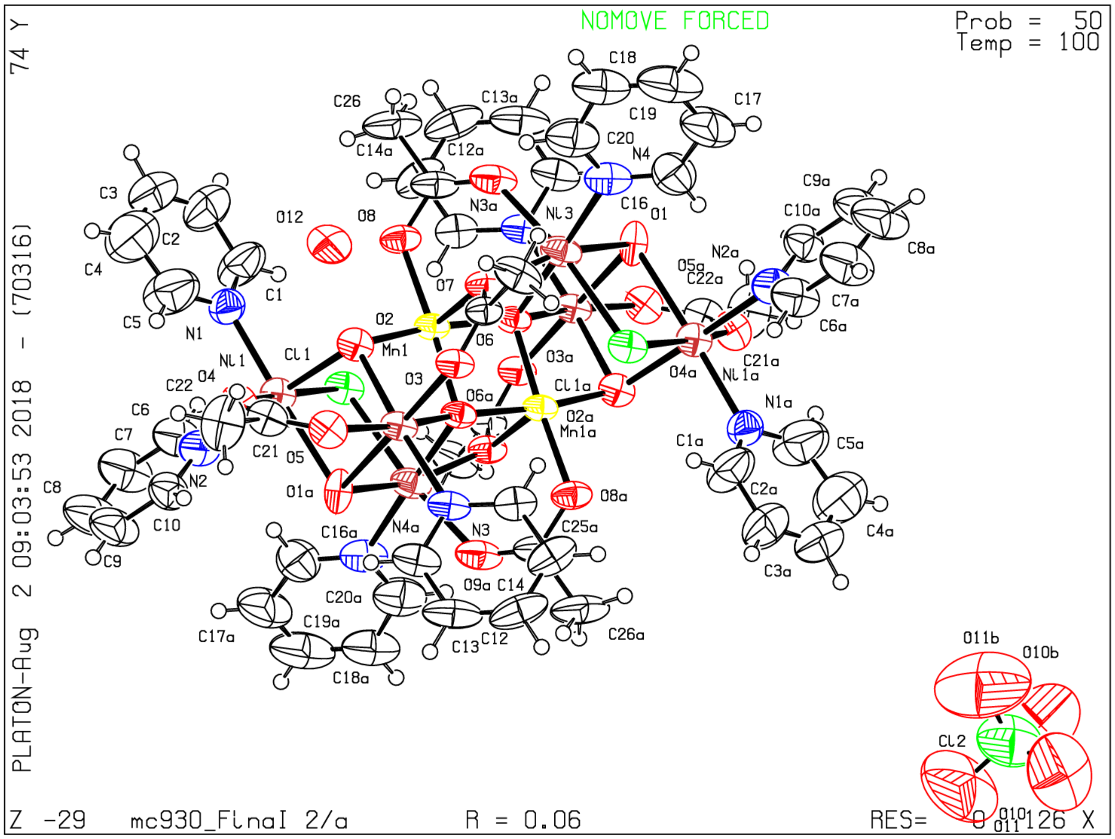

Supplement: Supplementary file 4 [file Data_Sheet_4.PDF]
