# Supplementary material for: Giant Heterometallic [Mn36Ni4]0/2− and [Mn32Co8] “Loops-of-Loops-and-Supertetrahedra” Molecular Aggregates
Source: Front Chem. 2019 Mar 5;7:96. doi: 10.3389/fchem.2019.00096 (PMC6413240; doi:10.3389/fchem.2019.00096)
Supplement: Supplementary file 5 [file Data_Sheet_5.docx]

Supplementary Material

**Giant Heterometallic [Mn_36_Ni_4_]^0/2-^ and [Mn_32_Co_8_] ‘Loops-of-Loops-and-Supertetrahedra’ Molecular Aggregates**

## Maria C. Charalambous^1^, Eleni E. Moushi^1,2^, Tu N.Nguyen^3^, Constantina Papatriantafyllopoulou^1,3,4^, Vassilios Nastopoulos^5^, George Christou^3^, Anastasios J. Tasiopoulos^1,*^

^1^ Department of Chemistry, University of Cyprus 1678 Nicosia, Cyprus.

^2^ Present address: Department of Life Sciences, School of Sciences, European University of Cyprus, 1516 Nicosia, Cyprus

^3^ Department of Chemistry, University of Florida, Gainesville, FL 32611-7200, USA.

^4^ Present address: School of Chemistry, National University of Ireland Galway, Galway, Ireland.

^5^ Department of Chemistry, University of Patras, 26500 Patras, Greece.

*** Correspondence:**

Anastasios J. Tasiopoulos

E-mail: atasio@ucy.ac.cy

**Table of Contents**

Page S2: Table of Contents

Page S3: Materials and Methods/ Table

Pages S4-S19: Results/Crystal Structures/ Tables and Figures

Pages S20-S24: Results/Magnetic Properties/ Magnetism Plots

**Materials and Methods**

**Table S1.**Crystallographic data for complexes (**1**)-(**4**)

| **Param.** | (**1**) | (**2**)·2CH_3_CN·12.30H_2_O | (**3**)·3.84 EtOH·6H_2_O | (**4**) |
| --- | --- | --- | --- | --- |
| Formula | Mn_38_Ni_10_Cl_15_O_129_N_12_C_196_H_300_ | Mn_36_Ni_4_Cl_10_O_126.30_N_6_C_148_H_276.60_ | Mn_32_Co_10_Cl_10_O_123.84_N_6_C_163.68_H_275.20_ | Mn_2_Ni_6_Cl_3_O_23_N_8_C_52_H_58_ |
| FW (g mol^-1^) | 8094.86 | 6728.32 | 6892.70 | 1731.55 |
| Space group | P$\bar{1}$ | I 2/a | P$\bar{1}$ | I 2/a |
| α (Å) | 19.2325(3) | 47.463(2) | 14.5627(6) | 19.675(2) |
| b (Å) | 21.5359(5) | 14.0486(4) | 21.3308 (9) | 16.205(1) |
| c (Å) | 26.0165(6) | 50.160(2) | 26.2945 (10) | 25.645(5) |
| α (°) | 85.877(3) | 90.00 | 100.083 (3) | 90.00 |
| β (°) | 71.202(2) | 103.689(3) | 94.279 (3) | 98.80 (1) |
| γ (°) | 67.749(2) | 90.00 | 98.230 (4) | 90.00 |
| V (Å^3^) | 9425.9(4) | 32495.9(15) | 7917.2(6) | 8079.8(2) |
| Z | 1 | 4 | 1 | 4 |
| T (Κ) | 100(2) | 100(2) | 100(2) | 100(2) |
| radiation (λ) (Å) | Mo Κα (0.71073) | Mo Κα (0.71073) | Mo Kα (0.71073) | Mo Kα (0.71073) |
| *ρ_calcd_*(g/cm^3^) | 1.426 | 1.375 | 1.446 | 1.423 |
| μ (mm^-1^) | 1.883 | 1.722 | 1.790 | 1.833 |
| Measd/ indep. refl. (R_int_) | 107253/36964 (0.0517) | 28529/28529 (0.0000) | 50384/24788 (0.0668) | 27902/8349(0.0400) |
| Obs. refl.^a^ | 21597 | 11651 | 18487 | 5514 |
| R_1_ (%)^a,b^ | 5.85 | 7.25 | 9.33 | 6.11 |
| wR_2_ (%) ^c, d^ | 15.07 | 17.02 | 22.80 | 18.37 |

^a^*Ι*> 2σ(*Ι*), ^b^ R_1_ = 100Σ(║F_0_│-│F_c_║)/ Σ │ F_0_│,^c^ all data,^d^ wR2 = 100[Σ[w(F_0_^2^-F_c_^2^)^2^ / Σ [w (F_0_^2^)^2^]]^1/2^, w = 1/σ^2^(F_0_^2^) + [(ap)^2^ + bP], where P = [max (F_0_^2^, 0) + 2F_c_^2^]/3

**Results/Crystal Structures**

**Table S2.** Selected Interatomic Distances (Å) and angles (°) for (**1**)

| Mn(1)-O(10) | 1.857(4) | Mn(5)-O(17) | 1.887(4) | Mn(9)-O(49) | 2.155(4) | Mn(13)-O(27) | 2.210(3) | Mn(17)-O(29) | 2.133(3) |
| --- | --- | --- | --- | --- | --- | --- | --- | --- | --- |
| Mn(1)-O(11) | 1.919(4) | Mn(5)-O(2) | 1.902(3) | Mn(9)-O(23) | 2.207(4) | Mn(13)-O(28) | 2.231(4) | Mn(17)-O(22) | 2.133(4) |
| Mn(1)-O(21) | 1.940(4) | Mn(5)-O(16) | 1.924(3) | Mn(9)-O(32) | 2.222(3) | Mn(13)-O(33) | 2.246(4) | Mn(17)-O(31) | 2.178(4) |
| Mn(1)-O(34) | 1.946(4) | Mn(5)-O(15) | 1.967(4) | Mn(9)-O(24) | 2.229(4) | Mn(13)-O(5) | 2.294(3) | Mn(17)-O(6) | 2.182(4) |
| Mn(1)-O(59) | 2.172(4) | Mn(5)-O(46) | 2.180(4) | Mn(9)-O(3) | 2.296(4) | Mn(13)-O(41) | 2.301(4) | Mn(17)-Cl(6) | 2.3485(18) |
| Mn(1)-O(38) | 2.338(3) | Mn(5)-O(54) | 2.357(3) | Mn(9)-O(50) | 2.369(3) | Mn(13)-O(43) | 2.463(3) | Mn(18)-O(30) | 1.894(4) |
| Mn(2)-O(12) | 1.862(4) | Mn(6)-O(2) | 1.877(3) | Mn(10)-O(24) | 1.875(4) | Mn(14)-O(33) | 1.869(3) | Mn(18)-O(31) | 1.897(4) |
| Mn(2)-O(1) | 1.935(4) | Mn(6)-O(48) | 1.927(4) | Mn(10)-O(25) | 1.894(4) | Mn(14)-O(32) | 1.881(4) | Mn(18)-O(6) | 1.907(3) |
| Mn(2)-O(13) | 1.957(4) | Mn(6)-O(18) | 1.958(3) | Mn(10)-O(3) | 1.914(3) | Mn(14)-O(5) | 1.923(4) | Mn(18)-O(4) | 1.917(4) |
| Mn(2)-O(37) | 1.963(4) | Mn(6)-O(47) | 1.979(3) | Mn(10)-O(4) | 1.928(4) | Mn(14)-O(3) | 1.933(3) | Mn(18)-Cl(3) | 2.613(2) |
| Mn(2)-O(40) | 2.143(4) | Mn(6)-O(17) | 2.182(4) | Mn(10)-Cl(1) | 2.673(2) | Mn(14)-Cl(2) | 2.702(2) | Mn(18)-Cl(1) | 2.676(2) |
| Mn(2)-O(39) | 2.237(3) | Mn(6)-O(50) | 2.203(4) | Mn(10)-Cl(2) | 2.675(2) | Mn(14)-Cl(4) | 2.753(2) | Mn(19)-O(8)#0 | 1.877(4) |
| Mn(3)-O(1) | 1.868(4) | Mn(7)-O(19) | 1.860(3) | Mn(11)-O(26) | 2.130(3) | Mn(15)-O(23) | 1.876(4) | Mn(19)-O(8) | 1.888(4) |
| Mn(3)-O(44) | 1.918(4) | Mn(7)-O(2) | 1.913(3) | Mn(11)-O(25) | 2.135(4) | Mn(15)-O(22) | 1.892(4) | Mn(19)-O(64) | 1.933(4) |
| Mn(3)-O(13) | 1.950(4) | Mn(7)-O(18) | 1.946(4) | Mn(11)-O(30) | 2.159(4) | Mn(15)-O(3) | 1.909(4) | Mn(19)-O(7) | 1.948(4) |
| Mn(3)-O(43) | 2.005(3) | Mn(7)-O(52) | 1.981(4) | Mn(11)-O(4) | 2.176(3) | Mn(15)-O(6) | 1.918(4) | Mn(19)-O(63) | 2.210(4) |
| Mn(3)-O(14) | 2.194(4) | Mn(7)-O(51) | 2.177(4) | Mn(11)-Cl(5) | 2.327(2) | Mn(15)-Cl(4) | 2.646(1) | Mn(19)-Cl(7) | 2.768(2) |
| Mn(3)-O(41) | 2.202(4) | Mn(7)-O(54) | 2.225(3) | Mn(12)-O(27) | 1.857(3) | Mn(15)-Cl(1) | 2.765(1) |  |  |
| Mn(4)-O(14) | 1.888(4) | Mn(8)-O(20) | 1.862(4) | Mn(12)-O(26) | 1.889(3) | Mn(16)-O(28) | 1.878(3) |  |  |
| Mn(4)-O(1) | 1.901(3) | Mn(8)-O(21) | 1.929(4) | Mn(12)-O(5) | 1.915(3) | Mn(16)-O(29) | 1.887(4) |  |  |
| Mn(4)-O(15) | 1.932(3) | Mn(8)-O(11) | 1.936(4) | Mn(12)-O(4) | 1.927(3) | Mn(16)-O(5) | 1.919(4) |  |  |
| Mn(4)-O(16) | 1.949(4) | Mn(8)-O(57) | 1.944(4) | Mn(12)-Cl(2) | 2.696(2) | Mn(16)-O(6) | 1.953(3) |  |  |
| Mn(4)-O(42) | 2.184(3) | Mn(8)-O(58) | 2.159(4) | Mn(12)-Cl(3) | 2.7077(19) | Mn(16)-Cl(4) | 2.6387(17) |  |  |
| Mn(4)-O(39) | 2.361(4) | Mn(8)-O(55) | 2.369(3) | Mn(13)-O(45) | 2.201(4) | Mn(16)-Cl(3) | 2.7040(16) |  |  |
|  |  |  |  |  |  |  |  |  |  |
| Ni(1)-O(36) | 2.021(4) | Ni(2)-O(53) | 2.030(4) | Ni(3)-O(7) | 2.025(4) | Ni(4)-O(61) | 2.020(4) | Ni(5)-O(9) | 2.00(1) |
| Ni(1)-O(12) | 2.038(4) | Ni(2)-O(19) | 2.036(3) | Ni(3)-O(60) | 2.022(4) | Ni(4)-O(62) | 2.035(4) | Ni(5)-N(6) | 2.030(5) |
| Ni(1)-O(35) | 2.048(4) | Ni(2)-O(56) | 2.065(4) | Ni(3)-N(4) | 2.060(5) | Ni(4)-O(9) | 2.04(1) | Ni(5)-O(8) | 2.059(4) |
| Ni(1)-O(10) | 2.066(4) | Ni(2)-O(20) | 2.078(4) | Ni(3)-O(9) | 2.08(1) | Ni(4)-N(5) | 2.054(5) | Ni(5)-O(65) | 2.062(4) |
| Ni(1)-N(1) | 2.097(6) | Ni(2)-O(55) | 2.094(3) | Ni(3)-N(3) | 2.087(5) | Ni(4)-O(7) | 2.080(4) | Ni(5)-O(63) | 2.107(4) |
| Ni(1)-O(38) | 2.128(4) | Ni(2)-N(2) | 2.095(4) | Ni(3)-Cl(8) | 2.447(5) | Ni(4)-O(8) | 2.123(4) | Ni(5)-Cl(8) | 2.351(5) |
|  |  |  |  | Ni(3)-Cl(7) | 2.555(2) | Ni(4)-Cl(8) | 2.420(5) | Ni(5)-Cl(7) | 2.511(2) |
|  |  |  |  |  |  |  |  |  |  |

| Mn(10)-Cl(1)-Mn(18) | 75.80(4) | Mn(10)-O(3)-Mn(14) | 114.80(16) | Mn(19)-O(8)-Ni(5) | 98.23(16) | Mn(16)-O(28)-Mn(13) | 105.39(15) |
| --- | --- | --- | --- | --- | --- | --- | --- |
| Mn(10)-Cl(1)-Mn(15) | 73.65(4) | Mn(15)-O(3)-Mn(9) | 101.10(14) | Mn(19)-O(8)-Ni(5) | 109.58(18) | Mn(16)-O(29)-Mn(17) | 103.37(15) |
| Mn(18)-Cl(1)-Mn(15) | 74.06(4) | Mn(10)-O(3)-Mn(9) | 101.89(14) | Mn(19)-O(8)-Ni(4) | 154.0(2) | Mn(18)-O(30)-Mn(11) | 102.73(18) |
| Mn(10)-Cl(2)-Mn(12) | 75.24(5) | Mn(14)-O(3)-Mn(9) | 102.16(16) | Mn(19)-O(8)-Ni(4) | 94.63(16) | Mn(18)-O(31)-Mn(17) | 102.49(17) |
| Mn(10)-Cl(2)-Mn(14) | 74.15(4) | Mn(18)-O(4)-Mn(12) | 115.61(18) | Ni(5)-O(8)-Ni(4) | 100.01(15) | Mn(14)-O(32)-Mn(9) | 106.70(18) |
| Mn(12)-Cl(2)-Mn(14) | 74.25(5) | Mn(18)-O(4)-Mn(10) | 117.43(17) | Ni(5)-O(9)-Ni(4) | 104.9(4) | Mn(14)-O(33)-Mn(13) | 106.21(19) |
| Mn(18)-Cl(3)-Mn(16) | 76.13(4) | Mn(12)-O(4)-Mn(10) | 116.5(2) | Ni(5)-O(9)-Ni(3) | 117.5(5) | Ni(1)-O(38)-Mn(1) | 88.46(13) |
| Mn(18)-Cl(3)-Mn(12) | 75.36(5) | Mn(18)-O(4)-Mn(11) | 101.32(17) | Ni(4)-O(9)-Ni(3) | 95.7(4) | Mn(2)-O(39)-Mn(4) | 90.43(12) |
| Mn(16)-Cl(3)-Mn(12) | 73.78(5) | Mn(12)-O(4)-Mn(11) | 100.40(15) | Mn(1)-O(10)-Ni(1) | 105.19(19) | Mn(3)-O(41)-Mn(13) | 98.09(16) |
| Mn(16)-Cl(4)-Mn(15) | 76.49(4) | Mn(10)-O(4)-Mn(11) | 100.94(15) | Mn(1)-O(11)-Mn(8) | 99.14(18) | Mn(3)-O(43)-Mn(13) | 98.61(14) |
| Mn(16)-Cl(4)-Mn(14) | 74.35(4) | Mn(12)-O(5)-Mn(16) | 115.8(2) | Mn(2)-O(12)-Ni(1) | 118.65(18) | Mn(6)-O(50)-Mn(9) | 98.74(13) |
| Mn(15)-Cl(4)-Mn(14) | 74.24(4) | Mn(12)-O(5)-Mn(14) | 116.18(18) | Mn(3)-O(13)-Mn(2) | 95.30(15) | Mn(7)-O(54)-Mn(5) | 89.71(12) |
| Ni(5)-Cl(7)-Ni(3) | 87.14(6) | Mn(16)-O(5)-Mn(14) | 116.04(17) | Mn(4)-O(14)-Mn(3) | 89.60(16) | Ni(2)-O(55)-Mn(8) | 88.51(12) |
| Ni(5)-Cl(7)-Mn(19) | 75.16(5) | Mn(12)-O(5)-Mn(13) | 100.78(14) | Mn(4)-O(15)-Mn(5) | 99.27(18) | Ni(5)-O(63)-Mn(19) | 87.19(14) |
| Ni(3)-Cl(7)-Mn(19) | 80.35(5) | Mn(16)-O(5)-Mn(13) | 101.68(14) | Mn(5)-O(16)-Mn(4) | 100.17(18) | Mn(3)-O(1)-Mn(4) | 99.8(2) |
| Ni(5)-Cl(8)-Ni(4) | 84.35(15) | Mn(14)-O(5)-Mn(13) | 102.56(17) | Mn(5)-O(17)-Mn(6) | 90.72(16) | Mn(3)-O(1)-Mn(2) | 98.81(16) |
| Ni(5)-Cl(8)-Ni(3) | 93.42(18) | Mn(18)-O(6)-Mn(15) | 117.92(17) | Mn(7)-O(18)-Mn(6) | 95.41(16) | Mn(4)-O(1)-Mn(2) | 116.64(17) |
| Ni(4)-Cl(8)-Ni(3) | 77.81(14) | Mn(18)-O(6)-Mn(16) | 116.33(16) | Mn(7)-O(19)-Ni(2) | 119.04(16) | Mn(6)-O(2)-Mn(5) | 100.37(17) |
| Mn(6)-O(2)-Mn(7) | 99.29(15) | Mn(15)-O(6)-Mn(17) | 101.05(14) | Mn(8)-O(20)-Ni(2) | 104.60(17) | Mn(10)-O(24)-Mn(9) | 105.70(15) |
| Mn(5)-O(2)-Mn(7) | 115.91(18) | Mn(16)-O(6)-Mn(17) | 99.42(15) | Mn(8)-O(21)-Mn(1) | 98.65(18) | Mn(10)-O(25)-Mn(11) | 103.57(17) |
| Mn(15)-O(3)-Mn(10) | 117.10(18) | Mn(19)-O(7)-Ni(3) | 119.8(2) | Mn(15)-O(22)-Mn(17) | 103.71(15) | Mn(12)-O(26)-Mn(11) | 103.39(16) |
| Mn(15)-O(3)-Mn(14) | 116.07(16) | Mn(19)-O(7)-Ni(4) | 94.22(17) | Mn(15)-O(23)-Mn(9) | 105.51(15) | Mn(12)-O(27)-Mn(13) | 105.82(16) |
| Mn(15)-O(6)-Mn(16) | 115.39(18) | Ni(3)-O(7)-Ni(4) | 96.26(15) | Mn(18)-O(6)-Mn(17) | 101.99(17) | Mn(19)#0-O(8)-Mn(19) | 96.58(16) |

**Table S3.** Selected Interatomic Distances (Å) and angles (°) for (**2**)·2CH_3_CN·12.30H_2_O

| Mn(1)-O(1) | 2.217(5) | Mn(5)-O(30) | 1.947(5) | Mn(8)-O(4) | 2.129(6) | Mn(13)-O(43) | 1.885(5) | Mn(17)-O(22) | 1.852(5) |
| --- | --- | --- | --- | --- | --- | --- | --- | --- | --- |
| Mn(1)-O(3) | 2.200(7) | Mn(5)-O(32) | 2.202(5) | Mn(9)-O(45) | 1.865(6) | Mn(13)-O(44) | 1.865(5) | Mn(17)-O(25) | 2.347(5) |
| Mn(1)-O(5) | 2.165(7) | Mn(5)-O(33) | 1.981(6) | Mn(9)-Cl(4) | 2.603(2) | Mn(13)-O(53) | 1.906(5) | Mn(17)-O(23) | 1.931(6) |
| Mn(1)-O(7) | 2.330(6) | Mn(5)-O(41) | 1.885(7) | Mn(9)-Cl(1) | 2.732(2) | Mn(13)-O(56) | 1.924(5) | Mn(18)-O(52) | 1.922(5) |
| Mn(1)-O(40) | 2.446(5) | Mn(5)-O(52) | 1.854(6) | Mn(9)-O(53) | 1.893(6) | Mn(13)-Cl(2) | 2.698(3) | Mn(18)-O(28) | 1.932(6) |
| Mn(1)-O(48) | 2.205(6) | Mn(6)-O(33) | 2.497(6) | Mn(9)-O(55) | 1.924(6) | Mn(13)-Cl(1) | 2.718(3) | Mn(18)-O(29) | 1.862(5) |
| Mn(1)-O(54) | 2.278(5) | Mn(6)-O(32) | 2.265(5) | Mn(9)-O(45) | 1.864(6) | Mn(14)-O(2) | 2.144(5) | Mn(18)-O(30) | 1.925(6) |
| Mn(2)-O(6) | 1.896(7) | Mn(6)-O(42) | 2.210(8) | Mn(9)-O(46) | 1.833(7) | Mn(14)-O(43) | 2.125(5) | Mn(18)-O(31) | 2.181(5) |
| Mn(2)-O(7) | 2.191(5) | Mn(6)-O(44) | 2.204(5) | Mn(10)-O(3) | 1.872(5) | Mn(14)-O(50) | 2.132(7) | Mn(18)-O(26) | 2.225(5) |
| Mn(2)-O(9) | 1.956(5) | Mn(6)-O(45) | 2.236(7) | Mn(10)-O(4) | 1.882(7) | Mn(14)-O(56) | 2.171(5) | Ni(1)-O(17) | 2.090(6) |
| Mn(2)-O(38) | 2.188(5) | Mn(6)-O(47) | 2.213(5) | Mn(10)-O(54) | 1.898(6) | Mn(14)-Cl(5) | 2.338(3) | Ni(1)-O(10) | 2.036(5) |
| Mn(2)-O(40) | 1.991(6) | Mn(6)-O(53) | 2.303(5) | Mn(10)-O(55) | 1.943(5) | Mn(15-)O(8) | 2.194(5) | Ni(1)-O(12) | 2.022(7) |
| Mn(2)-O(51) | 1.882(7) | Mn(6)-O(47) | 2.213(5) | Mn(10)-Cl(3) | 2.642(3) | Mn(15)-O(9) | 1.952(6) | Ni(1)-O(14) | 2.099(5) |
| Mn(3)-O(39) | 2.177(6) | Mn(6)-O(53) | 2.303(5) | Mn(10)-Cl(4) | 2.683(3) | Mn(15)-O(10) | 1.860(6) | Ni(1)-O(15) | 2.036(7) |
| Mn(3)-O(13) | 2.367(5) | Mn(7)-O(47) | 1.874(6) | Mn(11)-O(55) | 1.911(6) | Mn(15)-O(11) | 1.937(7) | Ni(1)-N(1) | 2.056(7) |
| Mn(3)-O(36) | 1.949(6) | Mn(7)-O(48) | 1.877(5) | Mn(11)-O(56) | 1.905(6) | Mn(15)-O(13) | 2.237(5) | Ni(2)-N(2) | 2.093(6) |
| Mn(3)-O(37) | 1.915(5) | Mn(7)-O(54) | 1.930(6) | Mn(11)-O(49) | 1.863(7) | Mn(15)-O(51) | 1.933(6) | Ni(2)-O(22) | 2.061(5) |
| Mn(3)-O(38) | 1.877(7) | Mn(7)-O(53) | 1.934(5) | Mn(11)-O(50) | 1.893(6) | Mn(16)-O(14) | 2.419(5) | Ni(2)-O(24) | 2.074(6) |
| Mn(3)-O(51) | 1.906(5) | Mn(7)-Cl(2) | 2.685(3) | Mn(11)-Cl(1) | 2.621(3) | Mn(16)-O(16) | 1.907(7) | Ni(2)-O(25) | 2.106(5) |
| Mn(4)-O(26) | 2.383(5) | Mn(7)-Cl(4) | 2.713(3) | Mn(11)-Cl(3) | 2.681(3) | Mn(16)-O(17) | 1.866(6) | Ni(2)-O(27) | 2.028(5) |
| Mn(4)-O(34) | 2.209(6) | Mn(8)-O(46) | 2.172(7) | Mn(12)-O(1) | 1.852(5) | Mn(16)-O(18) | 1.904(6) | Ni(2)-O(29) | 2.021(5) |
| Mn(4)-O(35) | 1.848(6) | Mn(8)-O(49) | 2.157(8) | Mn(12)-O(2) | 1.893(5) | Mn(16)-O(19) | 2.169(5) |  |  |
| Mn(4)-O(36) | 1.916(5) | Mn(8)-O(55) | 2.151(6) | Mn(12)-O(54) | 1.913(5) | Mn(16)-O(21) | 1.928(5) |  |  |
| Mn(4)-O(37) | 1.943(6) | Mn(8)-O(57) | 2.312(5) | Mn(12)-O(56) | 1.925(5) | Mn(17)-O(18) | 1.950(5) |  |  |
| Mn(4)-O(52) | 1.914(5) | Mn(8)-O(46) | 2.172(7) | Mn(12)-Cl(2) | 2.656(3) | Mn(17)-O(20) | 2.167(6) |  |  |
| Mn(5)-O(35) | 2.179(5) | Mn(8)-O(49) | 2.157(8) | Mn(12)-Cl(3) | 2.713(3) | Mn(17)-O(21) | 1.913(6) |  |  |

| Mn(12)-O(1)-Mn(1) | 105.4(3) | Mn(13)-O(44)-Mn(6) | 105.8(2) | Mn(11)-O(55)-Mn(9) | 117.0(3) | Mn(9)-O(53)-Mn(13) | 117.5(3) |
| --- | --- | --- | --- | --- | --- | --- | --- |
| Mn(12)-O(2)-Mn(14) | 103.3(3) | Mn(9)-O(45)-Mn(6) | 106.0(3) | Mn(11)-O(55)-Mn(10) | 115.2(3) | Mn(9)-O(53)-Mn(7) | 115.8(3) |
| Mn(10)-O(3)-Mn(1) | 106.4(3) | Mn(9)-O(46)-Mn(8) | 104.4(3) | Mn(9)-O(55)-Mn(10) | 115.6(3) | Mn(13)-O(53)-Mn(7) | 115.3(3) |
| Mn(10)-O(4)-Mn(8) | 104.2(3) | Mn(7)-O(47)-Mn(6) | 106.3(3) | Mn(11)-O(55)-Mn(8) | 101.9(3) | Mn(9)-O(53)-Mn(6) | 102.5(2) |
| Mn(2)-O(7)-Mn(1) | 98.2(2) | Mn(7)-O(48)-Mn(1) | 106.8(3) | Mn(9)-O(55)-Mn(8) | 102.1(3) | Mn(13)-O(53)-Mn(6) | 100.7(2) |
| Mn(15)-O(9)-Mn(2) | 95.2(3) | Mn(11)-O(49)-Mn(8) | 103.3(3) | Mn(10)-O(55)-Mn(8) | 101.3(2) | Mn(7)-O(53)-Mn(6) | 100.9(2) |
| Mn(15)-O(10)-Ni(1) | 118.9(3) | Mn(11)-O(50)-Mn(14) | 103.0(3) | Mn(11)-O(56)-Mn(13) | 116.6(3) | Mn(10)-O(54)-Mn(12) | 116.8(3) |
| Mn(15)-O(13)-Mn(3) | 89.48(18) | Mn(2)-O(51)-Mn(3) | 100.1(3) | Mn(11)-O(56)-Mn(12) | 117.4(3) | Mn(10)-O(54)-Mn(7) | 116.4(3) |
| Ni(1)-O(14)-Mn(16) | 87.68(19) | Mn(2)-O(51)-Mn(15) | 98.3(3) | Mn(13)-O(56)-Mn(12) | 115.7(3) | Mn(12)-O(54)-Mn(7) | 114.2(3) |
| Mn(16)-O(17)-Ni(1) | 104.8(3) | Mn(3)-O(51)-Mn(15) | 115.2(3) | Mn(11)-O(56)-Mn(14) | 101.1(2) | Mn(10)-O(54)-Mn(1) | 102.5(2) |
| Mn(16)-O(18)-Mn(17) | 99.4(3) | Mn(5)-O(52)-Mn(4) | 99.9(2) | Mn(13)-O(56)-Mn(14) | 100.2(2) | Mn(12)-O(54)-Mn(1) | 101.1(2) |
| Mn(17)-O(21)-Mn(16) | 99.9(3) | Mn(5)-O(52)-Mn(18) | 98.9(3) | Mn(12)-O(56)-Mn(14) | 101.2(2) | Mn(7)-O(54)-Mn(1) | 102.2(2) |
| Mn(17)-O(22)-Ni(2) | 105.7(2) | Mn(4)-O(52)-Mn(18) | 115.0(3) | Mn(3)-O(37)-Mn(4) | 100.0(3) | Mn(18)-O(30)-Mn(5) | 95.6(2) |
| Ni(2)-O(25)-Mn(17) | 88.81(17) | Mn(5)-O(32)-Mn(6) | 99.7(2) | Mn(3)-O(38)-Mn(2) | 90.9(3) | Mn(4)-O(36)-Mn(3) | 99.7(3) |
| Mn(18)-O(26)-Mn(4) | 89.1(2) | Mn(5)-O(33)-Mn(6) | 98.7(2) | Mn(2)-O(40)-Mn(1) | 100.2(2) |  |  |
| Mn(18)-O(29)-Ni(2) | 119.4(3) | Mn(4)-O(35)-Mn(5) | 91.1(2) | Mn(13)-O(43)-Mn(14) | 103.1(2) |  |  |

**Table S4.** Selected Interatomic Distances (Å) and angles (°) for compound (**3**)·3.84 EtOH·6H_2_O

| Mn(1)-O(35) | 1.850(8) | Mn(5)-O(41) | 1.885(6) | Mn(9)-O(18) | 2.185(7) | Mn(12)-Cl(4) | 2.621(3) | Mn(16)-O(6) | 2.292(6) |
| --- | --- | --- | --- | --- | --- | --- | --- | --- | --- |
| Mn(1)-O(37) | 1.933(7) | Mn(5)-O(1) | 1.893(6) | Mn(9)-O(47) | 2.204(6) | Mn(13)-O(51) | 1.886(6) | Mn(16)-O(22) | 2.503(6) |
| Mn(1)-O(32) | 1.954(10) | Mn(5)-O(42) | 1.930(6) | Mn(9)-O(54) | 2.218(7) | Mn(13)-O(52) | 1.888(6) | Co(1)-O(39) | 2.025(7) |
| Mn(1)-O(36) | 1.956(8) | Mn(5)-O(43) | 1.957(6) | Mn(9)-O(55) | 2.226(6) | Mn(13)-O(6) | 1.891(6) | Co(1)-O(11) | 2.034(10) |
| Mn(1)-O(7) | 2.142(8) | Mn(5)-O(20) | 2.202(6) | Mn(9)-O(16) | 2.262(6) | Mn(13)-O(5) | 1.968(6) | Co(1)-O(38) | 2.081(7) |
| Mn(1)-O(28) | 2.339(7) | Mn(5)-O(14) | 2.324(7) | Mn(9)-O(4) | 2.292(6) | Mn(13)-Cl(1) | 2.626(3) | Co(1)-O(10) | 2.100(10) |
| Mn(2)-O(38) | 1.833(8) | Mn(6)-O(44) | 1.882(6) | Mn(9)-O(19) | 2.503(6) | Mn(13)-Cl(4) | 2.673(3) | Co(1)-N(1) | 2.128(11) |
| Mn(2)-O(36) | 1.938(7) | Mn(6)-O(2) | 1.908(6) | Mn(10)-O(47) | 1.850(7) | Mn(14)-O(50) | 1.857(7) | Co(1)-O(13) | 2.169(7) |
| Mn(2)-O(37) | 1.953(9) | Mn(6)-O(43) | 1.924(6) | Mn(10)-O(48) | 1.885(6) | Mn(14)-O(49) | 1.874(6) | Co(2)-O(46) | 2.050(7) |
| Mn(2)-O(9) | 1.970(10) | Mn(6)-O(42) | 1.939(6) | Mn(10)-O(3) | 1.900(6) | Mn(14)-O(3) | 1.904(6) | Co(2)-O(30) | 2.063(7) |
| Mn(2)-O(8) | 2.151(8) | Mn(6)-O(21) | 2.186(6) | Mn(10)-O(4) | 1.909(6) | Mn(14)-O(6) | 1.908(6) | Co(2)-O(31) | 2.071(8) |
| Mn(2)-O(13) | 2.348(7) | Mn(6)-O(27) | 2.359(6) | Mn(10)-Cl(3) | 2.701(3) | Mn(14)-Cl(2) | 2.670(3) | Co(2)-O(35) | 2.078(8) |
| Mn(3)-O(39) | 1.855(7) | Mn(7)-O(46) | 1.859(6) | Mn(10)-Cl(2) | 2.726(3) | Mn(14)-Cl(4) | 2.747(3) | Co(2)-N(2) | 2.129(8) |
| Mn(3)-O(1) | 1.917(6) | Mn(7)-O(2) | 1.909(6) | Mn(11)-O(54) | 1.888(6) | Mn(15)-O(56) | 1.868(6) | Co(2)-O(28) | 2.168(7) |
| Mn(3)-O(12) | 1.949(8) | Mn(7)-O(29) | 1.944(7) | Mn(11)-O(4) | 1.901(6) | Mn(15)-O(55) | 1.871(6) | Co(3)-O(34) | 1.958(8) |
| Mn(3)-O(40) | 1.950(7) | Mn(7)-O(45) | 1.956(7) | Mn(11)-O(53) | 1.901(6) | Mn(15)-O(4) | 1.926(6) | Co(3)-O(53) | 1.983(7) |
| Mn(3)-O(15) | 2.170(7) | Mn(7)-O(26) | 2.155(6) | Mn(11)-O(5) | 1.963(6) | Mn(15)-O(6) | 1.931(6) | Co(3)-O(52) | 2.000(7) |
| Mn(3)-O(14) | 2.227(6) | Mn(7)-O(27) | 2.226(6) | Mn(11)-Cl(1) | 2.653(3) | Mn(15)-Cl(2) | 2.616(2) | Co(3)-N(3) | 2.087(8) |
| Mn(4)-O(1) | 1.870(6) | Mn(8)-O(2) | 1.881(6) | Mn(11)-Cl(3) | 2.668(3) | Mn(15)-Cl(1) | 2.787(3) | Co(3)-O(5) | 2.152(6) |
| Mn(4)-O(17) | 1.933(7) | Mn(8)-O(23) | 1.930(6) | Mn(12)-O(57) | 1.907(6) | Mn(16)-O(24) | 2.188(7) | Co(4)-O(48) | 2.028(7) |
| Mn(4)-O(40) | 1.944(7) | Mn(8)-O(45) | 1.956(7) | Mn(12)-O(3) | 1.912(6) | Mn(16)-O(50) | 2.195(6) | Co(4)-O(57) | 2.032(7) |
| Mn(4)-O(19) | 1.966(6) | Mn(8)-O(22) | 1.994(6) | Mn(12)-O(33) | 1.924(7) | Mn(16)-O(56) | 2.209(6) | Co(4)-O(49) | 2.053(7) |
| Mn(4)-O(41) | 2.202(6) | Mn(8)-O(44) | 2.185(6) | Mn(12)-O(5) | 1.938(6) | Mn(16)-O(51) | 2.235(7) | Co(4)-O(3) | 2.172(6) |
| Mn(4)-O(16) | 2.210(6) | Mn(8)-O(25) | 2.203(6) | Mn(12)-Cl(3) | 2.610(3) | Mn(16)-O(25) | 2.251(6) | Co(4)-Cl(5) | 2.285(3) |
|  |  |  |  |  |  |  |  |  |  |

| Mn(13)-Cl(1)-Mn(11) | 77.00(7) | Mn(10)-O(4)-Mn(15) | 115.7(3) | Mn(1)-O(37)-Mn(2) | 98.6(4) | Mn(14)-O(3)-Mn(12) | 118.2(3) |
| --- | --- | --- | --- | --- | --- | --- | --- |
| Mn(13)-Cl(1)-Mn(15) | 74.17(7) | Mn(11)-O(4)-Mn(9) | 102.7(3) | Mn(2)-O(38)-Co(1) | 107.2(4) | Mn(10)-O(3)-Co(4) | 98.3(3) |
| Mn(11)-Cl(1)-Mn(15) | 73.91(7) | Mn(10)-O(4)-Mn(9) | 100.2(3) | Mn(3)-O(39)-Co(1) | 119.5(3) | Mn(14)-O(3)-Co(4) | 98.4(3) |
| Mn(15)-Cl(2)-Mn(14) | 75.19(7) | Mn(15)-O(4)-Mn(9) | 101.3(3) | Mn(4)-O(40)-Mn(3) | 95.0(3) | Mn(12)-O(3)-Co(4) | 100.2(3) |
| Mn(15)-Cl(2)-Mn(10) | 74.85(7) | Mn(12)-O(5)-Mn(11) | 115.5(3) | Mn(5)-O(41)-Mn(4) | 90.0(3) | Mn(11)-O(4)-Mn(10) | 115.4(3) |
| Mn(14)-Cl(2)-Mn(10) | 74.39(8) | Mn(12)-O(5)-Mn(13) | 115.4(3) | Mn(5)-O(42)-Mn(6) | 100.0(3) | Mn(11)-O(4)-Mn(15) | 117.5(3) |
| Mn(12)-Cl(3)-Mn(11) | 77.36(8) | Mn(11)-O(5)-Mn(13) | 113.5(3) | Mn(6)-O(43)-Mn(5) | 99.6(3) | Mn(8)-O(22)-Mn(16) | 98.6(2) |
| Mn(12)-Cl(3)-Mn(10) | 75.21(8) | Mn(12)-O(5)-Co(3) | 120.7(3) | Mn(6)-O(44)-Mn(8) | 90.2(3) | Mn(8)-O(25)-Mn(16) | 100.5(3) |
| Mn(11)-Cl(3)-Mn(10) | 73.69(7) | Mn(11)-O(5)-Co(3) | 94.2(3) | Mn(7)-O(45)-Mn(8) | 94.2(3) | Mn(7)-O(27)-Mn(6) | 89.6(2) |
| Mn(12)-Cl(4)-Mn(13) | 77.15(8) | Mn(13)-O(5)-Co(3) | 94.2(2) | Mn(7)-O(46)-Co(2) | 119.0(3) | Co(2)-O(28)-Mn(1) | 88.1(3) |
| Mn(12)-Cl(4)-Mn(14) | 75.13(8) | Mn(13)-O(6)-Mn(14) | 116.9(3) | Mn(10)-O(47)-Mn(9) | 105.4(3) | Mn(1)-O(35)-Co(2) | 105.8(4) |
| Mn(13)-Cl(4)-Mn(14) | 73.32(7) | Mn(13)-O(6)-Mn(15) | 117.4(3) | Mn(10)-O(48)-Co(4) | 104.0(3) | Mn(2)-O(36)-Mn(1) | 98.3(4) |
| Mn(4)-O(1)-Mn(5) | 100.7(3) | Mn(14)-O(6)-Mn(15) | 114.3(3) | Mn(14)-O(49)-Co(4) | 103.7(3) | Mn(11)-O(53)-Co(3) | 101.9(3) |
| Mn(4)-O(1)-Mn(3) | 98.6(3) | Mn(13)-O(6)-Mn(16) | 103.2(3) | Mn(14)-O(50)-Mn(16) | 105.4(3) | Mn(11)-O(54)-Mn(9) | 105.9(3) |
| Mn(5)-O(1)-Mn(3) | 115.0(3) | Mn(14)-O(6)-Mn(16) | 100.1(2) | Mn(13)-O(51)-Mn(16) | 105.5(3) | Mn(15)-O(55)-Mn(9) | 105.6(3) |
| Mn(8)-O(2)-Mn(6) | 99.3(3) | Mn(15)-O(6)-Mn(16) | 100.8(3) | Mn(13)-O(52)-Co(3) | 102.0(3) | Mn(15)-O(56)-Mn(16) | 106.0(3) |
| Mn(8)-O(2)-Mn(7) | 98.3(3) | Co(1)-O(13)-Mn(2) | 88.5(2) | Mn(4)-O(16)-Mn(9) | 99.2(2) | Mn(10)-O(3)-Mn(14) | 118.1(3) |
| Mn(6)-O(2)-Mn(7) | 115.7(3) | Mn(3)-O(14)-Mn(5) | 89.8(2) | Mn(4)-O(19)-Mn(9) | 98.6(2) | Mn(10)-O(3)-Mn(12) | 116.5(3) |

**Table S5.** Selected Interatomic Distances (Å) and angles (°) for compound (**4**)

| Mn(1)-O(3) | 1.875(3) | Ni(1)-Cl(1) | 2.524(2) |
| --- | --- | --- | --- |
| Mn(1)-O(3) | 1.888(3) | Ni(2)-O(5) | 2.002(4) |
| Mn(1)-O(2) | 1.936(3) | Ni(2)-O(6) | 2.049(3) |
| Mn(1)-O(8) | 1.937(3) | Ni(2)-N(3) | 2.063(4) |
| Mn(1)-O(7) | 2.206(3) | Ni(2)-O(2) | 2.090(3) |
| Mn(1)-Cl(1) | 2.831(2) | Ni(2)-O(3) | 2.104(3) |
| Mn(1)-Ni(2) | 2.939(1) | Ni(2)-O(1) | 2.270(3) |
| Mn(1)-Ni(3) | 3.046(1) | Ni(3)-O(9) | 2.086(4) |
| Ni(1)-O(4) | 2.023(4) | Ni(3)-N(4) | 2.115(5) |
| Ni(1)-O(2) | 2.028(3) | Ni(3)-O(3) | 2.119(3) |
| Ni(1)-N(2) | 2.068(5) | Ni(3)-O(7) | 2.159(3) |
| Ni(1)-N(1) | 2.092(5) | Ni(3)-O(1) | 2.179(3) |
| Ni(1)-O(1) | 2.327(3) | Ni(3)-Cl(1) | 2.5063(14) |

| Ni(3)-Cl(1)-Ni(1) | 87.16(4) | Ni(1)-O(2)-Ni(2) | 96.06(14) |
| --- | --- | --- | --- |
| Ni(3)-Cl(1)-Mn(1) | 77.02(4) | Mn(1)-O(3)-Mn(1) | 95.63(14) |
| Ni(1)-Cl(1)-Mn(1) | 79.11(4) | Mn(1)-O(3)-Ni(2) | 153.57(17) |
| Ni(3)-O(1)-Ni(2) | 92.57(12) | Mn(1)-O(3)-Ni(2) | 94.67(14) |
| Ni(3)-O(1)-Ni(1) | 100.56(12) | Mn(1)-O(3)-Ni(3) | 99.20(14) |
| Ni(2)-O(1)-Ni(1) | 83.52(12) | Mn(1)-O(3)-Ni(3) | 112.42(15) |
| Mn(1)-O(2)-Ni(1) | 119.10(17) | Ni(2)-O(3)-Ni(3) | 99.23(13) |
| Mn(1)-O(2)-Ni(2) | 93.69(15) | Ni(3)-O(7)-Mn(1) | 88.49(12) |

**Table S6.** Bond valence sum calculations^a^ for the Mn/Ni ions of compound (**1**)

|  | **Mn(II)** | **Mn(III)** | **Mn(IV)** | **Ni(II)** |
| --- | --- | --- | --- | --- |
| **Mn1** | 3,22 | *2,95* | 3,09 | 2,49 |
| **Mn2** | 2,94 | *2,69* | 2,82 | 2,49 |
| **Mn3** | 2,86 | *2,62* | 2,75 | 2,45 |
| **Mn4** | 2,98 | *2,72* | 2,86 | 2,46 |
| **Mn5** | 2,97 | *2,71* | 2,85 | 2,45 |
| **Mn6** | 2,86 | *2,62* | 2,75 | 2,45 |
| **Mn7** | 2,94 | *2,69* | 2,83 | 2,50 |
| **Mn8** | 3,00 | *2,75* | 2,88 | 2,45 |
| **Mn9** | *1,66* | 1,52 | 1,60 | 1,28 |
| **Mn10** | 3,22 | *3,00* | 3,11 | 2,48 |
| **Mn11** | *2,01* | 1,90 | 1,95 | 1,52 |
| **Mn12** | 3,24 | *3,01* | 3,12 | 2,46 |
| **Mn13** | *1,79* | 1,64 | 1,72 | 1,38 |
| **Mn14** | 3,18 | *2,95* | 3,06 | 2,44 |
| **Mn15** | 3,22 | *2,99* | 3,11 | 2,48 |
| **Mn16** | 3,19 | *2,96* | 3,07 | 2,45 |
| **Mn17** | *1,95* | 1,84 | 1,89 | 1,49 |
| **Mn18** | 3,25 | *3,03* | 3,14 | 2,50 |
| **Mn19** | 3,18 | *2,93* | 3,06 | 2,45 |
| **Ni1** | 2,77 | 2,56 | 2,65 | *2,05* |
| **Ni2** | 2,77 | 2,56 | 2,64 | *2,04* |
| **Ni3** | 2,83 | 2,73 | 2,71 | *1,95* |
| **Ni4** | 2,83 | 2,70 | 2,72 | *2,06* |
| **Ni5** | 2,77 | 2,66 | 2,68 | *2,03* |

^a^ The underlined value is the one closest to the charge for which it was calculated. The oxidation state of a particular atom can be taken as the nearest whole number to the underlined value.

**Table S7.** Bond valence sum calculations^a^ for the Mn/Ni ions of compound (**2**)·2CH_3_CN·12.30H_2_O

|  | **Mn(II)** | **Mn(III)** | **Mn(IV)** | **Ni(II)** |
| --- | --- | --- | --- | --- |
| **Mn1** | *1,87* | 1,71 | 1,80 | 1,45 |
| **Mn2** | 3,21 | *2,93* | 3,08 | 2,48 |
| **Mn3** | 3,22 | *2,95* | 3,09 | 2,49 |
| **Mn4** | 3,24 | *2,96* | 3,11 | 2,51 |
| **Mn5** | 3,31 | *3,03* | 3,18 | 2,56 |
| **Mn6** | *1,81* | 1,66 | 1,74 | 1,40 |
| **Mn7** | 3,19 | *2,96* | 3,08 | 2,35 |
| **Mn8** | *1,63* | 1,49 | 1,57 | 1,26 |
| **Mn9** | 3,43 | *3,19* | 3,31 | 2,64 |
| **Mn10** | 3,27 | *3,04* | 3,16 | 2,51 |
| **Mn11** | 3,33 | *3,10* | 3,21 | 2,56 |
| **Mn12** | 3,27 | *3,04* | 3,15 | 2,51 |
| **Mn13** | 3,24 | *3,01* | 3,13 | 2,49 |
| **Mn14** | *2,02* | 1,90 | 1,95 | 1,54 |
| **Mn15** | 3,23 | *2,96* | 3,11 | 2,50 |
| **Mn16** | 3,28 | *3,00* | 3,15 | 2,54 |
| **Mn17** | 3,25 | *2,97* | 3,12 | 2,52 |
| **Mn18** | 3,32 | *3,04* | 3,19 | 2,57 |
| **Ni1** | 2,86 | 2,64 | 2,73 | *2,10* |
| **Ni2** | 2,79 | 2,58 | 2,66 | *2,06* |

^a^ The underlined value is the one closest to the charge for which it was calculated. The oxidation state of a particular atom can be taken as the nearest whole number to the underlined value.

**Table S8.** Bond valence sum calculations^a^ for the Mn/Co ions of compound (**3**)·3.84 EtOH·6H_2_O

|  | **Mn(II)** | **Mn(III)** | **Mn(IV)** | **Co(II)** | **Co(III)** |
| --- | --- | --- | --- | --- | --- |
| **Mn1** | 3,20 | *2,93* | 3,07 | 2,54 | 2,54 |
| **Mn2** | 3,19 | *2,92* | 3,07 | 2,54 | 2,54 |
| **Mn3** | 3,28 | *3,00* | 3,15 | 2,61 | 2,61 |
| **Mn4** | 3,19 | *2,92* | 3,07 | 2,54 | 2,54 |
| **Mn5** | 3,19 | *2,92* | 3,07 | 2,54 | 2,54 |
| **Mn6** | 3,21 | *2,93* | 3,08 | 2,55 | 2,55 |
| **Mn7** | 3,30 | *3,02* | 3,17 | 2,62 | 2,62 |
| **Mn8** | 3,13 | *2,87* | 3,01 | 2,49 | 2,49 |
| **Mn9** | *1,85* | 1,69 | 1,77 | 1,47 | 1,47 |
| **Mn10** | 3,31 | *3,07* | 3,19 | 2,60 | 2,63 |
| **Mn11** | 3,17 | *2,95* | 3,06 | 2,48 | 2,52 |
| **Mn12** | 3,17 | *2,96* | 3,06 | 2,48 | 2,52 |
| **Mn13** | 3,22 | *3,00* | 3,11 | 2,52 | 2,56 |
| **Mn14** | 3,32 | *3,08* | 3,20 | 2,60 | 2,64 |
| **Mn15** | 3,24 | *3,01* | 3,12 | 2,54 | 2,57 |
| **Mn16** | *1,86* | 1,70 | 1,79 | 1,48 | 1,48 |
| **Co1** | 2,61 | 2,42 | 2,49 | *2,11* | 2,11 |
| **Co2** | 2,58 | 2,39 | 2,47 | *2,08* | 2,08 |
| **Co3** | 2,56 | 2,37 | 2,44 | *2,06* | 2,06 |
| **Co4** | 2,43 | 2,29 | 2,36 | *1,88* | 1,94 |

^a^ The underlined value is the one closest to the charge for which it was calculated. The oxidation state of a particular atom can be taken as the nearest whole number to the underlined value.

**Table S9.** Bond valence sum calculations^a^ for the Mn/Ni ions of compound (**4**)

|  | **Mn(II)** | **Mn(III)** | **Mn(IV)** | **Ni(II)** |
| --- | --- | --- | --- | --- |
| **Mn1** | 3,17 | *2,92* | 3,05 | 2,45 |
| **Ni1** | 2,63 | 2,50 | 2,51 | *1,81* |
| **Ni2** | 2,62 | 2,43 | 2,50 | *1,92* |
| **Ni3** | 2,33 | 2,19 | 2,23 | *1,69* |

^a^ The underlined value is the one closest to the charge for which it was calculated. The oxidation state of a particular atom can be taken as the nearest whole number to the underlined value.

**
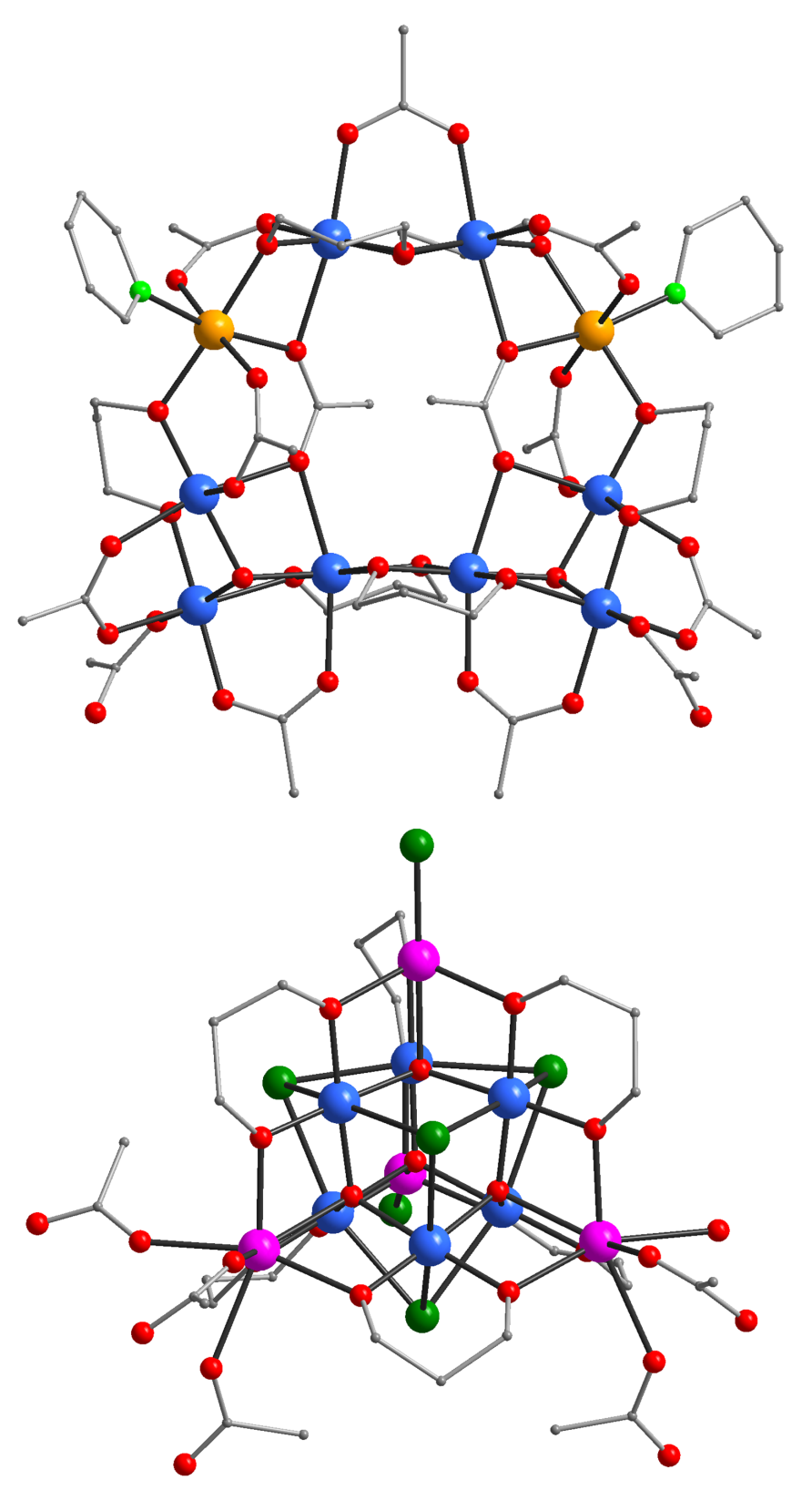
**

**Fig. S1**. Representations of the structures of the [Mn^III^_8_Ni_2_] loop (top) and the [Mn^III^_6_Mn^II^_4_] supertetrahedral (bottom) sub-units of (**1**). Color code: Mn^II^ purple, Mn^III^ blue, Ni^II^ orange, O red; N light green, Cl green, C gray. H atoms are omitted for clarity.

**
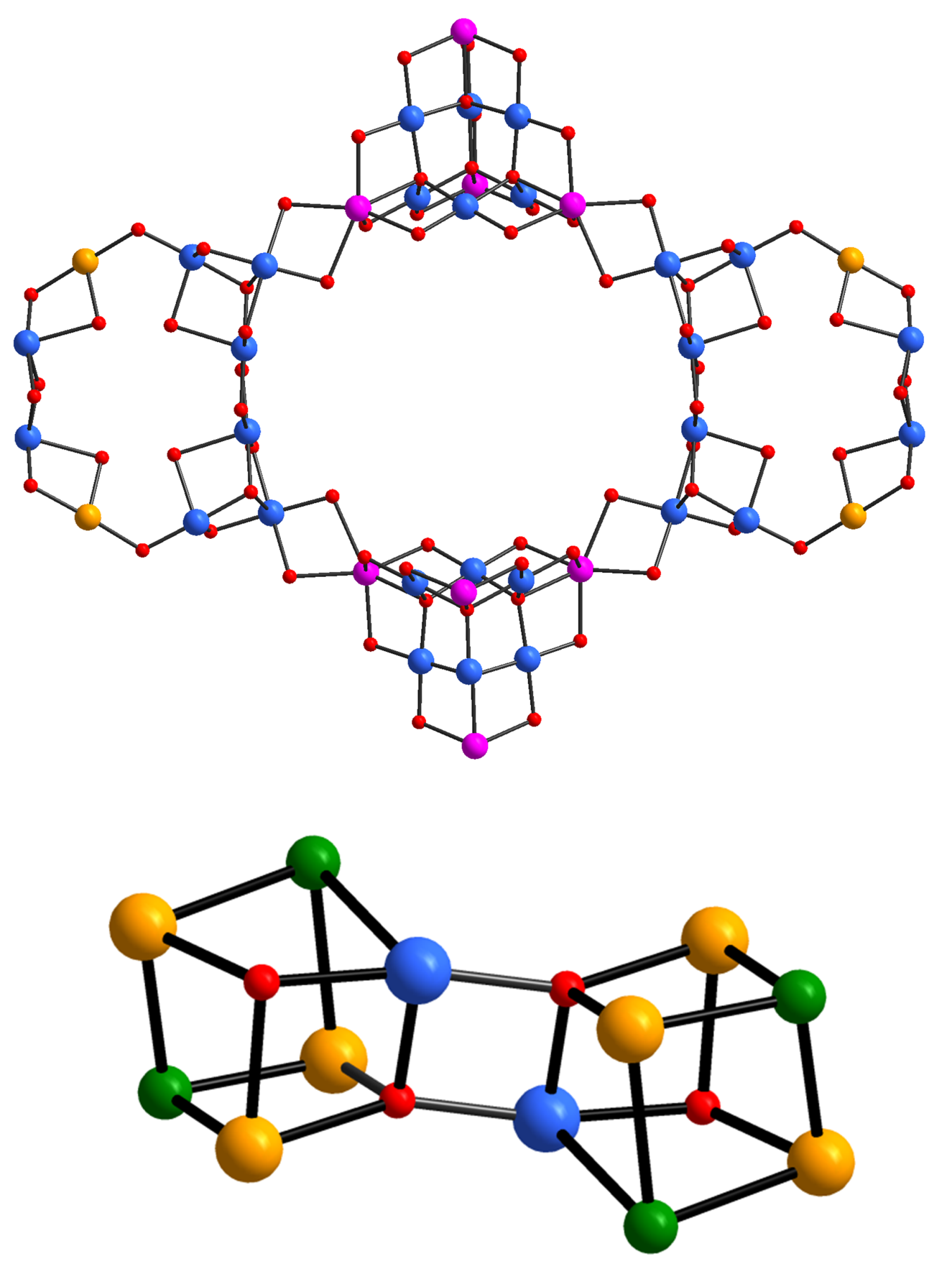
**

**Fig. S2**. Representation of the Mn/Ni/O core of the [Mn_36_Ni_4_]^2-^ anion (top) and the [Mn^III^_2_Ni^II^_6_]^2+^ cation (bottom) of (**1**). Color code: Mn^II^ purple, Mn^III^ blue, Ni^II^ orange, O red, Cl green.


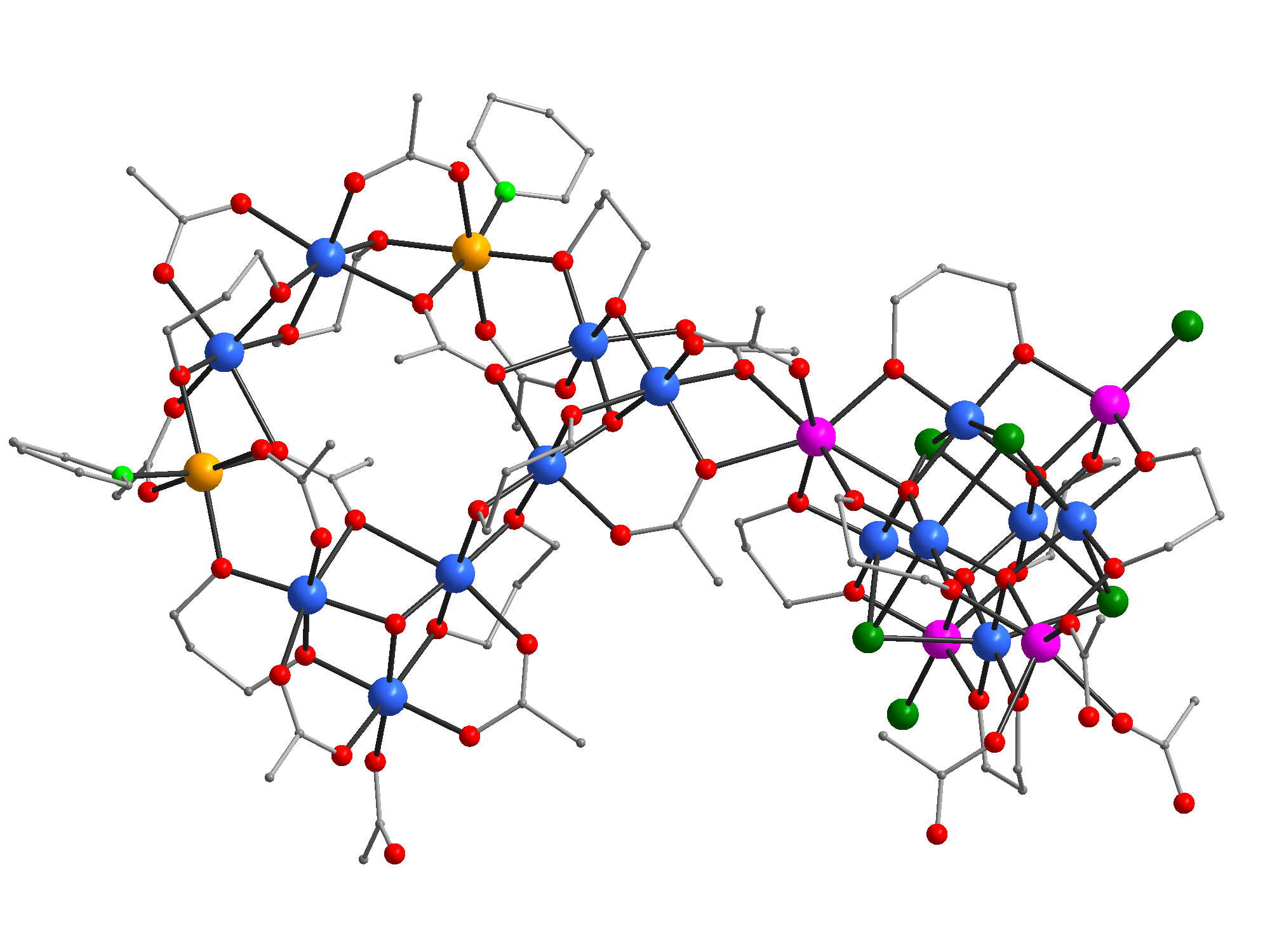


**Fig. S3**. Representation of a part of the molecular structure of the anion of (**1**) emphasizing on the connection of the loop and the supertetrahedral sub - units through acetate bridging ligands. Color code: Mn^II^ purple Mn^III^ blue, Ni^II^ orange, O red, N light green, Cl green, C gray. H atoms are omitted for clarity.


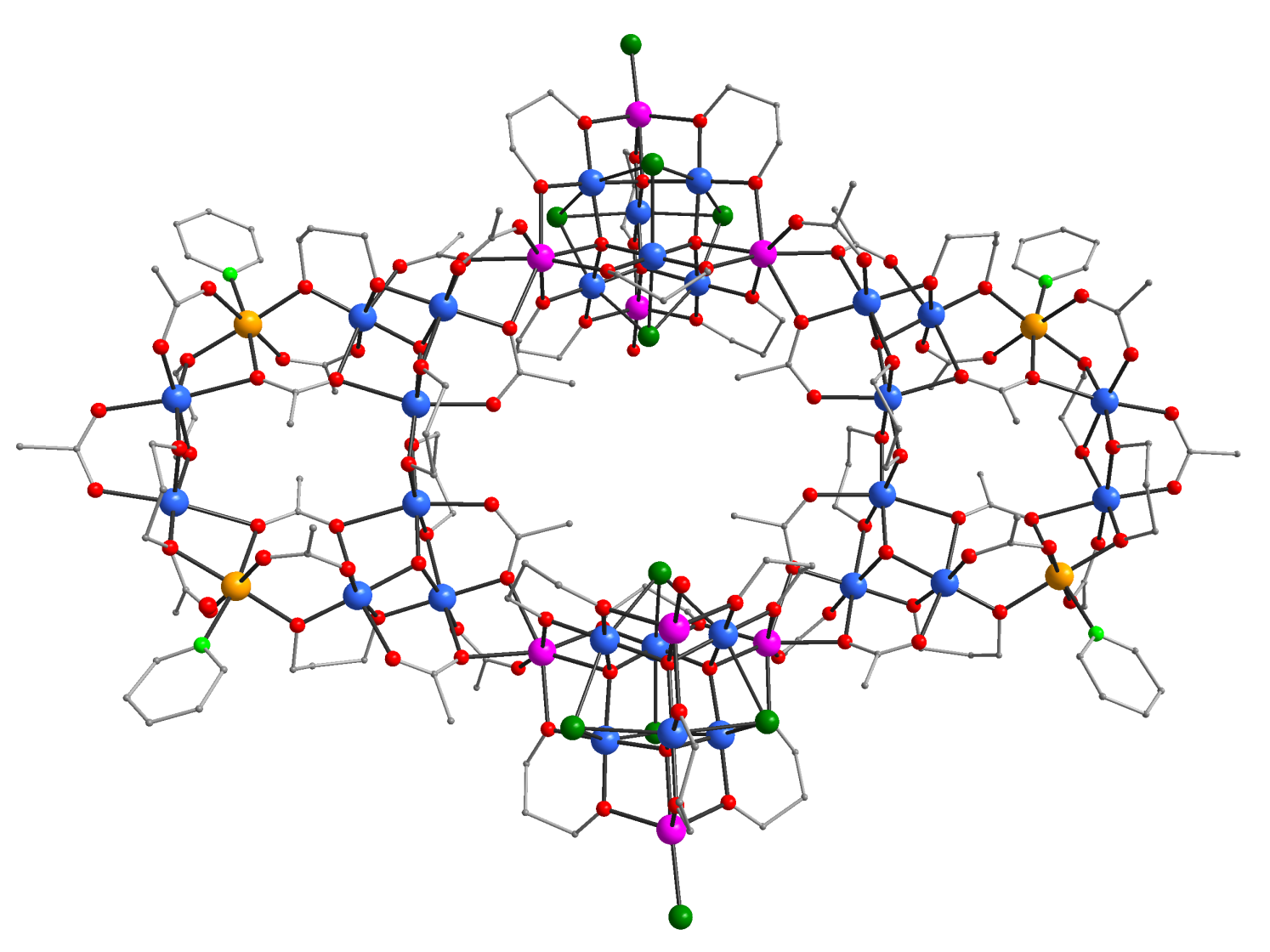


**Fig. S4**. Representation of the molecular structure of (**2**). Color code: Mn^II^ purple, Mn^III^ blue, Ni^II^ orange, O red, N light green, Cl green, C gray. H atoms are omitted for clarity.

**
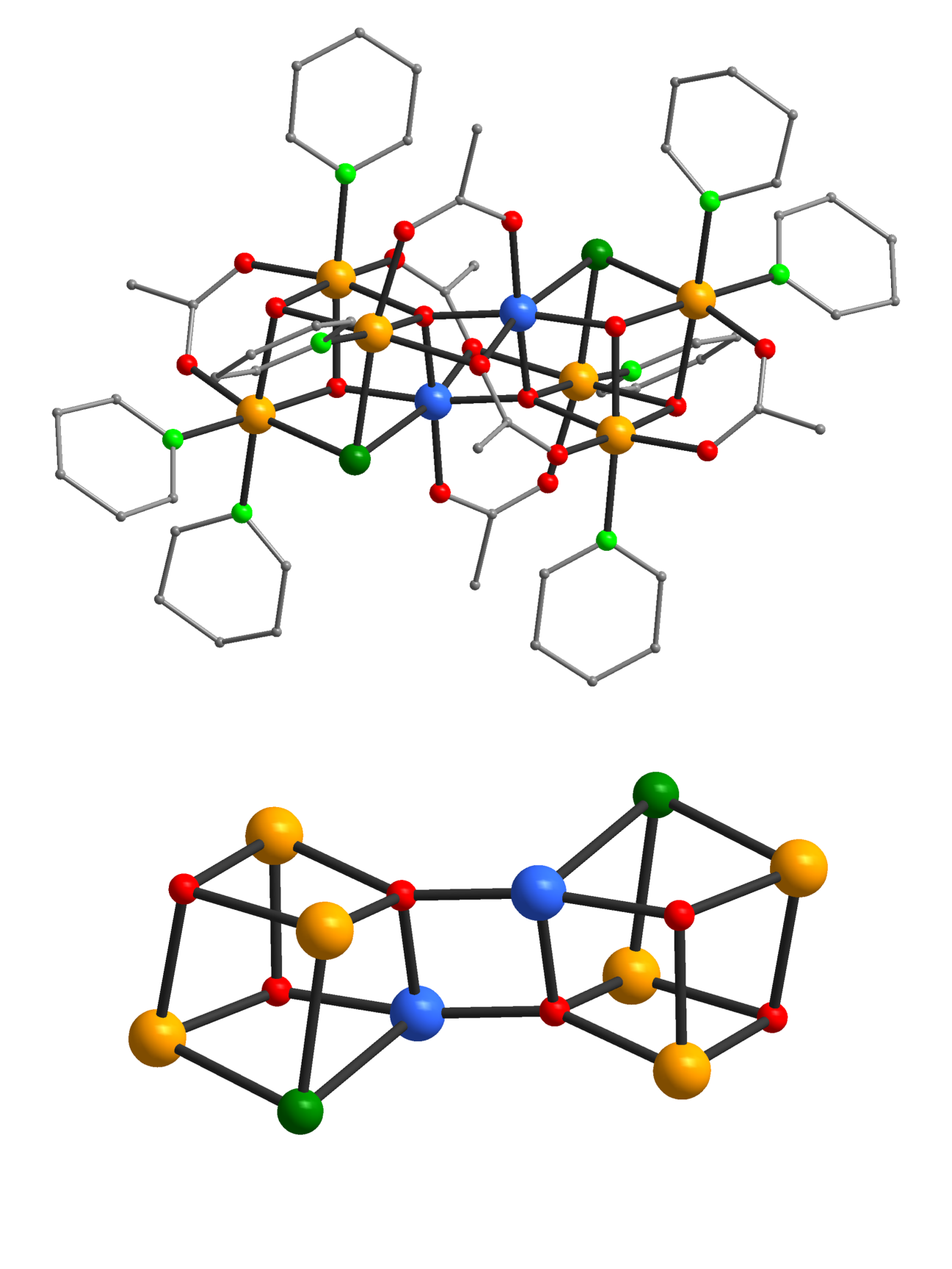
**

**Fig. S5**. Representations of the molecular structure (top) and the [Mn^III^_2_Ni^II^_6_(μ_4_-Ο)_2_(μ_3_-ΟH)_4_(μ_3_-Cl)_2_]^8+^ core (bottom) of (**4**). Color code: Mn^II^ purple, Mn^III^ blue, Ni^II^ orange, O red; N light green, Cl green, C gray. H atoms are omitted for clarity.

**Results/Magnetic Properties**


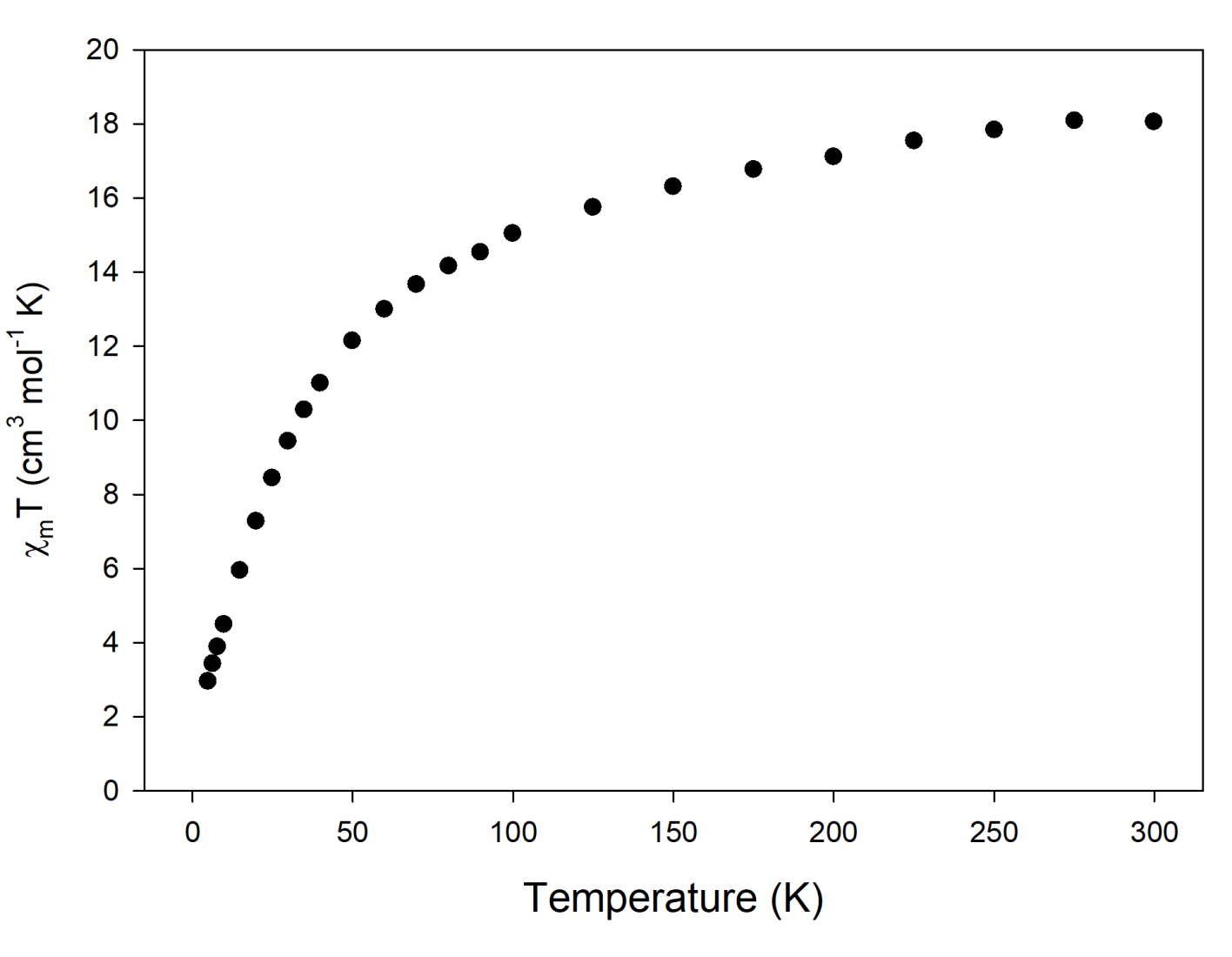


**Figure S6.** χ_M_T versus T plots for complex (**4**)·2 H_2_O in the temperature range 5–300 K in a 0.1 T applied dc field.

**
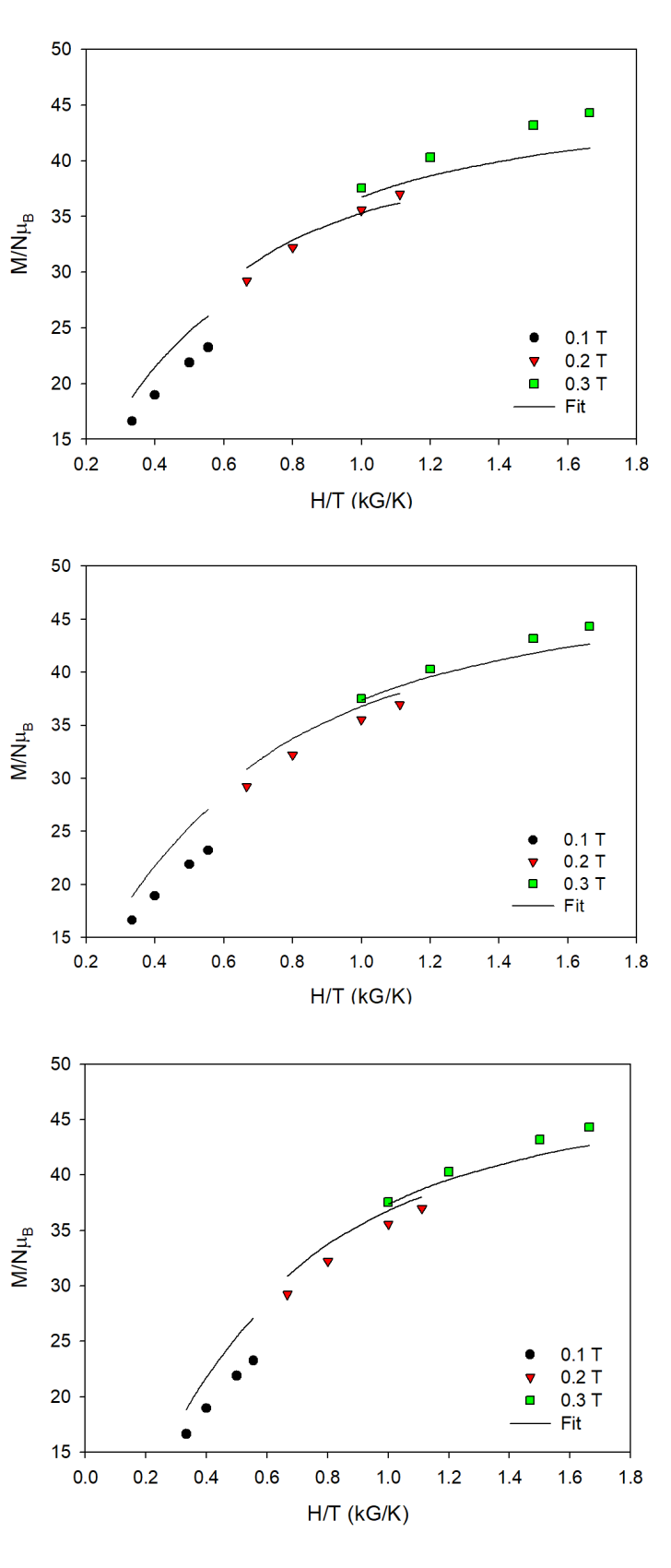
**

**Fig. S7**. Plots of reduced magnetisation (*M*/*Nμ*_B_) *vs* *H*/*T* for complex (**2**)·10H_2_O at the indicated fields for spin ground state values *S_T_* =25 (top), 26 (middle) and 27 (bottom). The solid lines are the fit of the data; see the text for the fitting parameters


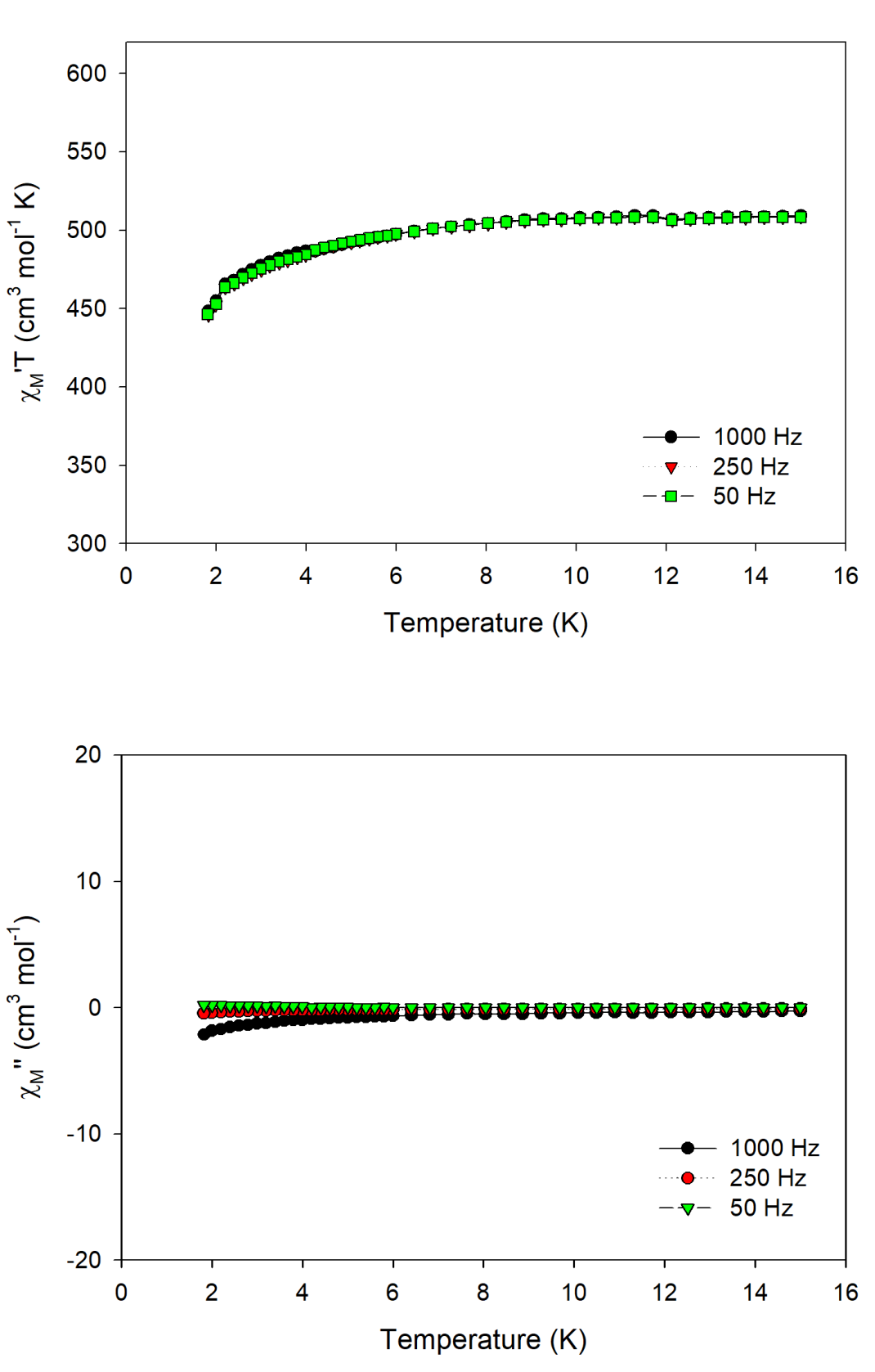


**Figure S8.** Plots of the in-phase (χ_M_') signal as χ_M_'*T vs. T* (top) and the the out-of-phase (χ_M_*''*) signal as χ_M_*''* *vs. T* (bottom) in a 3.5 G field oscillating at the indicated frequencies for complex (**1**)·6H_2_O.

**
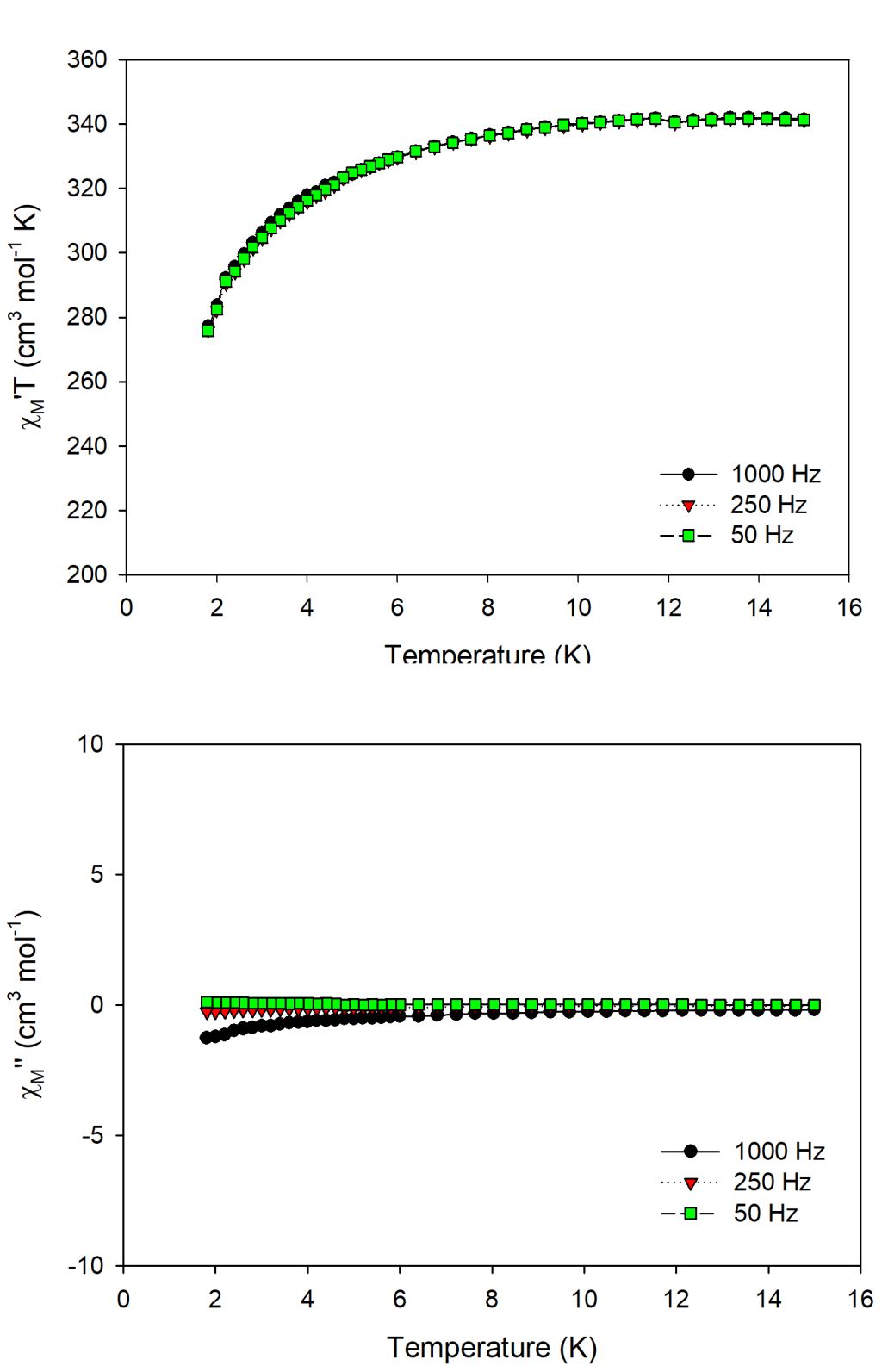
**

**Figure S9.** Plots of the in-phase (χ_M_') signal as χ_M_'*T vs. T* (top) and the the out-of-phase (χ_M_*''*) signal as χ_M_*''* *vs. T* (bottom) in a 3.5 G field oscillating at the indicated frequencies for complex (**2**)·10H_2_O.

**
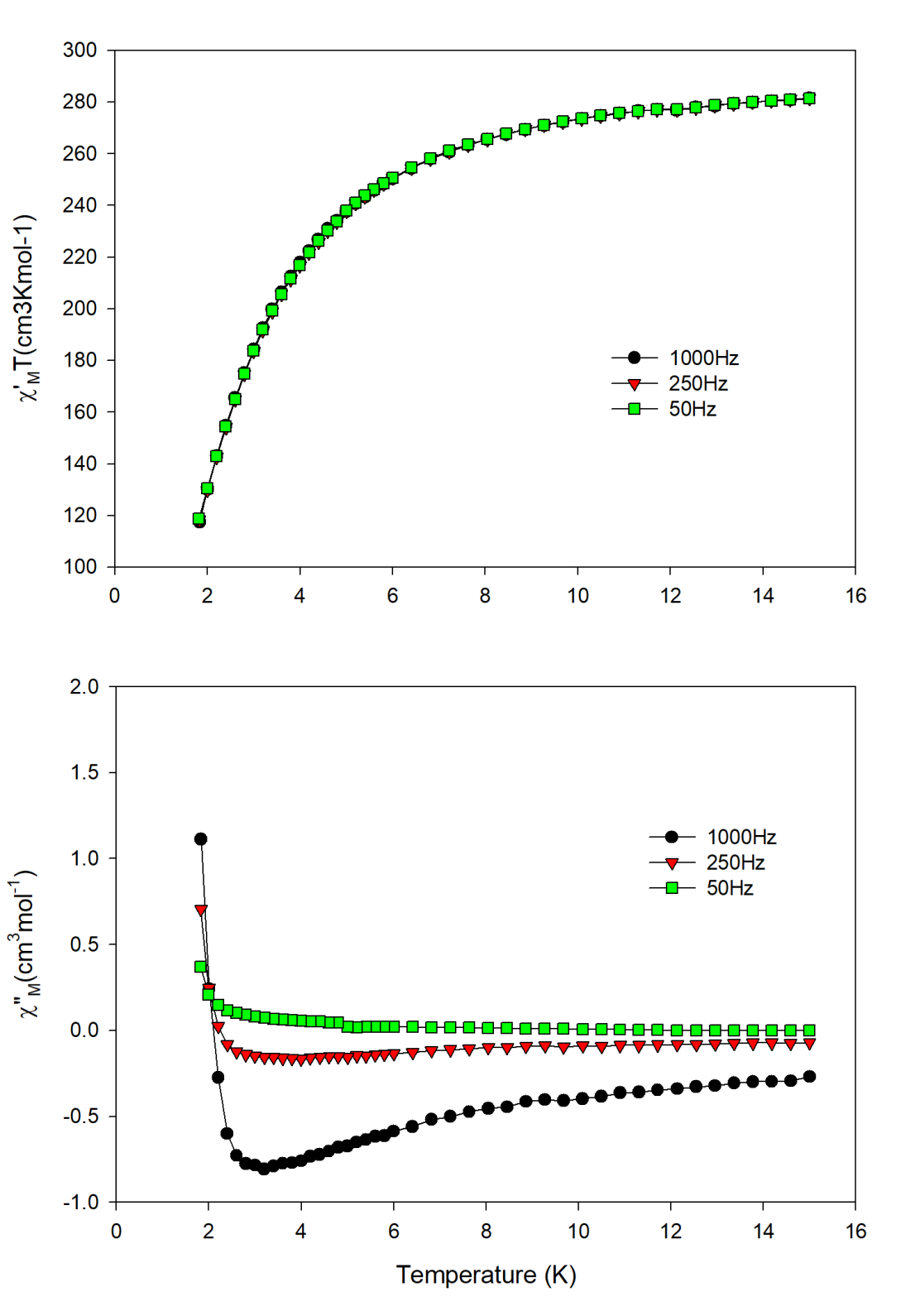
**

**Figure S10.** Plots of the in-phase (χ_M_') signal as χ_M_'*T vs. T* (top) and the the out-of-phase (χ_M_*''*) signal as χ_M_*''* *vs. T* (bottom) in a 3.5 G field oscillating at the indicated frequencies for complex (**3**)·20H_2_O.
